# Coumarin Compounds with 6- or 7-(4-Aminobutynyloxy) Substituent: Synthesis, In Vitro Cytotoxicity and In Silico DNA Binding Studies

**DOI:** 10.3390/molecules31183222

**Published:** 2026-09-12

**Authors:** Anarkul S. Kishkentayeva, Mohammad S. Hamad, Victor A. Savelyev, Zhasmin A. Boyaubayeva, Andrey I. Khlebnikov, Andrey G. Pokrovsky, Yurii V. Gatilov, Almagul S. Makhmutova, Elvira E. Shults

**Affiliations:** 1 School of Pharmacy, Karaganda Medical University, Karaganda 100012, Kazakhstan; bayaubaeva.zhasmin@gmail.com (Z.A.B.); almagul_312@mail.ru (A.S.M.); 2 Institute of Medicine and Medical Technologies, Novosibirsk State University, Pirogova Str., 1, 630090 Novosibirsk, Russia; khamad@g.nsu.ru (M.S.H.); agpok@inbox.ru (A.G.P.); 3 N.N. Vorozhtsov Novosibirsk Institute of Organic Chemistry, Siberian Branch of the Russian Academy of Sciences, 630090 Novosibirsk, Russia; vicsav@nioch.nsc.ru (V.A.S.); gatilov@nioch.nsc.ru (Y.V.G.); 4 Kizhner Research Center, Tomsk Polytechnic University, 634050 Tomsk, Russia; aikhl@chem.org.ru

**Keywords:** coumarins, umbelliferone, prop-2-ynyloxycoumarins, A^3^-coupling reaction, cytotoxicity, DNA, G-quadruplex

## Abstract

Coumarin compounds are of great interest in drug development research. Various substituents on the coumarin core significantly influence its biological activity. Although a number of coumarins with specific biological properties have already been identified, the ongoing challenge lies in the design and synthesis of novel derivatives with high specificity for pharmacological targets. In this work, 6-(4-aminobut-2-ynyloxy)- and 7-(4-aminobut-2-ynyloxy)-substituted coumarin derivatives were designed and synthesized. As a method for the synthesis of 6- or 7-substituted coumarins (yields 26–98%), a copper-catalyzed one-pot three-component reaction (A^3^ coupling) of 6- or 7-(prop-2-ynyloxy)coumarins with formaldehyde and secondary amines was studied. In vitro biological testing (MTT assay) results showed that the new coumarins exhibit pronounced cytotoxicity against human cervical cancer (C33 A and CaSki) and breast cancer (MCF-7) cell lines, with activity being dependent on the substituent at the nitrogen atom in the side chain. The most active compounds inhibited tumor cell growth, with GI_50_ values of 4.3–9.4 μM (SI = 9.1–19.8). All new compounds demonstrated low cytotoxicity against the non-malignant epithelial VERO cells (GI50 > 86 μM). To understand the observed SAR trends, molecular modeling of the interaction between the new coumarin derivatives and DNA G-quadruplex binding sites was performed.

## 1. Introduction

Coumarins have been recognized as a varied class of naturally occurring pharmacophores for the design and development of bioactive compounds with therapeutic and agrochemical applications [1]. Numerous coumarins of natural origin have demonstrated promising antitumor activity both in vitro and in vivo. The coumarin structural scaffold (2*H*-1-benzopyran-2-one) is well-known for its broad biological activity, including antibacterial [2], antiviral (anti-HIV) [3], neuroprotective [4], anti-inflammatory [5], and antitumor [6] effects. Due to this wide spectrum of biological action, the design and synthesis of new coumarin-based molecules remain a focal point in the search for novel drugs. To overcome tumor cell resistance mechanisms and target various biological pathways, coumarin-based hybrid molecules have been developed [7,8]. Biological studies have shown that the antitumor effect of coumarins is due to their involvement in multiple signaling pathways. They affect a range of processes associated with cancer progression, including kinase inhibition, cell cycle arrest, suppression of heat shock proteins (HSP90), angiogenesis, monocarboxylate transporters, telomerase, aromatase, and sulfatase, as well as exhibiting antimitotic activity and inhibiting carbonic anhydrase.

**Figure 1:**
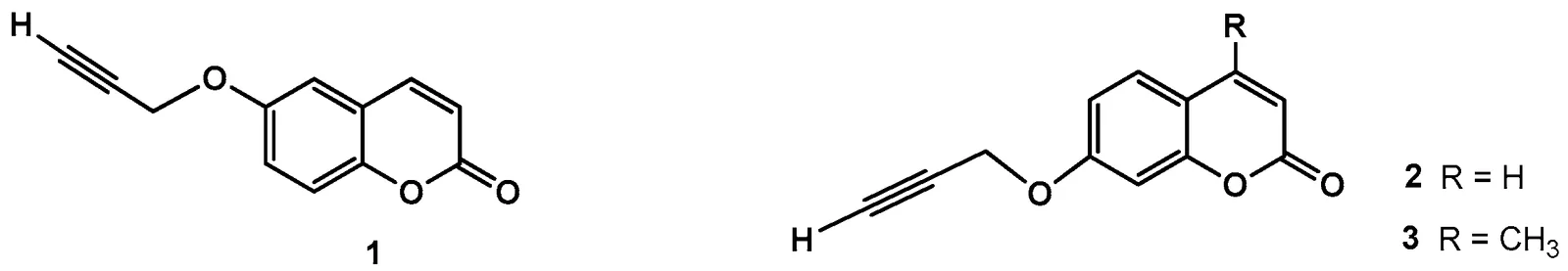
Structures of propargyloxysubstituted coumarins **1**–**3**.

The coumarin core, endowed with a large synthetic accessibility and favorable pharmacokinetic properties, appears as a valuable, privileged scaffold to be properly modified in order to obtain compounds that are able to engage different selected targets [9,10]. The acetylene is a privileged structural motif that can target many important proteins [11], for example, as a rigid linker incorporated into kinase inhibitors very successfully [12,13]. In this area, coumarin derivatives with alkynyl or alkynyloxy substituents on the aromatic ring are noteworthy. Therefore, alkynyl-coumarinyl ethers were developed as inhibitors of human monoamine oxidase B (MAO-B). A hex-5-ynyloxy chain at position 7 was found to be particularly advantageous [14]. 6-Propargyloxy-substituted coumarin **1** (Figure 1) showed selective medium-potency inhibition of the tumor-associated human carbonic anhydrase XII isoform (K_I_ = 56.4 nM), whereas its regioisomer at the 7 position (compound **2**) resulted in being 12.9-fold less potent (K_I_ = 730.0 nM) [15]. Compound **1** was characterized as the most promising in counteracting the increase in cellular metabolic activity induced by LPS. On the other hand, compound **2** was found to be very effective in decreasing LPS-induced H_2_O_2_ generation (via an in vitro biological evaluation on rat astrocytes). Addition of the methyl group to the C-4 position of the coumarin nucleus (compound **3**) contributed to its superior activity in the MPP+-induced neurotoxicity model in vitro [16].

Based on the aforementioned results concerning the activity of alkynyl-substituted coumarins, and in continuation of our research on the structural modification of coumarins [17,18,19,20,21], we directed this work toward the synthesis and evaluation of two types of isomeric coumarin derivatives containing an aminobut-2-ynyloxy substituent at the C-6 or C-7 positions. We hypothesized that modifying the coumarin core by hybridization with nitrogen-containing pharmacophoric fragments via a rigid acetylene linker could be a promising strategy for reducing side effects, overcoming drug resistance, and achieving effective therapeutic outcomes in cancer treatment. We focused on the study of the three-component one-pot copper-catalyzed Mannich reaction of 6-(prop-2-ynyloxy)-2*H*-chromen-2-one **1** and 7-(prop-2-ynyloxy)-2*H*-chromen-2-one **2**, **3** with formaldehyde and secondary amines (A^3^ coupling). This aldehyde–alkyne–amine A^3^-coupling reaction has become a powerful synthetic method due to its atom-economical nature and the possibility of assembling molecules with high levels of diversity and complexity [22,23]. The formation of the propynylamino moiety in this type of reaction was reported to proceed fast and smoothly and, as such, has found contrasting applications in medicinal chemistry, biology, and materials science for the generation of relevant linker groups [24,25,26]. By reaction of 6- or 7-(prop-2-ynyloxy)coumarins, formaldehyde, and various secondary amines (dialkylamines, azepane, azocane, N-substituted piperazines, 1,2,3,4-tetrahydroisoquinoline), we constructed an original library of 6- or 7-(4-aminobut-2-ynyloxy) substituted 2*H*-chromen-2-ones. Taking into account the interest in 6-hydroxy- and 7-hydroxy-substituted coumarin derivatives as anticancer agents [27,28,29,30], the new series of modified coumarins was evaluated for their in vitro anticancer activity against human cervical cancer (C33 A and CaSki) and breast cancer (MCF-7) cell lines using the conventional MTT assay. All compounds were non-toxic towards normal epithelial VERO cells and showed differential effects against the tested cell lines, exhibiting a greater impact and selectivity against the breast carcinoma cell line MCF-7. The molecular docking of new compounds to G-quadruplex DNA motifs was carried out.

## 2. Results

### 2.1. Chemical Synthesis

**Scheme 1:**
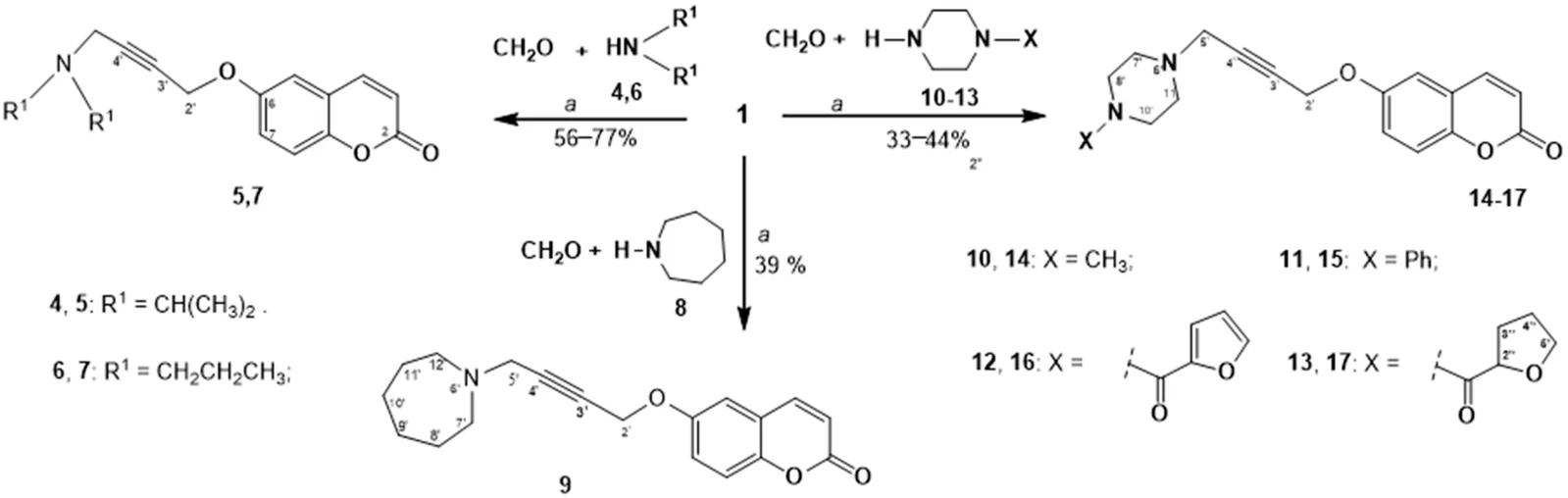
Synthesis of N-substituted 6-(4-(amino)but-2-ynyloxy)chromen-2-ones. Reagents and conditions: (a) Cu(OAc)_2_·H_2_O, dioxane, 65–70 °C, 3–5 h.

We found that the reaction of 6-(prop-2-ynyloxy)coumarin **1**, aq. formaldehyde (4 equiv.), and diisopropylamine **4** (1.8 equiv.), in the presence of copper(II) acetate monohydrate (5% mol) in 1,4-dioxane, proceeds smoothly, and by heating at 70 °C for 5 h, the alkyne was almost consumed. After treatment of the reaction mixture and column chromatography, the targeted 6-(4-(diisopropylamino)but-2-ynyloxy)-2*H*-chromen-2-one **5** was isolated as a white powder in good yield (77%) (Scheme 1). The copper salts induced an A^3^-coupling reaction of compound **1** with propylamine **6** (65 °C, 6 h) and formaldehyde, providing target compound **7** in a 56% yield. Secondary cyclic amine azepane **8** was also employed as a substrate, affording the targeted 6-(4-(azepan-1-yl)but-2-ynyloxy)-2*H*-chromen-2-one **9** (yield 33%). Copper salt-mediated coupling of **1** with formaldehyde and N-substituted piperazines **10**–**13** resulted in targeted N-substitutes 6-(4-(N-piperazin-1-yl)but-2-ynyloxy)-2*H*-chromen-2-ones **14**–**17** in moderate isolated yields (33–44%).

**Scheme 2:**
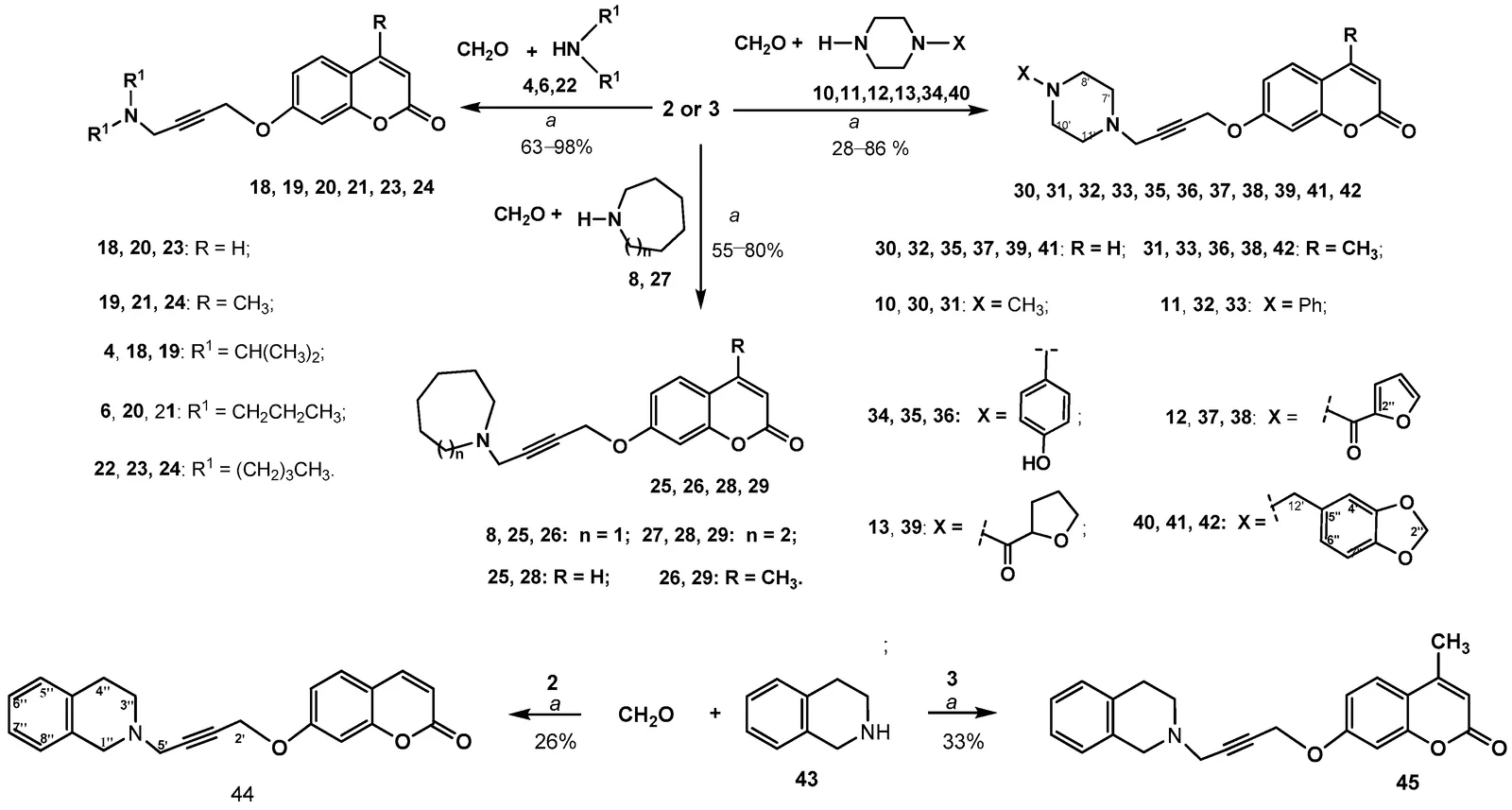
Synthesis of N-substituted 7-(4-(amino)but-2-ynyloxy)chromen-2-ones. Reagents and conditions: (a) Cu(OAc)_2_·H_2_O, dioxane, 65–70 °C, 3–5 h.

The three-component reaction of 7-*O*-propynylumbelliferon **2** with dialkylamines **4**, **6**, **22**, and aq. formaldehyde in the presence of Cu(OAc)_2_·H_2_O resulted in 7-(4-dialkylaminobut-2-ynyloxy)coumarins **18**, **20**, and **23** in good to excellent yields (63, 68, and 98%, respectively) (Scheme 2). The reaction of 7-*O*-propynylhymecromone **3** with aq. formaldehyde and acyclic secondary amines **4**, **6**, and **22** (65 °C, 3 h) led to the target hymechromone derivatives **19**, **21**, and **24** in very good, isolated yields (74–93%). Reaction of cyclic secondary amines: azepane **8** and azocane **27** with aq. formaldehyde and 7-(prop-2-ynyloxy)coumarins **2** and **3** in the presence of Cu(OAc)_2_·H_2_O required a longer duration and resulted in the formation of 7-(4-(azepan-1-yl)but-2-ynyloxy)-2*H*-chromen-2-ones **25** and **26** (yield 55–71%) or 7-(4-(azocan-1-yl)but-2-ynyloxy)-2*H*-chromen-2-ones **28** and **29** (yield 57–80%). Several N-substituted piperazines **10**–**13**, **34**, and **40** were introduced in the reaction with formaldehyde and alkynyl umbelliferone **2** or alkynyl hymechromone **3** to afford the expected 7-(4-(4-R-piperazin-1-yl)but-2-ynyl)coumarin derivatives **30**–**33**, **35**–**38**, **41**, and **42** in satisfactory to excellent yields (39–86%). A significant decrease in the yield of the target product **39** (down to 28%) was observed in the reaction of piperazin-1-yl(tetrahydrofuran-2-yl)methanone **13** with formaldehyde and 7-*O*-propynyl-substituted umbelliferone **2**. The reaction of propynyl-substituted coumarins **2** or **3** with formaldehyde and 1,2,3,4-tetrahydroisoquinoline **43** resulted in the formation of hybrid compounds **44** or **45** containing isoquinoline and chromene heterocycles linked by a rigid butynyloxy group (Scheme 2).

**Figure 2:**
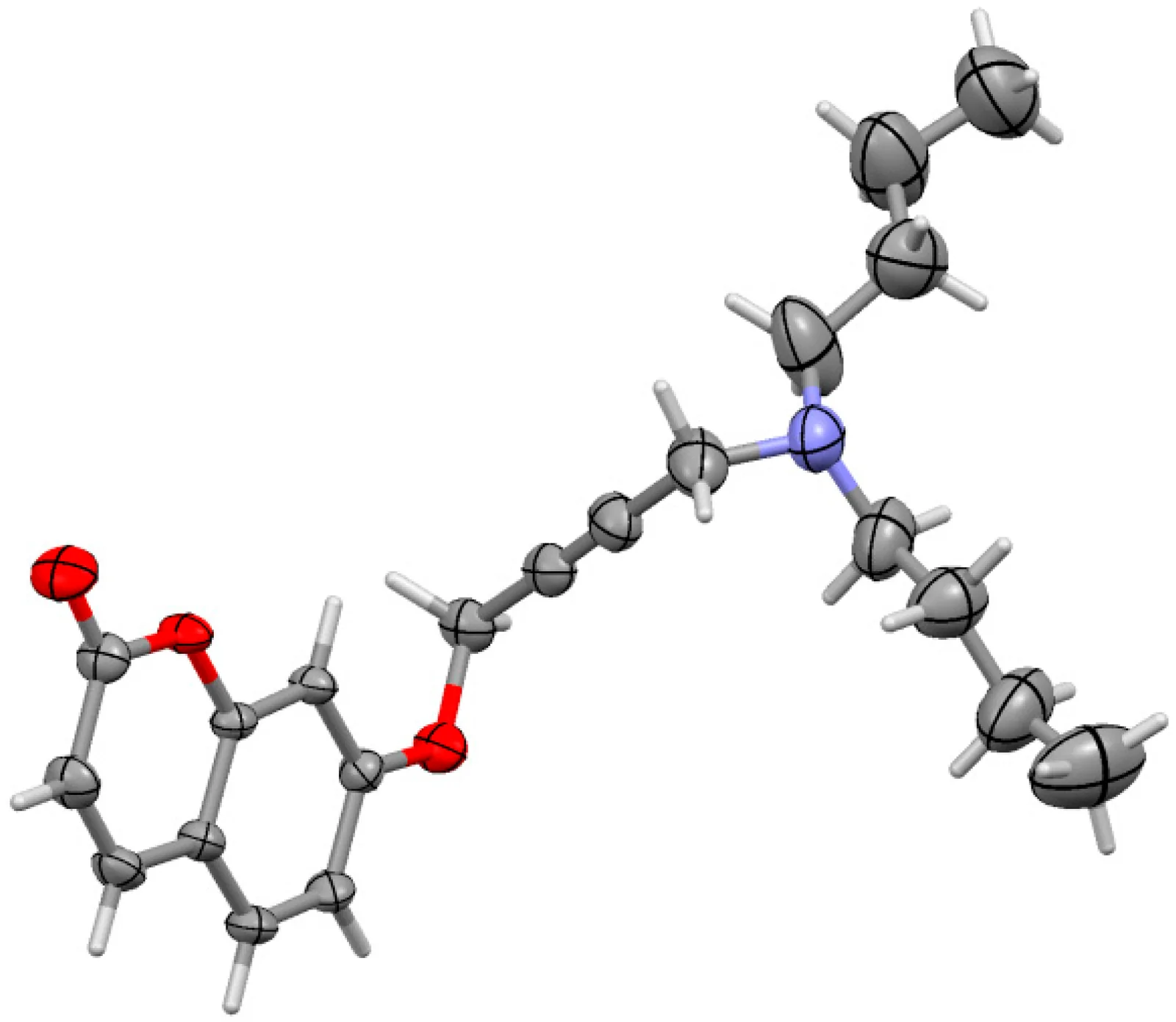
X-ray crystal structure of 7-(4-(dibutylamino)but-2-ynyloxy)-2*H*-chromen-2-one **23** (CCDC 2498361).

The structures of the novel compounds were confirmed by ^1^H and ^13^C-NMR, IR, UV, and HR-MS data. The ^1^H and ^13^C NMR spectra of 6- and 7-substituted coumarin derivatives (recorded in CDCl_3_ or (CD_3_)_2_SO) were in good agreement with their structure and contained one set of characteristic signals of the coumarin core, the corresponding substituents, and the butynyloxy linker group (Supplementary Figures S1–S60). In particular, the ^1^H NMR spectra of **5**, **7**, **9**, **14**–**21**, **23**–**33**, **35**–**39**, **41**, **42**, **44**, and **45** contain the resonance signals of the CH_2_ groups (H-2′, 5′), which were presented as broad singlets of two protons in the range of 4.85–4.70 (H-2′) and 3.41–3.30 (H-5′) ppm. The IR spectra of all final compounds **5**, **7**, **9**, **14**–**21**, **23**–**33**, **35**–**39**, **41**, **42**, **44**, and **45** showed a C≡C stretching vibration around 2285–2219 cm^−1^ (as broad absorption bands). In the ^13^C NMR spectra, the signals of the triple-bond carbon atoms appear at δC 76.72–79.92 (C-3′) and δC 82.47–88.04 (C-4′) ppm. These chemical shift values are characteristic of di-substituted alkynes. All synthesized compounds had UV spectra characteristic of coumarins. For example, the spectrum of **38** had absorption bands with maxima at 290 (lgε = 3.90) and 325 (lgε = 4.14) nm. The ESI mass spectrometric data of all synthesized compounds showed peaks at relevant [M]^+^ *m*/*z* values, which complemented the IR and NMR spectra for the confirmation of the expected structures of all the compounds. Additionally, the structure of the 7-(4-(dibutylamino)but-2ynyloxy) coumarin derivative **23** was confirmed by X-ray single-crystal diffraction studies (Figure 2).

### 2.2. Cytotoxicity Studies

All new 6- and 7-(4-aminobut-2-ynyloxy)-substituted coumarins **5**, **7**, **9**, **14**–**21**, **23**–**33**, **35**–**39**, **41**, **42**, **44**, and **45** were screened for their in vitro cytotoxicity and growth inhibitory activities against three different tumor types, namely the human cervical cancer (HPV-negative C33 A and HPV16-positive CaSki) and breast cancer (adenocarcinoma MCF-7 cell lines, in comparison with the activity of the known anticancer reference drug doxorubicin (DOX). Normal epithelial VERO cells were used as a non-cancer control. Cell viability was assessed by the 3-[4,5-dimethyl-thiazol-2-yl]-2,5-diphenyltetrazolium bromide (MTT) assay. The cytotoxic activities of the tested compounds were expressed as GI_50_ μM values (the dose that reduces growth inhibition to 50%) (Table 1). All tested coumarin derivatives were non-toxic for VERO cells (GI_50_ > 84 μM) relative to doxorubicin (GI_50_ = 8 ± 1.8 μM) (Table 1). The substituent at the nitrogen atom in the side chain of the butynyloxy moiety can enhance bioactivity, and the differential bioactivity exhibited by coumarin might be associated with its substitution position.

## 3. Discussion

Currently, we have studied the synthesis and cytotoxicity of compounds having a coumarin core and several nitrogen-substituted pharmacophores linked by the but-2-ynyloxy group at the C-6 or C-7 positions by the three-component one-pot copper-catalyzed Mannich reaction of 6-(prop-2-ynyloxy)-2*H*-chromen-2-one **1** and 7-(prop-2-ynyloxy)-2*H*-chromen-2-ones **2** and **3** with formaldehyde and secondary amines. As can be seen, the yields of the target compounds vary significantly (26–98%), which may be attributed to both the isolation conditions of the target products as precipitates and the basicity and electronic properties of the secondary amine; for example, in the case of 4-(tetrahydrofurancarbonyl)piperazine **13** or 1,2,3,4-tetrahydroisoquinoline **43**. Furthermore, the decrease in yield observed in reactions with (4-hydroxyphenyl)-substituted piperazine **34** may be due to the potential formation of phenoxyl radicals in the presence of copper(II) compounds [31]. In this work, we did not optimize the reaction conditions; however, if necessary, this could be performed for the most active compounds.

From the obtained results, the following points can still be concluded: The cytotoxicity profile indicates that the 6-(4-aminobutynyloxy)coumarins **5**, **7**, **9**, **14**–**17**, and 7-(4-aminobut-2-ynyloxy)coumarins **18**–**21**, **23**–**26**, **28**–**33**, **35**–**39**, **41**, **42**, **44**, and **45** demonstrated cytotoxic effect against cervical and breast cancer cell lines and compound with the substituent at the seventh position proved to be a little more effective, especially against breast carcinoma (MCF-7) cancer cell line. It has been observed that the cytotoxic effect depends on the nature of the substituent at the nitrogen atom of the but-2-ynyloxy fragment. SAR indicated that a heterocyclic substituent at C-5′ could significantly enhance the anticancer activity, as evident in the case of azepane-, azocane-, and piperazinyl-tethered coumarins. Hymechromone derivatives **26**, **29**, **31**, **33**, **36**, and **38** showed particularly substantially higher cytotoxicity and selectivity than the corresponding umbelliferone derivatives **25**, **28**, **30**, **32**, **35**, and **37.** In view of the activity and selectivity profile (Table 1), compounds **16**, **35**, **36**, and **38** exhibited selective cell growth inhibition activity on the HPV-negative human cancer cells, C33 A (GI_50_ = 5.2–7.1 μM, SI 14.1–16.3). Despite the lower cytotoxicity compared to a positive control, doxorubicin (GI_50_ = 2.5 μM), compounds **16**, **17**, **35**–**39**, and **42** inhibited growth of C33 A cells with greater selectivity (SI 8.5–16.3) than doxorubicin (SI 3.2). Notably, C33 A cells are negative for both DNA and RNA of human papillomavirus (HPV), distinguishing them from many other cervical cancer cell lines that often carry HPV integrations, in particular, CaSki [32]. This aspect makes the new compounds particularly valuable for studying cervical cancer that develops independently of HPV infection, including insights into alternative pathways of carcinogenesis.

Compound **29**, with an azocane substituent, and **37**, **38**, **39**, **41** and **42** with a piperazine substituent, showed selectivity for HPV-positive CaSki cell lines, with GI_50_ values comparable to or higher than doxorubicin (GI_50_ = 6.8–9.6 μM, SI 9.5–14.7). Among these derivatives, compounds **41** and **42** possessing a 4-piperonyl moiety at the piperazine ring exhibited selectivity towards CaSki cells.

It is known that the substitution patterns on the coumarin core significantly influence the pharmacological potential of this nucleus, especially with respect to breast cancer [33]. Most of the synthesized compounds possessed inhibitory effects against the MCF-7 cell line (GI_50_ ≤ 30 μM). Compounds **16**, **37**, and **38** with a 4-[(furan-2-carbonyl)piperazin-1-yl], or **17** and **39** with a 4-[(tetrahydro-furan-2-carbonyl)piperazin-1-yl] moiety at the C-6 or C-7 positions, as well as 7-(4-piperonyl)piperazine-substituted compounds **41** and **42**, were the most active ones (GI_50_ = 4.3–6.6 μM) with high selectivity (SI 15.0–19.8). The obtained data indicate the importance of the substituent at the fourth position of the (piperazin-1-yl)but-2-ynyloxy moiety for inhibition of the tumor cells’ growth; attachment at the C-6 or C-7 position to the coumarin core was toleratedfor retaining cytotoxicity.

Heterocyclic compounds having nitrogen, such as 1,2,3,4-tetrahydroisoquinolines, are of considerable interest since they constitute the core structural element of many alkaloids possessing enormous pharmacological activities [34,35]. The two synthesized tetrahydroisoquinoline-coumarin hybrids **44** and **45** displayed satisfactory activity against the tested cancer cell lines (GI_50_ = 12.5–19.4 μM).

Several coumarin derivatives were demonstrated to have enhanced affinity and selectivity for the G-quadruplex over the duplex structure [36,37,38,39]. Stabilization of G-quadruplexes of telomeric sequences by small molecules promotes inhibition of telomerase, which exhibits increased activity in tumor cells. Binding G-quadruplexes of oncogenic promoters leads to suppression of their expression. Targeting DNA secondary structures such as G-quadruplexes is now considered an appealing opportunity for drug intervention in anticancer therapy [40]. G-quadruplex (G4) is a well-known secondary nucleic acid structure containing guanine-rich sequences and plays a significant role in pharmacological and biological phenomena associated with oncological diseases. To date, a large number of DNA/RNA G4 ligands have been developed in several laboratories [41]. Recently, anthracene-9-propargylamine [42] and anthraquinone-1-propargylamine [43] scaffolds were characterized as new G-quadruplex ligands.

Based on these data, modeling of the interaction of new coumarin derivatives with G-quadruplex DNA motifs was performed to understand the possible binding mode of the synthesized coumarin derivatives bearing alkyne and aminoalkyl moieties to G-quadruplex DNA. The crystal structure of an intramolecular human telomeric DNA G-quadruplex in complex with the naphthalene diimide ligand MM41 (PDB ID: 3UYH, resolution 1.95 Å) [44] was used as the target. Docking calculations were carried out with the search space centered on the co-crystallized MM41 ligand.

**Figure 3:**
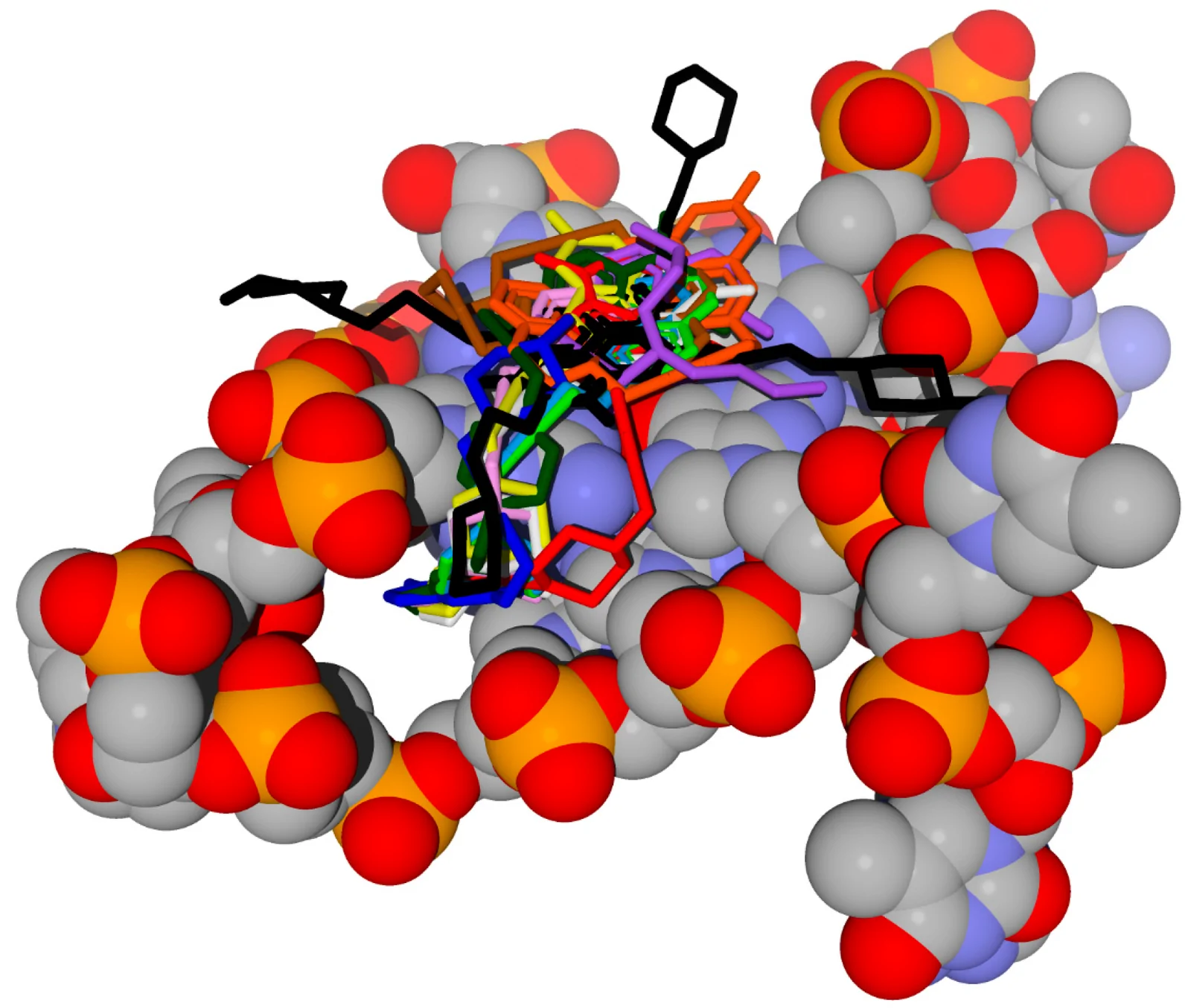
The superimposed docking poses of compounds **28** (brown), **25** (green), **14** (dark-blue), **9** (blue), **23** (cyan), **42** (dark-green), **36** (red), **45** (orange), **37** (yellow), **16** (magenta), and **38** (gray) in the binding site of the intramolecular human telomeric DNA G-quadruplex (PDB: 3UYH; balls). The co-crystallized naphthalene diimide ligand MM41 is shown in black sticks.

**Figure 4:**
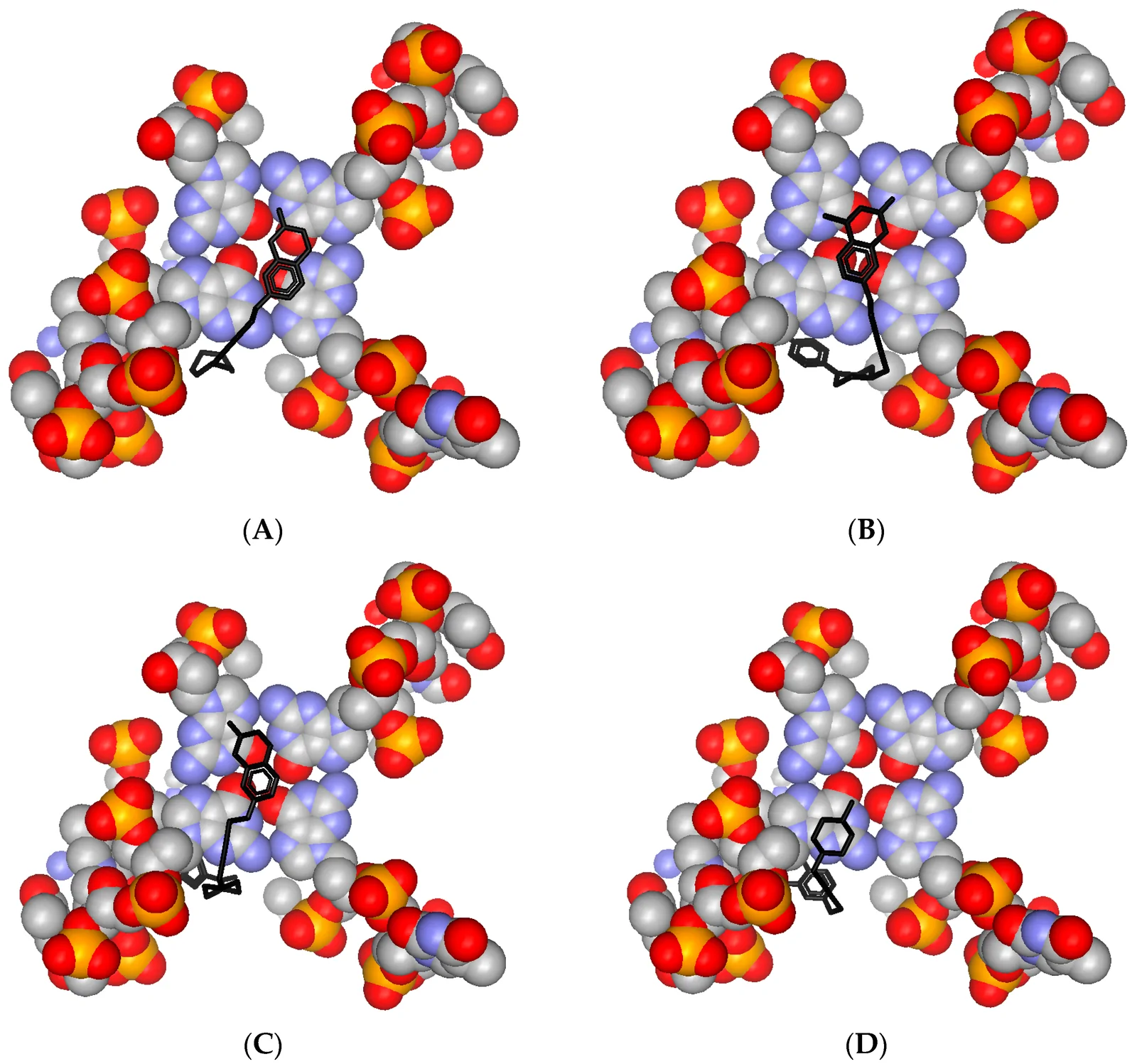
Top views of the docking poses of compounds **25** (**A**), **36** (**B**), **37** (**C**), and **14** (**D**) (black sticks). Guanine bases of the terminal G-tetrad with the adjacent DNA loop substructures are visible (balls). These panels provide a general overview of ligand positioning and loop penetration.

In most docked poses, the coumarin core of the investigated ligands was positioned over one or two guanine bases of the terminal G-tetrad, with the alkyne linker oriented approximately parallel to the plane of the quadruplex (Figure 3). Several poses displayed a distinct structural behavior. Specifically, certain derivatives demonstrated partial penetration of the terminal ligand moieties between the loop structures of the DNA sequence adjacent to the G-quadruplex, reaching towards the lower layer of bases. Thus, poses of the following compounds penetrated the loops via their respective terminal groups: **9** and **25** (azepan moiety), **36** (*p*-hydroxyphenylpiperazinyl moiety), and **42** (benzodioxolmethylpiperazine moiety), and the furan-2-carbonyl-piperazine derivatives **16**, **37**, and **38** (Figure 3 and Figure 4A–C).

Compound **14** exhibits a unique orientation where its N-methylated piperazine heterocycle is located on top of the quadruplex, while the coumarin moiety penetrates deeply between the loop structures (Figure 4D).

The docking scores obtained are presented in Table 2. The rerank scores ranged from −51.4 to −80.5, suggesting significant predicted binding to the G-quadruplex.

**Figure 5:**
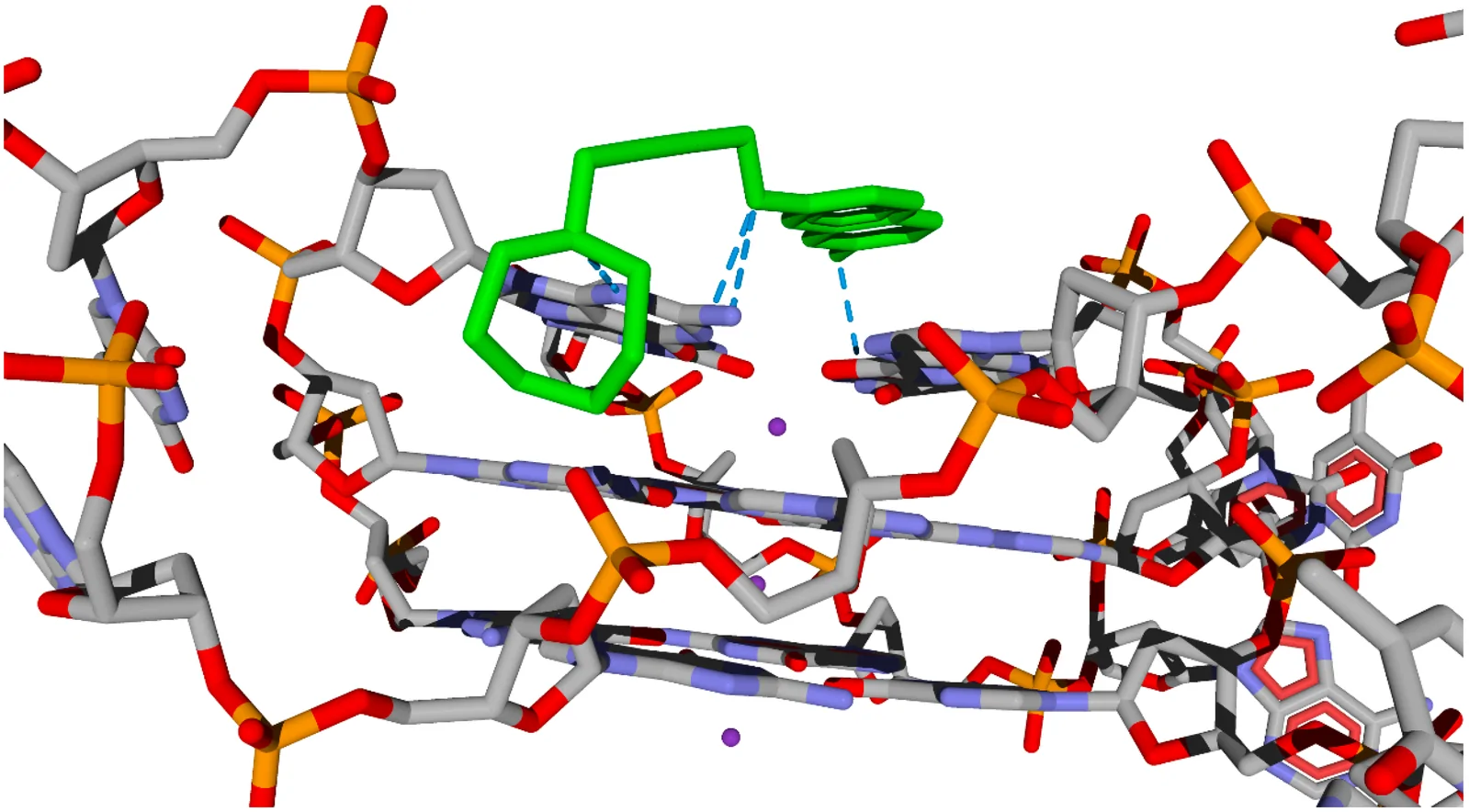
The docking pose of compound **25** (green). Hydrogen-bonding interactions with the G-quadruplex (PDB: 3UYH) are shown in blue dashed lines.

It should be noted that all the investigated compounds form numerous hydrogen bonds with the guanine bases (see example in Figure 5), which additionally anchor the ligands within the binding site.

The obtained docking results indicate that the combination of the coumarin scaffold, the rigid alkyne linker, and appropriately substituted tertiary amine moieties enables effective interaction with the G-quadruplex. Compounds showing deeper penetration into loop regions or more favorable rerank scores may possess enhanced stabilizing effects on the quadruplex structure, which could contribute to their potential anticancer activity through telomerase inhibition or oncogene promoter regulation. These computational results complement the experimental biological data and provide a structural basis for further optimization of coumarin-based G-quadruplex ligands. While the present in silico study offers an initial proof-of-concept for binding of these novel coumarin derivatives to the human telomeric G-quadruplex (PDB: 3UYH), we recognize that only one G4 conformation has been examined. Future work will extend comprehensive docking and molecular dynamics analyses to oncogene-promoter G-quadruplexes, including those of c-MYC and KRAS, with emphasis on the most active compounds reported here and on their selectively modified analogs.

## 4. Materials and Methods

### 4.1. General Information

Melting points were determined on the Mettler Toledo FP900 thermosystem (Columbus, OH, USA). NMR spectra were acquired on Bruker Avance-300 (^1^H: 300.13 MHz) and Bruker DRX 500 (^1^H: 500.13 MHz, ^13^C: 125.76 MHz) spectrometers (Bruker Corporation, Karlsruhe, Germany). Deuterochloroform (CDCl_3_) or ((CD_3_)_2_SO for compound **35** was used as a solvent. By using CDCl_3_ as a solvent, the residual CHCl_3_ (δ_H_ =7.24 ppm) and CDCl_3_ (δ_C_ = 76.8 ppm) were employed as internal standards, and by using (CD_3_)_2_SO as a solvent, the residual DMSO (δ_H_ = 2.51 ppm) and (CD_3_)_2_SO (δ_C_ = 39.51 ppm) were employed as the internal standards. NMR signal assignments were carried out with the aid of a combination of 1D and 2D NMR techniques that included ^1^H, ^13^C, COSY, HSQC, and HMBC spectra. In the description of the ^1^H and ^13^C-NMR spectra for all compounds, the atom numbering system given in Scheme 1 and Scheme 2 was used. IR absorption spectra were recorded on a Bruker Vector 22 FT-IR spectrometer (Bruker Corporation, Karlsruhe, Germany) in KBr pellets. UV spectra were obtained on an HP 8453 UV–Vis spectrometer (Hewlett-Packard, Waldbronn, Germany) in EtOH solution. Mass spectra were recorded on a DFS Thermo Scientific high-resolution mass spectrometer (evaporator temperature 120–280 °C, electron impact ionization at 70 eV) (Thermo Fisher Scientific, Waltham, MA, USA). The X-ray diffraction experiments for compound **23** were carried out at 296(2) K on a Bruker KAPPA APEX II CCD diffractometer (graphite-monochromated Mo Kα radiation). Reflection intensities were corrected for absorption by the multi-scan method with the SADABS2016/2 program [46]. The structure of compound **23** was solved by direct methods using SHELXT-2014/5 [47] and refined by the anisotropic (isotropic for all H atoms) full-matrix least-squares method against *F*^2^ of all reflections by SHELXL2018/3 [46]. Crystallographic data for the structure have been deposited at the Cambridge Crystallographic Data Centre as supplementary publication no. CCDC 2498361. These data can be obtained free of charge from The Cambridge Crystallographic Data Centre via www.ccdc.cam.ac.uk/data_request/cif (accessed on 29 October 2025).

The reaction progress was controlled by TLC on Sorbfil UV-254 plates using CHCl_3_-EtOH, 10:1, or petroleum ether-EtOAc, 3:1; detection was under UV light or by treatment with iodine vapor. Column chromatography was performed using silica gel 60 (0.063–0.200 mm, Merck KGaA, Darmstadt, Germany).

The chemicals used were as follows: 6-hydroxycoumarin, umbelliferone, hymechromone (7-hydroxy-4-methylcoumarin), diisopropylamine **4**, dipropylamine **6**, dibutylamine **22**, azepane **8**, azocane **27**, N-substituted piperazines **10**–**13**, **34**, **40**, aq. formaldehyde (30% in aq. solution), Cu(OAc)_2_·H_2_O, and 1,2,3,4-tetrahydroisoquinoline **43**; these were purchased from Alfa Aesar and Macklin. 6-(Prop-2-ynyloxy)-2*H*-chromen-2-one **1** [27], 7-prop-2-ynyloxy)-2*H*-chromen-2-one **2** [27,28], and 4-methyl-7-prop-2-ynyloxy)- 2*H*-chromen-2-one **3** [29] were prepared following literature procedures. The solvents (1,4-dioxane, CHCl_3_, EtOH, and EtOAc) were purified according to standard methods and distilled immediately before use.

### 4.2. General Procedure for Synthesis of N-Substituted 6-(4-Aminobut-2-Ynyloxy)-Chromen-2-Ones (**5**, **7**, **9**, **14**–**17**), 7-(4-Aminobut-2-Ynyloxy)Chromen-2-Ones (**18**, **20**, **23**, **25**, **28**, **30**, **32**, **35**, **37**, **39**, **41**, **44**), and 7-(4-Aminobut-2-Ynyloxy)-4-Methylchromen-2-Ones (**19**, **21**, **24**, **26**, **29**, **31**, **33**, **36**, **38**, **42**, **45**)

Secondary amines **4**, **6**, **8**, **10**–**13**, **22**, **27**, **34**, **40**, or **43** (8.4–10 mmol), aq. formaldehyde (60 mg, 20 mmol), and Cu(OAc)_2_·H_2_O (0.47–0.5 mmol) were successively added in argon to the solution of prop-2-ynyloxy-substituted coumarine **1**, **2**, or **3** (4.7–5 mmol) in dioxane (25 mL), and the reaction mixture was stirred at 65–70 °C for a period of 3 to 5 h (TLC-control). After the consumption of the starting materials, the reaction mixture was cooled and evaporated in vacuo. The residue was dissolved in EtOAc (150–200 mL), washed with brine, dried over MgSO_4_, filtered, and evaporated. The oily residue was dissolved in a minimum amount of CHCl_3_ and separated by silica gel column chromatography (eluent CHCl_3_–EtOH, gradient 100:0.5 → 10:1).

**6-(4-(Diisopropylamino)but-2-ynyloxy)-2*H*-chromen-2-one (5).** White solid; yield 77% (0.132 g); mp 86–88 °C; IR (KBr) ν: 2245 (C≡C), 1699 (C=O), 1627, 1568 (C=C), 1270, 1176, 1106 (C–O–C) cm^−1^; UV (EtOH) λ_max_ (lgε): 277 (4.08), 337 (3.66), nm; ^1^H NMR (CDCl_3_, 300 MHz) δ: 7.61 (1H, d, *J* = 9.6 Hz, H-4), 7.24 (1H, d, *J* = 9.0 Hz, H-8), 7.14 (1H, dd, *J* = 9.0, 2.9 Hz, H-7), 7.00 (1H, d, *J* = 2.9 Hz, H-5), 6.40 (1H, d, *J* = 9.6 Hz, H-3), 4.70 (2H, br. s, H-2′), 3.40 (2H, br. s, H-5′), 3.08 (2H, m, 2-CH-*i*-*Pr*), 1.00 (12H, d, *J* = 6.5 Hz, 4×CH_3_-*i*-*Pr*) ppm; ^13^C NMR (CDCl_3_, 125 MHz) δ: 160.56 (C-2), 153.71 (C-6), 148.41 (C-8a), 142.78 (C-4), 120.06 (C-8), 118.68 (C-4a), 117.45 (C-7), 116.74 (C-3), 111.36 (C-5), 87.37 (C-4′), 76.92 (C-3′), 56.59 (C-2′), 48.07 (C-5′), 33.91 (2-CH-*i*-*Pr*), 20.01 (4-CH_3_-*i*-*Pr*) ppm; HR-MS, *m*/*z* (I*_rel_.*, %): 314 (3), 313 (10.6, M^+^), 299 (20), 298 (100), 122 (10), 70 (13), 43 (12); calcd for C_19_H_23_NO_3_: 313.1673; found *m*/*z* 313.1670 [M]^+^.

**6-(4-(Dipropylamino)but-2-ynyloxy)-2*H*-chromen-2-one (7).** White solid; yield 56% (0.390 g); mp 62–65 °C; IR (KBr) ν: 2248 (C≡C), 1709 (C=O), 1614, 1506 (C=C), 1277, 1126, 1080 (C–O–C) cm^−1^; ^1^H NMR (CDCl_3_, 300 MHz) δ: 7.62 (1H, d, *J* = 9.6 Hz, H-4), 7.25 (1H, d, *J* = 8.9 Hz, H-8), 7.16 (1H, dd, *J* = 8.9, 2.9 Hz, H-7), 7.02 (1H, d, *J* = 2.9 Hz, H-5), 6.40 (1H, d, *J* = 9.6 Hz, H-3), 4.74 (2H, br. s., H-2′), 3.38 (2H, br. s, H-5′), 2.32 (4H, t, *J* = 7.5 Hz, 2CH_2_-*Pr*), 1.39 (4H, m, 2-CH_2_*-Pr*), 0.81 (6H, t, *J* = 7.4 Hz, 2-CH_3_-*Pr*) ppm; ^13^C NMR (CDCl_3_, 125 MHz) δ: 160.78 (C-2), 153.85 (C-6), 148.70 (C-8a), 143.01 (C-4), 120.21 (C-8), 118.93 (C-4a), 117.73 (C-3), 117.04 (C-7), 111.76 (C-5), 83.64 (C-4′), 78.65 (C-3′), 56.67 (C-2′), 55.63 (C-5′), 41.94 (2-CH_2_*-Pr*), 20.44 (2-CH_2_*-Pr*), 11.73 (2-CH_3_*-Pr*) ppm; HR-MS, *m*/*z* (I*_rel_.*, %): 314 (0.4), 313 (2.4, M^+^), 285 (19), 284 (100), 133 (23), 43 (13); calcd for C_19_H_23_NO_3_: 313.1673; found *m*/*z* 313.1677 [M]^+^.

**6-(4-(Azepan-1-yl)but-2-ynyloxy)-2*H*-chromen-2-one** (**9**). White solid. Yield 33% (0.260 g); mp 79–82 °C; IR (KBr) ν: 2255 (C≡C), 1709 (C=O), 1608, 1567 (C=C), 1273, 1111, 1087 (C–O–C) cm^−1^; UV (EtOH) λ_max_ (lgε): 277 (4.07), 338 (3.67) nm; ^1^H NMR (CDCl_3_, 300 MHz) δ: 7.63 (1H, d, *J* = 9.6 Hz, H-4), 7.25 (1H, d, *J* = 8.8 Hz, H-8), 7.15 (1H, dd, *J* = 8.8, 2.9 Hz, H-7), 7.01 (1H, d, *J* = 2.9 Hz, H-5), 6.40 (1H, d, *J* 9.6 Hz, H-3), 4.73 (2H, br. s, H-2′), 3.35 (2H, br. s, H-5′), 2.57 (4H, dd, *J* = 11.2, 5.5 Hz, H-7′, 12′), 1.59–1.49 (8H, m, H-8′, 9′, 10′, 11′) ppm; ^13^C NMR (CDCl_3_, 125 MHz) δ: 160.74 (C-2), 153.88 (C-6), 148.68 (C-8a), 142.96 (C-4), 120.22 (C-8), 118.95 (C-4a), 117.71 (C-7), 116.99 (C-3), 111.68 (C-5), 84.80 (C-4′), 78.12 (C-3′), 56.76 (C-2′), 55.20 (C-7′, 12′), 48.09 (C-5′), 28.08 (C-8′, 11′), 26.41 (C-9′, 10′) ppm; HR-MS, *m*/*z* (I*_rel_.*, %): 312 (11), 311 (42, M^+^), 310 (23), 151 (11), 150 (100), 149 (36), 148 (19), 94 (11); calcd for C_19_H_21_NO_3_: 311.1516; found *m*/*z* 311.1513 [M]^+^.

**6-(4-(4-Methylpiperazin-1-yl)but-2-ynyloxy)-2*H*-chromen-2-one** (**14**). White solid; yield 44% (0.194 g); mp 112–114 °C; IR (KBr) ν: 2250 (C≡C), 1707 (C=O), 1609, 1570, 1491 (C=C), 1340, 1138, 1011, 921 (piperazine), 1275, 1230, 1077 (C–O–C) cm^−1^; UV (EtOH) λ_max_ (lgε): 277 (4.06), 338 (3.66) nm; ^1^H NMR (CDCl_3_, 300 MHz) δ: 7.65 (1H, d, *J* = 9.6 Hz, H-4), 7.24 (1H, d, *J* = 8.6 Hz, H-8), 7.13 (1H, dd, *J* = 8.6, 2.7 Hz, H-7), 7.00 (1H, d, *J* = 2.7 Hz, H-5), 6.40 (1H, d, *J* = 9.6 Hz, H-3), 4.71 (2H, br. s, H-2′), 3.30 (2H, br. s, H-5′), 2.74–2.30 (8H, m, H-7′, 8′, 10′, 11′), 2.25 (3H, s, NCH_3_) ppm; ^13^C NMR (CDCl_3_, 125 MHz) *δ*: 160.76 (C-2), 153.88 (C-6), 148.76 (C-8a), 143.05 (C-4), 120.20 (C-8), 119.02 (C-4a), 117.77 (C-7), 117.01 (C-3), 111.68 (C-5), 83.26 (C-4′), 79.32 (C-3′), 56.70 (C-2′), 54.79 (C-8′, 10′), 51.79 (C-7′, 11′), 46.89 (C-5′), 45.86 (NCH_3_) ppm; HR-MS, *m*/*z* (I*_rel_.*, %): 313 (2), 312 (6, M^+^), 311 (0.6), 274 (3), 162 (10), 163 (11), 152 (10), 151 (100), 150 (35), 108 (43), 99 (41), 85 (21), 83 (27), 70 (29), 58 (25), 43 (26), 42 (29); calcd. for C_18_H_20_N_2_O_3_: 312.1468; found *m*/*z* 312.1465 [M]^+^.

**6-(4-(4-Phenylpiperazin-1-yl)but-2-ynyloxy)-2*H*-chromen-2-one** (**15**). White solid; yield 39% (0.220 g); mp 118–120 °C; IR (KBr) ν: 2285 (C≡C), 1707 (C=O), 1629, 1597, 1564, 1504, 811, 754, 688 (C=C, CH-Ar), 1340, 1138, 1011, 941 (piperazine), 1277, 1232, 1070 (C–O –C); UV (EtOH) λ_max_ (lgε): 277 (4.10), 338 (3.66) nm; ^1^H NMR (CDCl_3_, 300 MHz) δ: 7.62 (1H, d, *J* = 9.6 Hz, H-4), 7.28–7.22 (3H, m, H-8, 3″, 5″), 7.15 (1H, dd, *J* = 8.6, 2.9 Hz, H-7), 7.03 (1H, d, *J* = 2.9 Hz, H-5), 6.89 (2H, d, *J* = 7.9 Hz, H-2″, 6″), 6.85 (1H, t, *J* = 7.9 Hz, H-4″), 6.31 (1H, d, *J* = 9.6 Hz, H-3), 4.75 (2H, br. s, H-2′), 3.39 (2H, br. s., H-5′), 3.18 (4H, m, H-8′, 10′), 2.67 (4H, m, H-7′, 11′) ppm; ^13^C NMR (CDCl_3_, 125 MHz) δ: 160.69 (C-2), 153.83 (C-6), 150.93 (C-1″), 148.76 (C-8a), 142.97 (C-4), 129.03 (C-3″, 5″), 120.24 (C-8), 119.82 (C-4″), 118.99 (C-4a), 117.79 (C-7), 117.02 (C-3), 115.99 (C-2″, 6″), 111.65 (C-5), 83.08 (C-4′), 79.63 (C-3′), 56.66 (C-2′), 51.79 (C-7′, 11′), 48.99 (C-8′, 10′), 46.98 (C-5′) ppm; HR-MS, *m*/*z* (I*_rel_.*, %): 375 (6), 374 (21, M^+^), 373 (2), 214 (15), 213 (96), 212 (27), 211 (52), 198 (15), 159 (20), 133 (24), 132 (34), 120 (40); 118 (28), 108 (45), 105 (33), 104 (100), 77 (31), 56 (26); calcd for C_23_H_22_N_2_O_3_: 374.1625; found *m*/*z* 374.1621 [M]^+^.

**6-(4-(4-(Furan-2-carbonyl)piperazin-1-yl)but-2-ynyloxy)-2*H*-chromen-2-one** (**16**). White solid; yield 38% (0.217 g); mp 107–109 °C; IR (KBr) ν: 2280 (C≡C), 1707 (C=O), 1656 (C=O), 1616, 1567, 1491, 811, 748, 706 (C=C, CH-Ar), 1302, 1138, 1005, 908 (piperazine), 1275, 1176, 1056 (C–O–C) cm^−1^; UV (EtOH) λ_max_ (lgε): 264 (3.58), 338 (4.18) nm; ^1^H NMR (CDCl_3_, 300 MHz) δ: 7.61 (1H, d, *J* = 9.5 Hz, 1H, H-4), 7.46 (1H, d, *J* = 1.6 Hz, H-5″), 7.24 (1H, d, *J* = 8.8 Hz, H-8), 7.13 (1H, dd, *J* = 8.8, 2.8 Hz, H-7), 7.01 (1H, d, *J* = 2.8 Hz, H-5), 6.98 (1H, d, *J* = 3.5 Hz, H-3″), 6.46 (1H, dd, *J* = 3.6, 1.6 Hz, H-4″), 6.38 (1H, d, *J* = 9.5 Hz, H-3), 4.73 (2H, br. s, 2H, H-2′), 3.80 (4H, m, H-8′, 10′), 3.38 (2H, br. s, H-5′), 2.55 (4H, m, H-7′, 11′) ppm; ^13^C NMR (CDCl_3_, 125 MHz) δ: 160.74 (C-2), 158.90 (C-6), 153.81 (C=O), 148.76 (C-8a), 147.69 (C-2″), 143.69 (C-5″), 142.93 (C-4), 120.09 (C-8), 119.03 (C-4a), 117.83 (C-7), 117.21 (C-3), 116.60 (C-3″), 111.57 (C-5), 111.29 (C-4″), 82.47 (C-4′), 79.92 (C-3′), 56.58 (C-2′), 51.81 (C-7′, 11′), 46.92 (C-5′), 42.37 (C-8′, 10′) ppm; HR-MS, *m*/*z* (I*_rel_.*, %): 393 (2), 392 (5, M^+^), 286 (5), 268 (11), 231 (17), 230 (32), 200 (14), 162 (29), 161 (38), 138 (28), 134 (25), 133 (16), 119 (31), 106 (16), 105 (13), 97 (14), 95 (100), 85 (10), 77 (15), 56 (19). 55 (20); calcd for C_22_H_20_N_2_O_5_: 392.1367; found *m*/*z* 392.1368 [M]^+^.

**6-(4-(4-(Tetrahydrofuran-2-carbonyl)piperazin-1-yl)but-2-ynyloxy)-2*H*-chromen-2-one (17)**. White solid; yield 37% (0.213 g); mp 102–105 °C; IR (KBr) ν: 2246 (C≡C), 1708 (C=O), 1648 (C=O), 1600, 1570, 1500, 813, 706 (C=C, CH-Ar), 1338, 1112, 1005, 910 (piperazine), 1266, 1165, 1045 (C–O–C) cm^−1^; UV (EtOH) λ_max_ (lgε): 277 (4.02), 338 (3.60) nm; ^1^H NMR (CDCl_3_, 300 MHz) δ: 7.64 (1H, d, *J* = 9.6 Hz, H-4), 7.25 (1H, d, *J* = 9.0 Hz, H-8), 7.13 (1H, dd, *J* = 9.0, 2.8 Hz, H-7), 6.98 (1H, d, *J* = 2.8 Hz, H-5), 6.42 (1H, d, *J* = 9.6 Hz, H-3), 4.73 (2H, br. s, H-2′), 4.55 (1H, dd, *J* = 8.0, 5.6 Hz, H-2″), 3.91 (1H, m, H-5″), 3.81 (1H, m, H-5″), 3.73–3.60 (2H, m, H-8′, 10′), 3.57–3.47 (2H, m, H-8′, 10′), 3.34 (2H, br. s, H-5′), 2.54–2.43 (4H, m, H-7′, 11′), 2.27–2.19 (1H, m, H-3″), 2.05–1.83 (3H, m, H-3″, 4″, 4″) ppm; ^13^C NMR (125 MHz, CDCl_3_) *δ* 169.64 (C=O), 160.77 (C-2), 153.81 (C-6), 148.71 (C-8a), 142.98 (C-4), 120.01 (C-8), 118.99 (C-4a), 117.79 (C-7), 117.12 (C-3), 111.50 (C-5), 82.55 (C-4′), 79.68 (C-3′), 75.67, 68.97 (C-2″), 56.54 (C-2′), 51.93 (C-8′, 10′), 51.48 (C-7′, 11′), 46.94 (C-5′), 45.00, 41.59 (C-5″), 28.28 (C-3″), 25.61 (C-4″) ppm; HR-MS, *m*/*z* (I*_rel_.*, %): 398 (6), 397 (25), 396 (100, M^+^), 395 (3), 297 (12), 285 (11), 235 (43), 234 (79), 162 (57), 142 (23), 134 (30), 119 (24), 106 (18), 71 (62); calcd for C_22_H_24_N_2_O_5_: 396.1680; found *m*/*z* 396.1675 [M]^+^.

**7-(4-(Diisopropylamino)but-2-ynyloxy)-2*H*-chromen-2-one** (**18**). White solid; yield 98% (1.56 g); mp 72–75 °C; IR (KBr) ν: 2219 (C≡C), 1720 (C=O), 1616, 1504 (C=C), 1277, 1163, 1086 (C–O–C) cm^−1^; ^1^H NMR (CDCl_3_,300 MHz) δ: 7.62 (1H, d, *J* = 9.5 Hz, H-4), 7.35 (1H, d, *J* = 8.5 Hz, H-5), 6.90 (1H, d, *J* = 2.4 Hz, H-8), 6.87 (1H, dd, *J* = 8.5, 2.4 Hz, H-6), 6.25 (1H, d, *J* = 9.5 Hz, H-3), 4.73 (2H, br. s, H-2′), 3.41 (2H, br s, H-5′), 3.10 (2H, m, 2-CH-*i*-*Pr*), 1.01 (12H, d, *J* = 6.5 Hz, 4×CH_3_-*i*-*Pr*) ppm; ^13^C NMR (CDCl_3_, 125 MHz) δ: 161.03 (C-2), 160.71 (C-7), 155.44 (C-8a), 143.28 (C-4), 128.54 (C-5), 113.29 (C-3), 113.11 (C-6), 112.76 (C-4a), 102.08 (C-8), 87.81 (C-4′), 77.12 (C-3′), 56.69 (C-2′), 48.25 (C-5′), 34.12 (2-CH-*i*-Pr), 20.03 (4-CH_3_-*i*-*Pr*) ppm; HR-MS, *m*/*z* (I*_rel_.*, %): 314 (2), 313 (8, M^+^), 299 (22), 298 (100), 256 (9), 213 (7), 137 (7), 95 (7), 70 (9); calcd for C_19_H_23_NO_3_: 313.1673; found *m*/*z* 313.1671 [M]^+^.

**7-(4-(Diisopropylamino)but-2-ynyloxy)-4-methyl-2*H*-chromen-2-one** (**19**). White solid; yield 79% (1.20 g); mp 122–124 °C; IR (KBr) ν: 2230 (C≡C), 1728 (C=O), 1608, 1510 (C=C), 1277, 1167, 1093 (C–O–C) cm^−1^; UV (EtOH) λ_max_ (lgε): 275 (3.89), 320 (4.19) nm; ^1^H NMR (CDCl_3_,300 MHz) δ: 7.47 (1H, d, *J* = 8.5 Hz, H-5), 6.90 (1H, d, *J* = 2.4 Hz, H-8), 6.88 (1H, dd, *J* = 8.5, 2.4 Hz, H-6), 6.12 (1H, br. s, H-3), 4.73 (2H, br. s, H-2′), 3.41 (2H, br. s, H-5′), 3.10 (2H, m, 2-CH-*i*-*Pr*), 2.37 (3H, br. s., 4-CH_3_), 1.02 (12H, d, *J* = 6.5 Hz, 4×CH_3_-*i*-*Pr*) ppm; ^13^C NMR (CDCl_3_, 125 MHz) δ: 161.07 (C-2), 160.54 (C-7), 154.92 (C-8a), 152.32 (C-4), 126.22 (C-5), 113.88 (C-4a), 112.81 (C-3), 112.14 (C-6), 102.15 (C-8), 88.04 (C-4′), 76.72 (C-3′), 56.69 (C-2′), 48.36 (C-5′), 34.15 (2-CH-*i*-*Pr*), 20.41 (4-CH_3_-*i*-*Pr*), 18.56 (CH_3_) ppm; HR-MS, *m*/*z* (I*_rel_.*, %): 328 (2), 327 (7, M^+^), 313 (23), 312 (100), 298 (8), 270 (7), 227 (8), 70 (9); calcd for C_20_H_25_NO_3_: 327.1829; found *m*/*z* 327.1830 [M]^+^.

**7-(4-(Dipropylamino)but-2-ynyloxy)-2*H*-chromen-2-one (20).** White solid; yield 63% (0.492 g); mp 66–68 °C; IR (KBr) ν: 2235 (C≡C), 1709 (C=O), 1614, 1506 (C=C), 1273, 1156, 1093 (C–O–C) cm^−1^; UV (EtOH) λ_max_ (lgε): 277 (4.06), 330 (3.66) nm; ^1^H NMR (CDCl_3_, 300 MHz) δ: 7.61 (1H, d, *J* = 9.6 Hz, H-4), 7.36 (1H, d, *J* = 8.6 Hz, H-5), 6.92 (1H, d, *J* = 2.3 Hz, H-8), 6.88 (1H, dd, *J* = 8.6, 2.3 Hz, H-6), 6.24 (1H, d, *J* = 9.6 Hz, H-3), 4.76 (2H, br. s, H-2′), 3.40 (2H, br. s, H-5′), 2.33 (4H, t, *J* = 7.3 Hz, 2CH_2_-*Pr*), 1.40 (4H, m, 2-CH_2_-*Pr*), 0.82 (6H, t, *J* = 7.3 Hz, 2-CH_3_-*Pr*) ppm; ^13^C NMR (CDCl_3_, 125 MHz) δ: 160.78 (C-2), 160.53 (C-7), 155.41 (C-8a), 143.01 (C-4), 128.45 (C-5), 113.24 (C-6), 112.99 (C-3), 112.70 (C-4a), 102.02 (C-8), 83.99 (C-4′), 77.98 (C-3′), 56.43 (C-2′), 55.48 (C-5′), 41.85 (2-CH_2_-*Pr*), 20.44 (2-CH_2_-*Pr*), 11.55 (2-CH_3_-*Pr*) ppm; HR-MS, *m*/*z* (I*_rel_.*, %): 314 (0.4), 313 (2.4, M^+^), 285 (19), 284 (100), 133 (23), 43 (13); calcd for C_19_H_23_NO_3_: 313.1673; found *m*/*z* 313.1677 [M]^+^.

**7-(4-(Dipropylamino)but-2-ynyloxy)-4-methyl-2*H*-chromen-2-one (21).** White solid; yield 74% (0.570 g); mp 106–109 °C; IR (KBr) ν: 2245 (C≡C), 1717 (C=O), 1610, 1510 (C=C), 1267, 1203, 1155, 1088 (C–O–C) cm^−1^; ^1^H NMR (CDCl_3_, 300 MHz) δ: 7.48 (1H, d, *J* = 8.6 Hz, H-5), 6.92 (1H, d, *J* = 2.5 Hz, H-8), 6.89 (1H, dd, *J* = 8.6, 2.5 Hz, H-6), 6.12 (1H, s, H-3), 4.76 (2H, br. s, H-2′), 3.40 (2H, br. s, H-5′), 2.37 (s, 3H, CH_3_), 2.33 (4H, t, *J* = 7.5 Hz, 2CH_2_-*Pr*), 1.39 (4H, m, 2-CH_2_-*Pr*), 0.81 (6H, t, *J* = 7.5 Hz, 2-CH_3_-*Pr*) ppm; ^13^C NMR (CDCl_3_, 125 MHz) δ: 161.17 (C-2), 160.42 (C-7), 154.87 (C-8a), 152.43 (C-4), 125.41 (C-5), 113.89 (C-4a), 112.85 (C-3), 112.16 (C-6), 102.03 (C-8), 83.91 (C-4′), 78.18 (C-3′), 56.44 (C-2′), 55.61 (C-5′), 41.92 (2-CH_2_-*Pr*), 20.51 (2-CH_2_-*Pr*), 18.56 (4-CH_3_), 11.75 (2-CH_3_-*Pr)* ppm; HR-MS, *m*/*z* (I*_rel_.*, %): 328 (0.8), 327 (4, M^+^), 299 (22), 298 (100), 123 (11); calcd for C_20_H_25_NO_3_: 327.1829; found *m*/*z* 327.1828 [M]^+^.

**7-(4-(Dibutylamino)but-2-ynyloxy)-2*H*-chromen-2-one (23).** White solid; yield 68% (0.570 g); mp 68–70 °C; IR (KBr) ν: 2250 (C≡C), 1708 (C=O), 1614, 1506 (C=C), 1232, 1128, 1080 (C–O–C) cm^−1^; ^1^H NMR (CDCl_3_, 300 MHz) δ: 7.61 (1H, d, *J* = 9.6 Hz, H-4), 7.35 (1H, d, *J* = 8.5 Hz, H-5), 6.91 (1H, d, *J* = 2.0 Hz, H-8), 6.88 (1H, dd, *J* = 8.5, 2.0 Hz, H-6), 6.24 (1H, d, *J* = 9.6 Hz, H-3), 4.76 (2H, br. s, H-2′), 3.39 (2H, br. s, H-5′), 2.36 (4H, t, *J* = 7.0 Hz, 2CH_2_-*Bu*), 1.40–1.17 (8H, m, 4-CH_2_-*Bu*), 0.84 (6H, t, *J* = 7.0 Hz, 2-CH_3_-*Bu*) ppm; ^13^C NMR (CDCl_3_, 125 MHz) δ: 160.97 (C-2), 160.59 (C-7), 155.50 (C-8a), 143.19 (C-4), 128.56 (C-5), 113.29 (C-3), 113.08 (C-6), 112.82 (C-4a), 102.17 (C-8), 83.88 (C-4′), 78.24 (C-3′), 56.50 (C-2′), 53.48 (C-5′), 41.93 (2-CH_2_-*Bu*), 20.49 (4-CH_2_-*Bu*), 13.88 (2-CH_3_-*Bu*) ppm; HR-MS, *m*/*z* (I*_rel_.*, %): 341 (4, M^+^), 299 (19), 298 (100), 134 (14), 133 (11), 57 (9); calcd for C_21_H_27_NO_3_: 341.1986; found *m*/*z* 341.1989 [M]^+^. Crystal data **23**: C_21_H_27_N_1_O_3_, M = 341.43, triclinic, space group *P*-*1*, *a* = 6.7350 (5), *b* = 10.0456 (7), *c* = 16.1032 (10) Å, α = 104.208 (3), β = 93.498 (2), γ = 107.735 (2)°, *V* = 995.04(12) Å^3^, *Z* = 2, d_calc_ = 1.140 g·cm^−3^, µ = 0.075 mm^−1^, a total of 23,534 (θ_max_ = 27.56°), 4337 unique (*R*_int_ = 0.0401), 2865 [*I* > 2σ(*I*)], 228 parameters. GooF = 1.024, *R*_1_ = 0.0772, *wR*_2_ = 0.2333 [*I* > 2σ(*I*)], *R*_1_ = 0.1076, *wR*_2_ = 0.2724 (all data), max/min diff. peak 0.447/-0.395 e·Å^−3^.

**7-(4-(Dibutylamino)but-2-ynyloxy)-4-methyl-2*H*-chromen-2-one (24).** White solid; yield 93% (0.775 g); mp 85–87 °C; IR (KBr) ν: 2245 (C≡C), 1728 (C=O), 1610, 1510 (C=C), 1255, 1130, 1085 (C–O–C) cm^−1^; ^1^H NMR (CDCl_3_, 300 MHz) δ: 7.52 (1H, d, *J* = 8.5 Hz, H-5), 6.96 (1H, d, *J* = 2.0 Hz, H-8), 6.93 (1H, dd, *J* = 8.5, 2.0 Hz, H-6), 6.17 (1H, br. s, H-3), 4.81 (2H, br. s, H-2′), 3.44 (2H, br. s, H-5′), 2.40–2.38 (4H, t, *J* = 7.2 Hz, 2CH_2_-*Bu*), 2.41 (3H, br. s, CH_3_), 1.39 (4H, m, 2CH_2_-*Bu*), 1.28 (4H, m, 2-CH_2_-*Bu*), 0.88 (6H, t, *J* = 7.2 Hz, 2-CH_3_-*Bu*) ppm; ^13^C NMR (CDCl_3_, 125 MHz) δ: 161.07 (C-2), 160.39 (C-7), 154.68 (C-8a), 152.34 (C-4), 125.32 (C-5), 112.89 (C-4a), 112.74 (C-3), 112.09 (C-6), 102.88 (C-8), 83.99 (C-4′), 78.21 (C-3′), 56.46 (C-2′), 55.53 (2-CH_2_-*Bu*), 41.92 (C-5′), 29.46 (2-CH_2_-*Bu*), 20.50 (2-CH_2_-*Bu*), 18.56 (4-CH_3_), 13.69 (2-CH_3_-*Bu*) ppm; HR-MS, *m*/*z* (I*_rel_.*, %): 356 (1), 355 (4, M^+^), 313 (23), 312 (100), 274 (10), 137 (13), 98 (12), 86 (17); calcd for C_22_H_29_NO_3_: 355.2142; found *m*/*z* 355.2145 [M]^+^.

**7-(4-(Azepan-1-yl)but-2-ynyloxy)-2*H*-chromen-2-one** (**25**). White solid. Yield 55% (0.860 g); mp 75–78 °C; IR (KBr) ν: 2260 (C≡C), 1706 (C=O), 1614, 1506 (C=C), 1237, 1126, 1080 (C–O–C) cm^−1^; ^1^H NMR (CDCl_3_, 300 MHz) δ: 7.65 (1H, d, *J* = 9.6 Hz, H-4), 7.39 (1H, d, *J* = 8.6 Hz, H-5), 6.94 (1H, d, *J* = 2.3 Hz, H-8), 6.92 (1H, dd, *J* = 8.6, 2.3 Hz, H-6), 6.28 (1H, d, *J* 9.6 Hz, H-3), 4.78 (2H, br. s, H-2′), 3.37 (2H, br. s, H-5′), 2.58–2.52 (4H, m, H-7′, 12′), 1.61–1.53 (8H, m, H-8′, 9′, 10′, 11′) ppm; ^13^C NMR (CDCl_3_, 125 MHz) δ: 160.86 (C-2), 160.66 (C-7), 155.57 (C-8a), 143.17 (C-4), 128.58 (C-5), 113.36 (C-6), 113.11 (C-3), 112.86 (C-4a), 102.18 (C-8), 86.25 (C-4′), 77.55 (C-3′), 56.58 (C-2′), 55.44 (C-7′, 12′), 48.21 (C-5′), 28.01 (C-8′, 11′), 20.52 (C-9′, 10′) ppm; HR-MS, *m*/*z* (I*_rel_.*, %): 314 (0.8), 313 (3.6, M^+^), 285 (16), 284 (100), 123 (10); calcd for C_19_H_23_NO_3_: 313.1673; found *m*/*z* 313.1678 [M]^+^.

**7-(4-(Azepan-1-yl)but-2-ynyloxy)-4-methyl-2*H*-chromen-2-one** (**26**). White solid. Yield 71% (1.16 g); mp 108–110 °C; IR (KBr) ν: 2254 (C≡C), 1727 (C=O), 1610, 1510 (C=C), 1267, 1203, 1070 (C–O–C) cm^−1^; ^1^H NMR (CDCl_3_, 300 MHz) δ: 7.48 (1H, d, *J* = 8.5 Hz, H-5), 6.92 (1H, d, *J* = 2.2 Hz, H-8), 6.90 (1H, dd, *J* = 8.5, 2.2 Hz, H-6), 6.12 (1H, s, H-3), 4.72 (2H, br. s, H-2′), 3.37 (2H, br. s, H-5′), 2.61–2.57 (4H, m, H-7′, 12′), 2.37 (3H, s, CH_3_), 1.64–1.50 (8H, m, H-8′, 9′, 10′, 11′) ppm; ^13^C NMR (CDCl_3_, 125 MHz) δ: 161.04 (C-2), 160.37 (C-7), 154.82 (C-8a), 152.33 (C-4), 125.36 (C-5), 113.89 (C-4a), 112.75 (C-3), 112.10 (C-6), 102.03 (C-8), 85.06 (C-4′), 77.52 (C-3′), 56.46 (C-2′), 55.13 (C-7′, 12′), 48.14 (C-5′), 27.94 (C-8′, 11′), 26.44 (C-9′, 10′), 18.56 (4-CH_3_) ppm; HR-MS, *m*/*z* (I*_rel_.*, %): 328 (1.5), 327 (4, M^+^), 299 (18), 298 (100), 123 (25), 122 (21), 108 (8); calcd for C_20_H_25_NO_3_: 327.1829; found *m*/*z* 327.1831 [M]^+^.

**7-(4-(Azocan-1-yl)but-2-ynyloxy)-2*H*-chromen-2-one** (**28**). White solid. Yield 57% (0.900 g); mp 109–112 °C; IR (KBr) ν: 2260 (C≡C), 1732 (C=O), 1620, 1608 (C=C), 1275, 1122, 1082 (C–O–C) cm^−1^; ^1^H NMR (CDCl_3_, 300 MHz) δ: 7.61 (1H, d, *J* = 9.5 Hz, H-4), 7.36 (1H, d, *J* = 8.6 Hz, H-5), 6.92 (1H, d, *J* = 2.3 Hz, H-8), 6.88 (1H, dd, *J* = 8.6, 2.3 Hz, H-6), 6.25 (1H, d, *J* 9.5 Hz, H-3), 4.77 (2H, br. s, H-2′), 3.35 (2H, br. s, H-5′), 2.54–2.48 (4H, m, H-7′, 13′), 1.58–1.48 (8H, m, H-8′, 9′, 10′, 11′, 12′) ppm; ^13^C NMR (CDCl_3_, 125 MHz) δ: 161.03 (C-2), 160.63 (C-7), 155.49 (C-8a), 143.23 (C-4), 128.58 (C-5), 113.29 (C-6), 113.12 (C-3), 112.78 (C-4a), 102.08 (C-8), 85.91 (C-4′), 76.88 (C-3′), 56.57 (C-2′), 53.09 (C-7′, 13′), 47.66 (C-5′), 27.43 (C-8′, 12′), 27.27 (C-10′), 23.81 (C-9′, 11′) ppm; HR-MS, *m*/*z* (I*_rel_.*, %): 326 (4), 325 (25), 324 (20, M^+^**.**), 214 (10), 165 (11), 164 (100), 163 (24), 162 (26), 133 (27), 126 (16), 112 (91), 105 (14), 55 (28), 53 (24), 51 (12); calcd for C_20_H_22_NO_3_: 324.1594; found *m*/*z* 324.1593 [M]^+^.

**7-(4-(Azocan-1-yl)but-2-ynyloxy)-4-methyl-2*H*-chromen-2-one** (**29**). White solid. Yield 80% (1.35 g); mp 136–139 °C; IR (KBr) ν: 2260 (C≡C), 1727 (C=O), 1610, 1510 (C=C), 1270, 1165, 1088 (C–O–C) cm^−1^; ^1^H NMR (CDCl_3_, 300 MHz) δ: 7.48 (1H, d, *J* = 8.6 Hz, H-5), 6.91 (1H, d, *J* = 2.0 Hz, H-8), 6.89 (1H, dd, *J* = 8.6, 2.0 Hz, H-6), 6.13 (1H, s, H-3), 4.76 (2H, br. s, H-2′), 3.35 (2H, br. s, H-5′), 2.55–2.50 (4H, m, H-7′, 13′), 2.38 (3H, s, CH_3_), 1.54–1.48 (10H, m, H-8′, 9′, 10′, 11′, 12′) ppm; ^13^C NMR (CDCl_3_, 125 MHz) δ: 161.11 (C-2), 160.51 (C-7), 154.952 (C-8a), 152.38 (C-4), 125.37 (C-5), 113.92 (C-4a), 112.91 (C-3), 112.15 (C-6), 102.12 (C-8), 85.89 (C-4′), 77.07 (C-3′), 56.63 (C-2′), 53.12 (C-7′, 13′), 47.70 (C-5′), 27.48 (C-8′, 12′), 27.37 (C-10′), 25.89 (C-9′, 11′), 18.53 (4-CH_3_) ppm; HR-MS, *m*/*z* (I*_rel_.*, %): 340 (8), 339 (33), 338 (23, M^+^.), 282 (8), 176 (13), 165 (12), 164 (98), 163 (65), 148 (10), 112 (100), 108 (16); calcd for C_21_H_24_NO_3_: 338.1751; found *m*/*z* 338.1748 [M]^+^.

**7-(4-(4-Methylpiperazin-1-yl)but-2-ynyloxy)-2*H*-chromen-2-one** (**30**). White solid; yield 46% (0.360 g); mp 106–109 °C; IR (KBr) ν: 2254 (C≡C), 1726 (C=O), 1604, 1504 (C=C), 1340, 1126, 1003, 922 (piperazine), 1277, 1230, 1075 (C–O–C) cm^−1^; ^1^H NMR (CDCl_3_, 300 MHz) δ: 7.61 (1H, d, *J* = 9.5 Hz, H-4), 7.36 (1H, d, *J* = 8.5 Hz, H-5), 6.89 (1H, d, *J* = 2.1 Hz, H-8), 6.88 (1H, dd, *J* = 8.5, 2.1 Hz, H-6), 6.24 (1H, d, *J* = 9.5 Hz, H-3), 4.73 (2H, br. s, H-2′), 3.31 (2H, br. s, H-5′), 2.70–2.40 (8H, m, H-7′, 8′, 10′, 11′), 2.26 (3H, s, NCH_3_) ppm; ^13^C NMR (CDCl_3_, 125 MHz) *δ*: 160.93 (C-2), 160.61 (C-7), 155.55 (C-8a), 143.16 (C-4), 128.81 (C-5), 113.38 (C-3), 113.01 (C-6), 112.88 (C-4a), 102.03 (C-8), 83.65 (C-4′), 78.73 (C-3′), 56.51 (C-2′), 54.77 (C-8′, 10′), 51.77 (C-7′, 11′), 46.91 (C-5′), 45.82 (NCH_3_) ppm; HR-MS, *m*/*z* (I*_rel_.*, %): 313 (2), 312 (2.3, M^+^), 162 (10), 152 (10), 150 (100), 149 (10), 134 (11), 108 (47), 99 (59), 97 (15), 94 (16), 77 (11), 56 (41), 43 (26), 42 (28); calcd for C_18_H_20_N_2_O_3_: 312.1468; found *m*/*z* 312.1466 [M]^+^.

**4-Methyl-7-(4-(4-methylpiperazin-1-yl)but-2-ynyloxy)-2*H*-chromen-2-one** (**31**). White solid; yield 49% (0.380 g); mp 105–107 °C; IR (KBr) ν: 2250 (C≡C), 1725 (C=O), 1610, 1510 (C=C), 1300, 1137, 1003, 920 (piperazine), 1279, 1235, 1070 (C–O–C) cm^−1^; ^1^H NMR (CDCl_3_, 300 MHz) δ: 7.48 (1H, d, *J* = 8.6 Hz, H-5), 6.90 (1H, d, *J* = 2.0 Hz, H-8), 6.88 (1H, dd, *J* = 8.6, 2.0 Hz, H-6), 6.12 (1H, br. s, H-3), 4.73 (2H, br. s, H-2′), 3.31 (2H, br. s, H-5′), 2.77–2.56 (8H, m, H-7′, 8′, 10′, 11′), 2.44 (3H, br. s, 4-CH_3_), 2.26 (3H, s, NCH_3_) ppm; ^13^C NMR (CDCl_3_, 125 MHz) *δ*: 161.04 (C-2), 160.45 (C-7), 154.94 (C-8a), 152.29 (C-4), 125.41 (C-5), 113.97 (C-4a), 112.69 (C-3), 112.20 (C-6), 102.03 (C-8), 83.56 (C-4′), 78.77 (C-3′), 56.44 (C-2′), 54.77 (C-8′, 10′), 51.79 (C-7′, 11′), 46.90 (C-5′), 45.81 (NCH_3_), 18.60 (4-CH_3_) ppm; HR-MS, *m*/*z* (I*_rel_.*, %): 327 (1.6), 326 (2, M^+^), 325 (1.2), 151 (57), 150 (100), 108 (27), 99 (36), 70 (17), 58 (12), 56 (20), 43 (15), 42 (12); calcd for C_19_H_22_N_2_O_3_: 326.1625; found *m*/*z* 326.1633 [M]^+^.

**7-(4-(4-Phenylpiperazin-1-yl)but-2-ynyloxy)-2*H*-chromen-2-one** (**32**). White solid; yield 86% (0.810 g); mp 128–130 °C; IR (KBr) ν: 2254 (C≡C), 1726 (C=O), 1610, 1602, 1500, 825, 765, 694 (C=C, CH-Ar), 1320, 1124, 1001, 910 (piperazine), 1280, 1214, 1070 (C–O–C) cm^−1^; UV (EtOH) λ_max_ (lgε): 275 (4.06), 321 (4.10) nm; ^1^H NMR (CDCl_3_, 300 MHz) δ: 7.60 (1H, d, *J* = 9.5 Hz, H-4), 7.37 (1H, d, *J* = 8.8 Hz, H-5), 7.26 (2H, dd, H-3″, 5″, *J* = 8.2, 7.6 Hz), 6.92 (1H, d, *J* = 2.1 Hz, H-8), 6.90 (1H, dd, *J* = 8.8, 2.1 Hz, H-6), 6.88 (1H, dd, *J* = 7.6, 2.4 Hz, H-4″), 6.81 (2H, dd, *J* = 8.2, 2.4 Hz, H-2″, 6″), 6.24 (1H, d, *J* = 9.5 Hz, H-3), 4.79 (2H, br. s, H-2′), 3.40 (2H, br. s, H-5′), 3.20 (4H, t, *J* = 6.5 Hz, H-8′, 10′), 2.68 (4H, t, *J* = 6.5 Hz, H-7′, 11′) ppm; ^13^C NMR (CDCl_3_, 125 MHz) *δ*: 160.84 (C-2), 160.47 (C-7), 155.46 (C-8a), 150.95 (C-1″), 143.07 (C-4), 129.03 (C-3″, 5″), 128.81 (C-5), 119.62 (C-4″), 115.94 (C-2″, 6″), 113.32 (C-3), 112.95 (C-6), 112.84 (C-4a), 102.05 (C-8), 83.48 (C-4′), 78.91 (C-3′), 56.44 (C-2′), 51.82 (C-8′, 10′), 48.84 (C-7′, 11′), 46.93 (C-5′) ppm; HR-MS, *m*/*z* (I*_rel_.*, %): 376 (3), 375 (21), 374 (79, M^+^), 214 (14), 212 (100), 211 (16), 161 (43), 160 (10), 159 (19), 132 (27), 120 (38), 108 (45), 107 (12), 106 (35), 105 (44), 104 (27), 94 (15), 91 (14), 77 (21), 56 (45); calcd. for C_23_H_22_N_2_O_3_: 374.1625; found *m*/*z* 374.1630 [M]^+^.

**4-Methyl-7-(4-(4-phenylpiperazin-1-yl)but-2-ynyloxy)-2*H*-chromen-2-one** (**33**). White solid; yield 54% (0.520 g); mp 114–117 °C; IR (KBr) ν: 2250 (C≡C), 1729 (C=O), 1603, 1504, 806, 759, 692 (C=C, CH-Ar), 1315, 1138, 1007, 922 (piperazine), 1267, 1203, 1088 (C–O–C) cm^−1^; ^1^H NMR (CDCl_3_, 300 MHz) δ: 7.50 (1H, d, *J* = 8.8 Hz, H-5), 7.25 (2H, dd, H-3″, 5″, *J* = 8.2, 7.6 Hz), 6.92 (1H, d, *J* = 2.3 Hz, H-8), 6.90 (1H, dd, *J* = 8.8, 2.3 Hz, H-6), 6.86 (1H, dd, *J* = 7.6, 2.4 Hz, H-4″), 6.81 (2H, dd, *J* = 8.2, 2.4 Hz, H-2″, 6″), 6.11 (1H, br. s, H-3), 4.80 (2H, br. s, H-2′), 3.41 (2H, br. s, H-5′), 3.20 (4H, t, *J* = 6.3 Hz, H-8′, 10′), 2.69 (4H, t, *J* = 6.3 Hz, H-7′, 11′), 2.36 (3H, br. s, 4-CH_3_) ppm; ^13^C NMR (CDCl_3_, 125 MHz) δ: 160.98 (C-2), 160.31 (C-7), 154.89 (C-8a), 152.23 (C-4), 150.98 (C-1″), 129.96 (C-3″, 5″) 125.41 (C-5), 118.65 (C-4″), 115.95 (C-2″, 6″), 113.97 (C-4a), 112.69 (C-3), 112.20 (C-6), 102.08 (C-8), 83.41 (C-4′), 78.97 (C-3′), 56.44 (C-2′), 51.77 (C-8′, 10′), 48.84 (C-7′, 11′), 46.86 (C-5′), 18.48 (4-CH_3_) ppm; HR-MS, *m*/*z* (I*_rel_.*, %): 389 (12), 388 (49, M^+^), 228 (10), 213 (44), 212 (100), 211 (20), 176 (35), 161 (55), 160 (29), 159 (20), 148 (53), 147 (31), 132 (21), 108 (20), 106 (28), 105 (54), 104 (52), 91 (40), 72 (43), 56 (29), 53 (22), 52 (15); calcd for C_24_H_24_N_2_O_3_: 388.1781; found *m*/*z* 388.1780 [M]^+^.

**7-(4-(4-(4-Hydroxyphenyl)piperazin-1-yl)but-2-ynyloxy)-2*H*-chromen-2-one** (**35**). White solid; yield 40% (0.235 g); mp 103–105 °C; IR (KBr) ν: 3531 (OH), 2235 (C≡C), 1699 (C=O), 1620, 1517, 833, 814, 761, 748 (C=C, CH-Ar), 1342, 1120, 1000, 918 (piperazine), 1259, 1130, 1080 (C–O–C) cm^−1^; ^1^H NMR (CD_3_)_2_SO, 300 MHz) δ: 8.84 (1H, s, OH), 7.98 (1H, d, *J* = 9.6 Hz, H-4), 7.64 (1H, d, *J* = 8.6 Hz, H-5), 7.07 (1H, d, *J* = 2.0 Hz, H-8), 7.00 (1H, dd, *J* = 8.6, 2.0 Hz, H-6), 6.76 (2H, d, *J* = 8.5 Hz, H-3″, 5″), 6.64 (2H, d, *J* = 8.5 Hz, H-2″, 6″), 6.29 (1H, d, *J* = 9.6 Hz, H-3), 4.85 (2H, br. s, H-2′), 3.43 (2H, br. s, H-5′), 2.94 (4H, m, H-8′, 10′), 2.59 (4H, m, H-7′, 11′) ppm; ^13^C NMR (CD_3_)_2_SO, 125 MHz *δ*: 160.55 (C-2), 160.52 (C-7), 155.43 (C-8a), 151.28 (C-1″), 144.55 (C-4), 144.25 (C-4″), 129.80 (C-5), 128.68 (C-5), 118.17 (C-3″, 5″), 116.71 (C-2″, 6″), 113.32 (C-3), 113.17 (C-6), 113.11 (C-4a), 102.22 (C-8), 83.51 (C-4′), 79.27 (C-3′), 56.68 (C-2′), 51.61 (C-8′, 10′), 49.95 (C-7′, 11′), 46.32 (C-5′) ppm; HR-MS, *m*/*z* (I*_rel_.*, %): 392 (5), 391 (23), 390 (100, M^+^), 229 (27), 206 (33), 211 (16), 177 (45), 162 (43), 148 (24), 136 (73), 134 (52), 122 (30), 121 (55), 120 (43), 108 (28), 84 (79), 66 (83), 56 (36), 46 (36), 43 (25); calcd. for C_23_H_22_N_2_O_4_: 390.1574; found *m*/*z* 390.1572 [M]^+^.

**7-(4-(4-(4-Hydroxyphenyl)piperazin-1-yl)but-2-ynyloxy)-4-methyl-2*H*-chromen-2-one** (**36**). White solid; yield 39% (0.220 g); mp 99–102 °C; IR (KBr) ν: 3392 (OH), 2235 (C≡C), 1724 (C=O), 1614, 1514, 827, 750 (C=C, CH-Ar), 1348, 1110, 1003, 922 (piperazine), 1265, 1128, 1084 (C–O–C) cm^−1^; ^1^H NMR (CDCl_3_, 300 MHz) δ: 7.47 (1H, d, *J* = 8.8 Hz, H-5), 6.91 (1H, d, *J* = 2.0 Hz, H-8), 6.89 (1H, dd, *J* = 8.8, 2.0 Hz, H-6), 6.79 (2H, d, *J* = 8.6 Hz, H-3″, 5″), 6.72 (2H, d, *J* = 8.6 Hz, H-2″, 6″), 6.11 (1H, br. s, H-3), 4.76 (2H, br. s, H-2′), 3.38 (2H, br. s, H-5′), 3.06 (4H, t, *J* = 6.3 Hz, H-8′, 10′), 2.67 (4H, t, *J* = 6.3 Hz, H-7′, 11′), 2.34 (3H, br. s, 4-CH_3_) ppm; ^13^C NMR (CDCl_3_, 125 MHz) δ: 161.40 (C-2), 160.34 (C-7), 153.81 (C-8a), 152.66 (C-4), 150.21 (C-1″), 144.83 (C-4″), 126.46 (C-5), 118.50 (C-3″, 5″), 115.83 (C-2″, 6″), 113.93 (C-4a), 112.80 (C-3), 112.09 (C-6), 101.99 (C-8), 83.25 (C-4′), 79.11 (C-3′), 56.33 (C-2′), 51.80 (C-8′, 10′), 50.37 (C-7′, 11′), 46.89 (C-5′), 18.65 (4-CH_3_) ppm; HR-MS, *m*/*z* (I*_rel_.*, %): 406 (5), 405 (27), 404 (100, M^+^), 229 (27), 228 (35), 227 (14), 177 (41), 176 (29), 175 (15), 148 (50), 147 (28), 136 (27), 122 (24), 121 (50), 120 (45), 108 (35), 107 (15), 91 (15), 78 (21), 63 (24), 56 (33); calcd for C_24_H_24_N_2_O_4_: 404.1731; found *m*/*z* 404.1734 [M]^+^.

**7-(4-(4-(Furan-2-carbonyl)piperazin-1-yl)but-2-ynyloxy)-2*H*-chromen-2-one** (**37**). White solid; yield 41% (0.220 g); mp 112–114 °C; IR (KBr) ν: 2270 (C≡C), 1734 (C=O), 1645 (C=O), 1612, 1500, 1484 (C=C), 1314, 1120, 1001, 938 (piperazine), 1277, 1226, 1056 (C–O–C) cm^−1^; ^1^H NMR (CDCl_3_, 300 MHz) δ: 7.60 (1H, d, *J* = 9.6 Hz, 1H, H-4), 7.46 (1H, d, *J* = 1.6 Hz, H-5″), 7.35 (1H, d, *J* = 8.4 Hz, H-5), 6.95 (1H, d, *J* = 3.4 Hz, H-3″), 6.88 (1H, d, *J* = 2.0 Hz, H-8), 6.86 (1H, dd, *J* = 8.4, 2.0 Hz, H-6), 6.45 (1H, dd, *J* = 3.4, 1.6 Hz, H-4″), 6.19 (1H, d, *J* = 9.6 Hz, H-3), 4.76 (2H, br. s, 2H, H-2′), 3.75–3.83 (4H, m, H-8′, 10′), 3.37 (2H, br. s, H-5′), 2.55 (4H, m, H-7′, 11′) ppm; ^13^C NMR (CDCl_3_, 125 MHz) δ: 160.97 (C-2), 160.48 (C-7), 158.89 (C-8a), 155.52 (C=O), 147.66 (C-2″), 143.86 (C-5″), 143.23 (C-4), 128.72 (C-5), 116.60 (C-3″), 113.44 (C-3), 112.97 (C-4a), 112.94 (C-4″), 111.21 (C-6), 102.11 (C-8), 83.01 (C-4′), 79.25 (C-3′), 56.49 (C-2′), 51.82 (C-7′, 11′), 46.91 (C-5′), 42.56 (C-8′, 10′) ppm; HR-MS, *m*/*z* (I*_rel_.*, %): 393 (2), 392 (7, M^+^), 391 (6), 268 (18), 231 (32), 230 (72), 138 (38), 135 (12), 119 (23), 106 (31), 95 (100), 94 (13), 69 (11), 56 (14). 42 (12); calcd for C_22_H_20_N_2_O_5_: 392.1367; found *m*/*z* 392.1368 [M]^+^.

**7-(4-(4-(Furan-2-carbonyl)piperazin-1-yl)but-2-ynyloxy)-4-methyl-2*H*-chromen-2-one** (**38**). White solid; yield 49% (0.276 g); mp 111–113 °C; IR (KBr) ν: 2270 (C≡C), 1730 (C=O), 1648 (C=O), 1620, 1510, 1487 (C=C), 1318, 1132, 1003, 915 (piperazine), 1267, 1203, 1088, 1047 (C–O–C) cm^−1^; UV (EtOH) λ_max_ (lgε): 290 (3.90), 320 (4.14) nm; ^1^H NMR (CDCl_3_, 300 MHz) δ: 7.47 (1H, d, *J* = 8.6 Hz, H-5), 7.45 (1H, d, *J* = 1.5 Hz, H-5″), 6.95 (1H, d, *J* = 3.2 Hz, H-3″), 6.88 (1H, d, *J* = 2.0 Hz, H-8), 6.86 (1H, dd, *J* = 8.6, 2.0 Hz, H-6), 6.44 (1H, dd, *J* = 3.2, 1.5 Hz, H-4″), 6.11 (1H, br. s, H-3), 4.76 (2H, br. s, 2H, H-2′), 3.75–3.83 (4H, m, H-8′, 10′), 3.38 (2H, br. s, H-5′), 2.51–2.59 (4H, m, H-7′, 11′), 2.30 (3H, br. s, 4-CH_3_) ppm; ^13^C NMR (CDCl_3_, 125 MHz) δ: 161.05 (C-2), 160.22 (C-7), 158.84 (C-8a), 154.82 (C=O), 152.42 (C-4), 147.54 (C-2″), 143.63 (C-5″), 125.52 (C-5), 116.40 (C-3″), 114.01 (C-4a), 112.25 (C-3), 112.15 (C-4″), 111.20 (C-6), 102.02 (C-8), 82.77 (C-4′), 79.31 (C-3′), 54.29 (C-2′), 51.83 (C-7′, 11′), 46.84 (C-5′), 42.31 (C-8′, 10′), 18.54 (4-CH_3_) ppm; HR-MS, *m*/*z* (I*_rel_.*, %): 407 (0.7), 406 (1.5, M^+^), 405 (1.4), 230 (14), 148 (16), 138 (9), 147 (15), 119 (9), 95 (100), 91 (15), 57 (11), 56 (9). 43 (9), 39 (34); calcd for C_23_H_22_N_2_O_5_: 406.1523; found *m*/*z* 406.1514 [M]^+^.

**7-(4-(4-(Tetrahydrofuran-2-carbonyl)piperazin-1-yl)but-2-ynyloxy)-2*H*-chromen-2-one (39)**. Colorless oil; yield 28% (0.175 g); IR (KBr) ν: 2286 (C≡C), 1709 (C=O), 1648 (C=O), 1614, 1570, 1506 (C=C), 1315, 1132, 1003, 920 (piperazine), 1230, 1123, 1080, 1045 (C–O–C) cm^−1^; ^1^H NMR (CDCl_3_, 300 MHz) δ: 7.63 (1H, d, *J* = 9.6 Hz, H-4), 7.27 (1H, d, *J* = 8.5 Hz, H-5), 6.89 (1H, d, *J* = 2.0 Hz, H-8), 6.87 (1H, dd, *J* = 8.5, 2.0 Hz, H-7), 6.25 (1H, d, *J* = 9.6 Hz, H-3), 4.76 (2H, br. s, H-2′), 4.61–4.53 (1H, m, H-2″), 3.95–3.88 (1H, m, H-5″), 3.86–3.79 (1H, m, H-5″), 3.72–3.60, 3.58–3.48 (2H, both m, H-8′, 10′), 3.34 (2H, br. s, H-5′), 2.52–2.44 (4H, m, H-7′, 11′), 2.30–2.22 (1H, m, H-3″), 2.10–1.90 (2H, m, H-4″), 1.87–1.80 (1H, m, H-3″) ppm; ^13^C NMR (CDCl_3_, 125 MHz) δ: 169.70 (C=O), 160.97 (C-2), 160.46 (C-7), 155.45 (C-8a), 143.26 (C-4), 128.71 (C-5), 113.39 (C-3), 112.96 (C-4a), 112.90 (C-6), 101.96 (C-8), 83.04 (C-4′), 79.08 (C-3′), 75.63 (C-2″), 68.99 (C-5″), 56.36 (C-2′), 51.99 (C-8′, 10′), 51.51 (C-7′, 11′), 46.54 (C-5′), 45.03 (C-2″), 41.59 (C-5″), 28.39 (C-3″), 25.61 (C-4″) ppm; HR-MS, *m*/*z* (I*_rel_.*, %): 397 (3.7), 396 (8, M^+^), 235 (11), 234 (23), 142 (10), 136 (11), 135 (15), 119 (12), 108 (14), 106 (16), 94 (17), 71 (100), 56 (19), 43 (47), 42 (16); calcd for C_22_H_24_N_2_O_5_: 396.1680; found *m*/*z* 396.1677 [M]^+^.

**7-[4-(4-Benzo[*d*][1,3]dioxol-5-ylmethyl)piperazin-1-yl)but-2-ynyloxy)-2*H*-chromen-2-one** (**41**). Colorless oil; yield 44% (0.280 g); IR (KBr) ν: 2258 (C≡C), 1735 (C=O), 1614, 1504, 835, 809, 755, 696 (C=C, CH-Ar), 1306, 1132, 1009, 931 (piperazine), 1256, 1210, 1068, 1045 (C–O–C) cm^−1^; UV (EtOH) λ_max_ (lgε): 293 (4.06), 321 (4.15) nm; ^1^H NMR (CDCl_3_, 300 MHz) δ: 7.66 (1H, d, *J* = 9.6 Hz, H-4), 7.41 (1H, d, *J* = 8.4 Hz, H-5), 6.94 (1H, d, *J* = 2.0 Hz, H-8), 6.93 (1H, dd, *J* = 8.4, 2.0 Hz, H-6), 6.91 (1H, d, *J* = 7.8 Hz, H-7″), 6.78 (1H, d, *J* = 7.8 Hz, H-6″), 6.76 (1H, br. s, H-4″), 6.28 (1H, d, *J* = 9.6 Hz, H-3), 5.97 (2H, s, H-2″), 4.85 (2H, br. s, H-2′), 3.48 (2H, s, H-12′), 3.35 (2H, br. s, H-5′), 2.69–2.47 (8H, m, H-7′, 8′, 10′, 11′) ppm; ^13^C NMR (CDCl_3_, 125 MHz) δ: 161.03 (C-2), 160.58 (C-7), 155.51 (C-8a), 147.51 (C-3a″), 146.44 (C-7a″), 143.25 (C-4), 131.79 (C-5″), 128.63 (C-5), 122.11 (C-6″), 113.33 (C-3), 112.95 (C-4a), 112.86 (C-6), 109.86 (C-4″), 107.69 (C-7″), 101.96 (C-8), 100.76 (C-2″), 83.78 (C-4′), 78.56 (C-3′), 62.50 (C-12′), 56.50 (C-2′), 52.58 (C-8′, 10′), 51.92 (C-7′, 11′), 46.87 (C-5′) ppm; HR-MS, *m*/*z* (I*_rel_.*, %): 432 (1.2, M^+^), 431 (0.5), 271 (51), 270 (12), 248 (18), 136 (19), 135 (100), 85 (18), 84 (43), 83 (65), 47 (17), 35 (15); calcd for C_25_H_24_N_2_O_5_: 432.1680; found *m*/*z* 432.1682 [M]^+^.

**7-[4-(4-Benzo[*d*][1,3]dioxol-5-ylmethyl)piperazin-1-yl)but-2-ynyloxy)-4-methyl-2*H*-chromen-2-one** (**42**). White solid; yield 40% (0.320 g); mp 108–110 °C; IR (KBr) ν: 2262 (C≡C), 1724 (C=O), 1610, 1506, 833, 811, 750, 707 (C=C, CH-Ar), 1312, 1139, 1005, 913 (piperazine), 1265, 1203, 1070, 1043 (C–O–C) cm^−1^; UV (EtOH) λ_max_ (lgε): 284 (3.34), 338 (3.62) nm; ^1^H NMR (CDCl_3_, 300 MHz) δ: 7.41 (1H, d, *J* = 8.8 Hz, H-5), 6.91 (1H, d, *J* = 2.0 Hz, H-8), 6.89 (1H, dd, *J* = 8.8, 2.0 Hz, H-6), 6.79 (1H, d, *J* = 7.8 Hz, H-7″), 6.73 (1H, d, *J* = 7.8 Hz, H-6″), 6.71 (1H, br. s, H-4″), 6.12 (1H, br. s, H-3), 5.91 (2H, s, H-2″), 4.76 (2H, br. s, H-2′), 3.38 (2H, s, H-12′), 3.31 (2H, br. s, H-5′), 2.62–2.39 (8H, m, H-7′, 8′, 10′, 11′), 2.38 (3H, br. s, 4-CH_3_) ppm; ^13^C NMR (CDCl_3_, 125 MHz) δ: 161.11 (C-2), 160.39 (C-7), 154.90 (C-8a), 152.40 (C-4), 147.45 (C-3a″), 146.41 (C-7a″), 131.76 (C-5″), 125.44 (C-5), 122.10 (C-6″), 113.94 (C-3), 112.68 (C-4a), 112.21 (C-6), 109.37 (C-4″), 107.73 (C-7″), 102.03 (C-8), 100.75 (C-2″), 83.76 (C-4′), 78.64 (C-3′), 62.54 (C-12′), 56.50 (C-2′), 52.63 (C-8′, 10′), 51.91 (C-7′, 11′), 47.03 (C-5′), 18.61 (4-CH_3_) ppm; HR-MS, *m*/*z* (I*_rel_.*, %): 447 (0.5), 446 (1.8, M^+^), 271 (47), 270 (21), 136 (10), 135 (100), 77 (13); calcd for C_26_H_26_N_2_O_5_: 446.1836; found *m*/*z* 446.1832 [M]^+^.

**7-(4-(3,4-Dihydroisoquinolin-2(1*H*)-yl)but-2-ynyloxy)-2*H*-chromen-2-one (44).** White solid; yield 26% (0.220 g); mp 95–97 °C; IR (KBr) ν: 2255 (C≡C), 1699 (C=O), 1614, 1507, 838, 737 (C=C, CH-Ar), 1272, 1130, 1068 (C–O–C) cm^−1^; ^1^H NMR (CDCl_3_, 300 MHz) δ: 7.58 (1H, d, *J* = 9.6 Hz, 1H, H-4), 7.34 (1H, d, *J* = 8.6 Hz, H-5), 7.11–7.04 (3H, m, H-6″, 7″, 8″), 6.95 (1H, dd, *J* = 7.2, 1.8 Hz, H-5″), 6.91 (1H, d, *J* = 2.0 Hz, H-8), 6.87 (1H, dd, *J* = 8.6, 2.0 Hz, H-6), 6.23 (1H, d, *J* = 9.6 Hz, H-3), 4.78 (2H, br. s, 2H, H-2′), 3.67 (2H, br. s, H-1″), 3.51 (2H, br. s, H-5′), 2.91–2.83 (2H, m, H-4″), 2.80–2.71 (2H, m, H-3″) ppm; ^13^C NMR (CDCl_3_, 125 MHz) δ: 160.90 (C-2), 160.47 (C-7), 155.48 (C-8a), 143.10 (C-4), 134.11 (C-4a″), 133.16 (C-8a″), 128.60 (C-5), 128.50 (C-5″), 126.38 (C-8″), 126.02 (C-6″), 125.51 (C-7″), 113.40 (C-3), 113.08 (C-6), 112.89 (C-4a), 102.13 (C-8), 83.67 (C-4′), 78.81 (C-3′), 56.48 (C-1″), 54.21 (C-2′), 49.67 (C-3″), 46.86 (C-5′), 29.02 (C-4″) ppm; HR-MS, *m*/*z* (I*_rel_.*, %): 346 (7), 345 (34, M^+^), 298 (12), 184 (20), 183 (100), 182 (41), 134 (11), 133 (29), 132 (19), 131 (16), 130 (28), 105 (16), 104 (81), 103 (23). 78 (18), 77 (19), 51 (15); calcd for C_22_H_19_NO_3_: 345.1360; found *m*/*z* 345.1363 [M]^+^.

**7-(4-(3,4-Dihydroisoquinolin-2(1*H*)-yl)but-2-ynyloxy)-4-methyl-2*H*-chromen-2-one (45).** White solid; yield 33% (0.162 g); mp 122–125 °C; IR (KBr) ν: 2285 (C≡C), 1740 (C=O), 1610, 1510, 851, 810, 740, 706 (C=C, CH-Ar), 1275, 1138, 1062 (C–O–C) cm^−1^; ^1^H NMR (CDCl_3_, 300 MHz) δ: 7.45 (1H, d, *J* = 8.5 Hz, H-5), 7.09–7.04 (3H, m, H-6″, 7″, 8″), 6.93 (1H, dd, *J* = 7.3, 1.8 Hz, H-5″), 6.91 (1H, d, *J* = 2.0 Hz, H-8), 6.88 (1H, dd, *J* = 8.5, 2.0 Hz, H-6), 6.10 (1H, br. s, H-3), 4.78 (2H, br. s, 2H, H-2′), 3.65 (2H, br. s, H-1″), 3.51 (2H, br. s, H-5′), 2.91–2.83 (2H, m, H-4″), 2.79–2.71 (2H, m, H-3″), 2.34 (3H, br. s, 4-CH_3_) ppm; ^13^C NMR (CDCl_3_, 125 MHz) δ: 160.95 (C-2), 160.36 (C-7), 154.94 (C-8a), 152.17 (C-4), 134.21 (C-4a″), 133.47 (C-8a″), 128.50 (C-5), 126.36 (C-5″), 126.02 (C-8″), 125.51 (C-6″), 125.37 (C-7″), 114.03 (C-4a), 112.74 (C-3), 112.24 (C-6), 102.31 (C-8), 83.68 (C-4′), 78.93 (C-3′), 56.50 (C-2′), 51.24 (C-1″), 49.76 (C-3″), 46.89 (C-5′), 29.04 (C-4″), 18.48 (4-CH_3_) ppm; HR-MS, *m*/*z* (I*_rel_.*, %): 360 (1), 359 (6, M^+^), 358 (2), 184 (14), 183 (42), 182 (100), 130 (13), 104 (10); calcd for C_23_H_21_NO_3_: 359.1516; found *m*/*z* 359.1511 [M]^+^.

### 4.3. Cell Culture and Cytotoxicity Assay

The HPV-negative human cervical cancer cell line C33 A (ATCC HTB-31), HPV16-positive human cervical cancer cell line CaSki (ATCC CRM-CRL-1550), and breast cancer adenocarcinoma MCF-7 (ATCC HTB-22) cell lines were obtained from the American Type Culture Collection. Normal epithelial VERO cells (Cat. No T8299) derived from the kidney of an African green monkey were used as the non-cancer control. This cell line was obtained from the cell collection of the State Research Center for Virology and Biotechnology “Vector” of Rospotrebnadzor, Koltsovo, Novosibirsk Region. The cells were cultured in DMEM/F12 medium containing 10% embryonic calf serum, L-glutamine (2 mmol/L), and gentamicin (80 µg/mL) in a CO_2_ incubator at 37 °C. The tested compounds (**5**, **7**, **9**, **14**–**21**, **23**–**26**, **28**–**33**, **35**–**39**, **41**, **42**, **44**, and **45**) and a reference drug, doxorubicin, were dissolved in DMSO and added to the cell culture at the required concentrations (the residual concentration of DMSO in the sample does not exceed 1%). Three wells were used for each concentration. The cells that were incubated with only DMSO without the compounds were used as controls. Cells were placed in 96-well microplates and cultivated at 37 in 5% CO_2_/95% air for 48 h. Cell viability was assessed through an MTT [3-(4,5-dimethylthiazol-2-yl)-2,5-phenyl-2H-tetrazolium bromide] conversion assay [48], where 1% MTT was added to each well. Four hours later, the medium was removed, leaving the formazan crystals, and isopropanol was added and mixed for 15 min. The optical density of the samples was measured on a Thermo Multiskan FC spectrophotometer (Thermo Fisher Scientific, Waltham, MA, USA) at a wavelength of 570 nm, with a reference at 670 nm. The 50% cytotoxic dose (GI_50_) of each compound (i.e., the compound concentration that lowers the amount of cells to 50% in a culture or decreases the optical density two-fold as compared to the control wells) was calculated from the obtained data. All data are presented as the mean ± SEM from at least three independent experiments. Statistical analysis of the results was performed using Microsoft Excel 2007, STATISTICA 6.0, and GraphPad Prism 5.0 programs, and significance was defined as *p* < 0.05 (*t*-test). The results are given as the average value ± standard deviation. SI (selectivity index; a ratio of the mean GI_50_ (non-cancer cells) to the mean GI_50_ (cancer cells)) was calculated using Microsoft Excel 2019 (Microsoft, Redmond, WA, USA). All experiments were repeated 3 times. At least triplicate cultures were scored for each experimental point. Cell viability was calculated as a percentage of vehicle control.

### 4.4. Molecular Modeling

The three-dimensional structures of coumarin derivatives (compounds **7**, **9**, **14**, **16**, **23**, **25**, **28**, **36**, **37**, **38**, **42**, and **45**) were constructed using ChemOffice 2016 software (PerkinElmer Informatics, Waltham, MA, USA) and subsequently energy-minimized by employing the MM2 force field to refine their geometries. The crystal structure of the intramolecular human telomeric DNA G-quadruplex, co-crystallized with naphthalene diimide ligand MM41 [43], was downloaded from the Protein Data Bank (PDB ID: 3UYH).

Molecular docking was conducted using Molegro Virtual Docker (MVD) version 6.0 (CLC bio, Qiagen, Aarhus, Denmark). The G-quadruplex structure and ligand models were imported into the MVD workspace. To define the search space, a spherical area with a radius of 10 Å was placed at the centroid of the co-crystallized MM41 molecule, encompassing the binding site. For each ligand, 500 independent docking runs were executed with the “Internal HBond” and “Sp^2^-Sp^2^ Torsions” ligand evaluation options enabled to account for possible intramolecular hydrogen bonding and torsional flexibility in π-electron systems. The MolDock scoring function was applied to evaluate the docked poses, which were further re-evaluated using the rerank score [44]. The results were analyzed and visualized with the MVD built-in tools.

## 5. Conclusions

In summary, an efficient method for the modification of 6-hydroxycoumarin, umbelliferone, and hymecromone derivatives has been developed via a three-component reaction of the corresponding 6- or 7-propargyl-substituted coumarins with secondary amines and formaldehyde in the presence of a copper catalyst. This A^3^-coupling procedure proved to be rapid and effective, enabling the first synthesis of a series of 6- and 7-(4-aminobut-2-ynyloxy)coumarins. Structure–activity relationship (SAR) data indicate that the presence of the 4-aminobut-2-ynyl moiety plays a crucial role in the ability of these compounds to inhibit tumor cell growth, while its attachment at the C-6 or C-7 positions of the coumarin core does not hinder the retention of cytotoxicity. Coumarin derivatives (**16**, **17**, **35**, **36**, **38**, **39**, **41**, and **42**) having a (piperazin-1-ylbut-2-ynyloxy) side chain at the C-6 or C-7 positions inhibited the growth of tumor cells with greater selectivity than a positive control, doxorubicin (GI_50_ = 4.3–10.5 μM, SI = 9.6–19.8). Replacing the piperazin-1-yl residue at the 4 position of the but-2-ynyloxy side chain with a dialkylamino group led to a marked decrease in cytotoxic properties, as demonstrated by compounds **5**, **7**, and **18**–**24** (GI_50_ = 21.5–45.8 μM). The nature of the substituent at the 4 position of the piperazine fragment is of significant importance for selectivity: compounds with furanocarbonyl and *p*-hydroxyphenyl substituents showed higher selectivity toward C33 A cervical cancer cells (SI 14.1–16.3), whereas derivatives with a tetrahydrofuranocarbonyl substituent were more selective toward MCF-7 cells (SI 16.1–19.7). Consequently, compounds **16**, **17**, and **36**–**39** can serve as a promising starting point for developing novel, diversified antitumor tools. Based on these promising yet preliminary data, further studies (e.g., expanded cytotoxicity assessment and selectivity profiling, characterization of mechanisms of action, and investigation of target interactions) will be beneficial for a comprehensive evaluation of 6- or 7-(piperazin-1-ylbut-2-ynyloxy)-substituted coumarins. It should be noted that the proposed interaction with DNA G-quadruplexes is currently only a computational hypothesis based solely on molecular docking results. Biophysical confirmation by methods such as the G4-FID (fluorescent intercalator displacement) assay or time-resolved fluorescence correlation spectroscopy will be needed to establish physical binding in vitro. In addition, because the coumarin nucleus is a privileged scaffold known to engage multiple established anticancer targets (including kinases, aromatase, and HSP90), we do not rule out the possibility that the compounds reported here exert cytotoxicity through a multitarget mechanism. Forthcoming work will therefore begin with biophysical validation and then proceed to targeted cellular experiments, for example, specific knockdown models, that can distinguish G-quadruplex-dependent from independent antitumor pathways.

## Figures and Tables

**Table 1:** Concentrations of half-maximal inhibition (GI_50_ ± SEM, μM) on cancer cell lines and VERO cells for coumarin derivatives **5**, **7**, **9**, **14**–**21**, **23**–**26**, **28**–**33**, **35**–**39**, **41**, **42**, **44**, and **45** and doxorubicin.

Compound			Type of Cells ^[a,b]^						
			Cytotoxicity (GI_50_, μM) Against Cell Lines			Selectivity Index (SI) ^[c]^			VERO
			C33 A	CaSki	MCF-7	C33 A	CaSki	MCF-7	
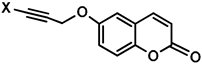									
**5**	**X**= CH_2_N(*i*-Pr)_2_		34.5 ± 3.6	37.9 ± 2.8	21.5 ± 0.8				>100
**7**	**X** = CH_2_N(Pr)_2_		38.6 ± 2.9	>100	22.1 ± 2.1				>100
**9**	**X** = 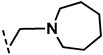		15.1 ± 0.8	27.4 ± 1.1	24.1 ± 1.2	5.9	3.4	3.7	90.1 ± 8.5
**14**	**X** = 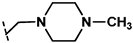		20.3 ± 1.6	23.2 ± 1.4	12.5 ± 0.8	4.1	3.6	6.7	84.1 ± 6.8
**15**	**X** = 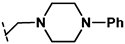		24.8 ± 3.2	16.2 ± 0.9	15.1 ± 1.2				>100
**16**	X = 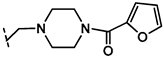		6.1 ± 0.8	12.8 ± 0.8	5.9 ± 0.7	14.5	6.9	15.0	88.6 ± 4.6
**17**	X = 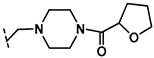		11.2 ± 0.9	19.5 ± 2.6	6.2 ± 1.0	8.9	5.1	16.1	>100
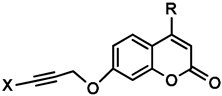									
**18**	**X** = CH_2_N(*i*-Pr)_2_	**R = H**	42.1 ± 2.4	30 ± 4.6	27.1 ± 2.7				NT
**19**	**X** = CH_2_N(*i*-Pr)_2_	**R = CH_3_**	31.2 ± 1.4	45.8 ± 4.2	38.3 ± 1.1				NT
**20**	**X** = CH_2_N(Pr)_2_	**R = H**	27.1 ± 1.1	>100	29.6 ± 1.6				>100
**21**	**X** = CH_2_N(Pr)_2_	**R = CH_3_**	31.3 ± 4.1	34.6 ± 3.8	18.2 ± 0.8				NT
**23**	**X** = CH_2_N(*n*-Bu)_2_	**R = H**	48.8 ± 4.3	35.3 ± 3.6	31.2 ± 1.6				>100
**24**	**X** = CH_2_N(*n*-Bu)_2_	**R = CH_3_**	23.6 ± 1.9	25.1 ± 2.3	28.6 ± 1.5				>100
**25**	**X =** 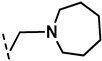	**R = H**	29.1 ± 4.2	32.6 ± 2.5	16.5 ± 0.9	3.1	2.8	5.3	89.1 ± 6.5
**26**		**R =CH_3_**	20.1 ± 1.8	17.4 ± 1.1	12.1 ± 0.6	4.8	5.5	8.1	96.4 ± 7.1
**28**	**X =** 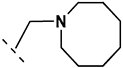	**R = H**	28.5 ± 1.2	16.2 ± 0.8	18.8 ± 1.4				>100
**29**		**R = CH_3_**	24.9 ± 1.8	9.6 ± 0.8	15.4 ± 0.8	3.7	9.5	5.9	91.1 ± 7.3
**30**	**X =** 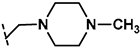	**R = H**	18.1 ± 0.6	19.5 ± 1.9	13.1 ± 1.2	5.1	4.6	6.9	90.6 ± 3.9
**31**		**R = CH_3_**	23.5 ± 1.2	21.4 ± 2.5	17.3 ± 1.4				>100
**32**	**X =** 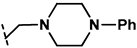	**R = H**	24.8 ± 3.2	17.4 ± 1.1	11.2 ± 0.8	3.5	4.8	7.7	86.2 ± 8.4
**33**		**R = CH_3_**	13.3 ± 0.8	20.2 ± 1.0	12.1 ± 0.9				>100
**35**	**X =** 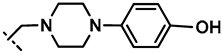	**R = H**	7.1 ± 0.5	15.1 ± 0.8	10.5 ± 0.8	14.1	6.6	9.6	>100
**36**		**R = CH_3_**	6.2 ± 0.9	17.9 ± 0.6	8.6 ± 0.5	16.1	5.6	11.6	>100
**37**	**X =** 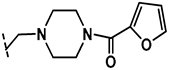	**R = H**	11.8 ± 1.4	8.9 ± 0.6	6.6 ± 0.9	8.5	11.3	15.1	>100
**38**		**R = CH_3_**	5.2 ± 0.3	9.4 ± 0.9	4.3 ± 0.7	16.3	9.1	19.8	85.1 ± 4.1
**39**		**R = H**	9.1 ± 0.5	11.1 ± 0.9	5.1 ± 0.6	10.9	9.0	19.7	>100
**41**	**X =** 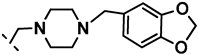	**R = H**	20.4 ± 1.3	7.5 ± 0.2	4.7 ± 0.9	4.4	11.9	19.4	89.8 ± 8.7
**42**		**R = CH_3_**	10.2 ± 0.9	6.8 ± 0.9	5.8 ± 0.7	9.8	14.7	17.2	>100
**44**	**X =** 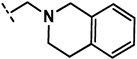	**R = H**	12.5 ± 1.5	18.6 ± 0.8	19.4 ± 0.6				>100
**45**		**R = CH_3_**	15.1 ± 1.0	17.2 ± 1.5	12.5 ± 0.9				>100
**Doxorubicin**			2.5 ± 0.8	10.1 ± 0.7	5.2 ± 0.8	3.2	0.8	1.5	8.0 ± 1.8

^[a]^ GI_50_: concentration at which 50% growth inhibition of tumor cells is observed after 48 h incubation. ^[b]^ The experimental results are given as the data-averaged values obtained from three independently conducted experiments. ^[c]^ Selectivity index (SI): a ratio of the mean GI_50_ (non-cancer cells) to the mean GI_50_ (cancer cells).

**Table 2:** Results of molecular docking of coumarin derivatives in the G-quadruplex binding site.

Ligand	Rerank Score *	Ligand	Rerank Score *
**7**	−57.39	**28**	−54.61
**9**	−61.12	**36**	−70.20
**14**	−63.93	**37**	−69.69
**16**	−79.66	**38**	−74.42
**23**	−51.41	**42**	−80.47
**25**	−62.86	**45**	−61.03

* The rerank score (unitless) is a post-docking scoring function that combines the terms of the MolDock Score with additional steric (Lennard–Jones) terms [45].

## Data Availability

Data are contained within the article.

## Supplementary Materials

The following supporting information can be downloaded at: https://www.mdpi.com/article/10.3390/molecules31183222/s1, (Figures S1–S60: copies of 1H and 13C NMR spectra of all new compounds).
