# Supplementary material for: Coumarin Compounds with 6- or 7-(4-Aminobutynyloxy) Substituent: Synthesis, In Vitro Cytotoxicity and In Silico DNA Binding Studies
Source: Molecules. 2026 Sep 12;31(18):3222. doi: 10.3390/molecules31183222 (PMC13609057; doi:10.3390/molecules31183222)
Supplement: Supplementary file 1 [file molecules-31-03222-s001.zip › molecules-4504142-supplementary.pdf]

SUPPLEMENTARY MATERIAL

Title: Coumarin Compounds with 6- or 7-(4-Aminobutynyloxy) Substituent: Synthesis, In Vitro Cytotoxicity and In Silico DNA Binding Studies

Anarkul S. Kishkentayeva [1,*], Mohammad S. Hamad [2], Victor A. Savelyev [3], Zhasmin A. Boyaubayeva [1], Andrey I. Khlebnikov [4], Andrey G. Pokrovsky [2], Yurii V. Gatilov [3], Almagul S. Makhmutova [1] and Elvira E. Shults [3,*]

[1] School of Pharmacy, Karaganda Medical University, Karaganda 100012, Kazakhstan
[2] Institute of Medicine and Medical Technologies, Novosibirsk State University, Pirogova Str., 1, 630090 Novosibirsk, Russia
[3] N.N. Vorozhtsov Novosibirsk Institute of Organic Chemistry, Siberian Branch of the Russian Academy of Sciences, 630090 Novosibirsk, Russia
[4] Kizhner Research Center, Tomsk Polytechnic University, 634050 Tomsk, Russia
* Correspondence: anar_kish@mail.ru (A.S.K.); schultz@nioch.nsc.ru (E.E.S.)

Copies of ^{1}H and ^{13}C NMR spectra for all new compounds.

---

1 Figure S1. ^{1}H NMR spectrum of 6-(4-(diisopropylamino)but-2-ynyloxy)-2*H*-chromen-2-one (**5**) ($CDCl_3$, 300 MHz).
2 Figure S2. ^{13}C NMR spectrum of 6-(4-(diisopropylamino)but-2-ynyloxy)-2*H*-chromen-2-one (**5**) ($CDCl_3$, 125 MHz).
3 Figure S3. ^{1}H NMR spectrum of 6-(4-(dipropylamino)but-2-ynyloxy)-2*H*-chromen-2-one (**7**) ($CDCl_3$, 300 MHz).
4 Figure S4. ^{13}C NMR spectrum of 6-(4-(dipropylamino)but-2-ynyloxy)-2*H*-chromen-2-one (**7**) ($CDCl_3$,125 MHz).
5 Figure S5. ^{1}H NMR spectrum of 6-(4-(azepan-1-yl)but-2-ynyloxy)-2*H*-chromen-2-one (**9**) ($CDCl_3$, 300 MHz).
6 Figure S6. ^{13}C NMR spectrum of 6-(4-(azepan-1-yl)but-2-ynyloxy)-2*H*-chromen-2-one (**9**) ($CDCl_3$,125 MHz).
7 Figure S7. ^{1}H NMR spectrum of 6-(4-(4-methylpiperazin-1-yl)but-2-ynyloxy)-2*H*-chromen-2-one (**14**) ($CDCl_3$, 300 MHz).
8 Figure S8. ^{13}C NMR spectrum of 6-(4-(4-methylpiperazin-1-yl)but-2-ynyloxy)-2*H*-chromen-2-one (**14**) ($CDCl_3$,125 MHz).
9 Figure S9. ^{1}H NMR spectrum of 6-(4-(4-phenylpiperazin-1-yl)but-2-ynyloxy)-2*H*-chromen-2-one (**15**) ($CDCl_3$, 300 MHz).
10 Figure S10. ^{13}C NMR spectrum of 6-(4-(4-phenylpiperazin-1-yl)but-2-ynyloxy)-2*H*-chromen-2-one (**15**) ($CDCl_3$,125 MHz).
11 Figure S11. ^{1}H NMR spectrum of 6-(4-(4-(furan-2-carbonyl)piperazin-1-yl)but-2-ynyloxy)-2*H*-chromen-2-one (**16**) ($(CD_3)_2SO$, 300 MHz).
12 Figure S12. ^{13}C NMR spectrum of 6-(4-(4-(furan-2-carnonyl)piperazin-1-yl)but-2-ynyloxy)-2*H*-chromen-2-one (**16**) ($CDCl_3$,125 MHz).
13 Figure S13. ^{1}H NMR spectrum of 6-(4-(4-(tetrahydrofuran-2-carbonyl)piperazin-1-yl)but-2-ynyloxy)-2*H*-chromen-2-one (**17**) $CDCl_3$, 300 MHz).
14 Figure S14. ^{13}C NMR spectrum of 6-(4-(4-(tetrahydrofuran-2-carbonyl)piperazin-1-yl)but-2-ynyloxy)-2*H*-chromen-2-one (**16**) ($CDCl_3$,125 MHz).
15 Figure S15. ^{1}H NMR spectrum of 7-(4-(diisopropylamino)but-2-ynyloxy)-2*H*-chromen-2-one (**18**) ($CDCl_3$, 300 MHz).
16 Figure S16. ^{13}C NMR spectrum of 7-(4-(diisopropylamino)but-2-ynyloxy)-2*H*-chromen-2-one (**18**) ($CDCl_3$, 125 MHz).
17 Figure S17. ^{1}H NMR spectrum of 7-(4-(diisopropylamino)but-2-ynyloxy)-4-methyl-2*H*-chromen-2-one (**19**) ($CDCl_3$, 300 MHz).
18 Figure S18. ^{13}C NMR spectrum of 7-(4-(diisopropylamino)but-2-ynyloxy)-4-methyl-2*H*-chromen-2-one (**19**) ($CDCl_3$, 125 MHz).
19 Figure S19. ^{1}H NMR spectrum of 7-(4-(dipropylamino)but-2-ynyloxy)-2*H*-chromen-2-one (**20**) ($CDCl_3$, 300 MHz).
20 Figure S20. ^{13}C NMR spectrum of 7-(4-(dipropylamino)but-2-ynyloxy)-2*H*-chromen-2-one (**20**) ($CDCl_3$, 125 MHz).
21 Figure S21. ^{1}H NMR spectrum of 7-(4-(dipropylamino)but-2-ynyloxy)-4-methyl-2*H*-chromen-2-one (**21**) ($CDCl_3$, 300 MHz).
22 Figure S22. ^{13}C NMR spectrum of 7-(4-(dipropylamino)but-2-ynyloxy)-4-methyl-2*H*-chromen-2-one (**21**) ($CDCl_3$, 125 MHz).
23 Figure S23. ^{1}H NMR spectrum of 7-(4-(dibutylamino)but-2-ynyloxy)-2*H*-chromen-2-one (**23**) ($CDCl_3$, 300 MHz).
24 Figure S24. ^{13}C NMR spectrum of 7-(4-(dibutylamino)but-2-ynyloxy)-2*H*-chromen-2-one (**23**) ($CDCl_3$, 125 MHz).

Figure S25. ^{1}H NMR spectrum of 7-(4-(dibutylamino)but-2-ynyloxy)-4-methyl-2*H*-chromen-2-one (**24**) ($CDCl_3$, 300 MHz).
Figure S26. ^{13}C NMR spectrum of 7-(4-(dibutylamino)but-2-ynyloxy)-4-methyl-2*H*-chromen-2-one (**24**) ($CDCl_3$, 125 MHz).
Figure S27. ^{1}H NMR spectrum of 7-(4-(azepan-1-yl)but-2-ynyloxy)-2*H*-chromen-2-one (**25**) ($CDCl_3$, 300 MHz).
Figure S28. ^{13}C NMR spectrum of 7-(4-(azepan-1-yl)but-2-ynyloxy)-2*H*-chromen-2-one (**25**) ($CDCl_3$,125 MHz).
Figure S29. ^{1}H NMR spectrum of 7-(4-(azepan-1-yl)but-2-ynyloxy)-4-methyl-2*H*-chromen-2-one (**26**) ($CDCl_3$, 300 MHz).
Figure S30. ^{13}C NMR spectrum of 7-(4-(azepan-1-yl)but-2-ynyloxy)-4-methyl-2*H*-chromen-2-one (**26**) ($CDCl_3$,125 MHz).
Figure S31. ^{1}H NMR spectrum of 7-(4-(azocan-1-yl)but-2-ynyloxy)-2*H*-chromen-2-one (**28**) ($CDCl_3$, 300 MHz).
Figure S32. ^{13}C NMR spectrum of 7-(4-(azocan-1-yl)but-2-ynyloxy)-2*H*-chromen-2-one (**28**) ($CDCl_3$,125 MHz).
Figure S33. ^{1}H NMR spectrum of 7-(4-(azocan-1-yl)but-2-ynyloxy)-4-methyl-2*H*-chromen-2-one (**29**) ($CDCl_3$, 300 MHz).
Figure S34. ^{13}C NMR spectrum of 7-(4-(azocan-1-yl)but-2-ynyloxy)-4-methyl-2*H*-chromen-2-one (**29**) ($CDCl_3$,125 MHz).
Figure S35. ^{1}H NMR spectrum of 7-(4-(4-methylpiperazin-1-yl)but-2-ynyloxy)-2*H*-chromen-2-one (**30**) ($CDCl_3$, 300 MHz).
Figure S36. ^{13}C NMR spectrum of 7-(4-(4-methylpiperazin-1-yl)but-2-ynyloxy)-2*H*-chromen-2-one (**30**) ($CDCl_3$, 125 MHz).
Figure S37. ^{1}H NMR spectrum of 7-(4-(4-methylpiperazin-1-yl)but-2-ynyloxy)-4-methyl-2*H*-chromen-2-one (**31**) ($CDCl_3$, 300 MHz).
Figure S38. ^{13}C NMR spectrum of 7-(4-(4-methylpiperazin-1-yl)but-2-ynyloxy)-4-methyl-2*H*-chromen-2-one (**31**) ($CDCl_3$, 125 MHz).
Figure S39. ^{1}H NMR spectrum of 7-(4-(4-phenylpiperazin-1-yl)but-2-ynyloxy)-2*H*-chromen-2-one (**32**) ($CDCl_3$, 300 MHz).
Figure S40. ^{13}C NMR spectrum of 7-(4-(4-phenylpiperazin-1-yl)but-2-ynyloxy)-2*H*-chromen-2-one (**32**) ($CDCl_3$, 125 MHz).
Figure S41. ^{1}H NMR spectrum of 7-(4-(4-phenylpiperazin-1-yl)but-2-ynyloxy)-4-methyl-2*H*-chromen-2-one (**33**) ($CDCl_3$, 300 MHz).
Figure S42. ^{13}C NMR spectrum of 7-(4-(4-phenylpiperazin-1-yl)but-2-ynyloxy)-4-methyl-2*H*-chromen-2-one (**33**) ($CDCl_3$, 125 MHz).
Figure S43. ^{1}H NMR spectrum of 7-(4-(4-(4-hydroxyphenyl)piperazin-1-yl)but-2-ynyloxy)-2*H*-chromen-2-one (**35**) ($(CD_3)_2SO$, 300 MHz).
Figure S44. ^{13}C NMR spectrum of 7-(4-(4-(4-hydroxyphenyl)piperazin-1-yl)but-2-ynyloxy)-2*H*-chromen-2-one (**35**) ($(CD_3)_2SO$, 125 MHz).
Figure S45. ^{1}H NMR spectrum of 7-(4-(4-(4-hydroxyphenyl)piperazin-1-yl)but-2-ynyloxy)-4-methyl-2*H*-chromen-2-one (**36**) ($CDCl_3$, 300 MHz).
Figure S46. ^{13}C NMR spectrum of 7-(4-(4-(4-hydroxyphenyl)piperazin-1-yl)but-2-ynyloxy)-4-methyl-2*H*-chromen-2-one (**36**) ($CDCl_3$, 125 MHz).
Figure S47. ^{1}H NMR spectrum of 7-(4-(4-(furan-2-carbonyl)piperazin-1-yl)but-2-ynyloxy)-2*H*-chromen-2-one (**37**) (($CDCl_3$, 300 MHz).
Figure S48. ^{13}C NMR spectrum of 7-(4-(4-(furan-2-carbonyl)piperazin-1-yl)but-2-ynyloxy)-2*H*-chromen-2-one (**37**) (($CDCl_3$, 125 MHz).
Figure S49. ^{1}H NMR spectrum of 7-(4-(4-(furan-2-carbonyl)piperazin-1-yl)but-2-ynyloxy)-4-methyl-2*H*-chromen-2-one (**38**) ($CDCl_3$, 300 MHz).
Figure S50. ^{13}C NMR spectrum of 7-(4-(4-(furan-2-carbonyl)piperazin-1-yl)but-2-ynyloxy)-4-methyl-2*H*-chromen-2-one (**38**) ($CDCl_3$, 125 MHz).
Figure S51. ^{1}H NMR spectrum of 7-(4-(4-(tetrahydrofuran-2-carbonyl)piperazin-1-yl)but-2-ynyloxy)-2*H*-chromen-2-one (**39**) ($CDCl_3$, 300 MHz).
Figure S52. ^{13}C NMR spectrum of 7-(4-(4-(tetrahydrofuran-2-carbonyl)piperazin-1-yl)but-2-ynyloxy)-2*H*-chromen-2-one (**39**) ($CDCl_3$, 125 MHz).
Figure S53. ^{1}H NMR spectrum of 7-(4-(4-(benzo[*d*][1,3]dioxol-5-ylmethyl)piperazin-1-yl)but-2-ynyloxy)-2*H*-chromen-2-one (**41**) ($CDCl_3$, 300 MHz).
Figure S54. ^{13}C NMR spectrum of 7-(4-(4-(benzo[*d*][1,3]dioxol-5-ylmethyl)piperazin-1-yl)but-2-ynyloxy)-2*H*-chromen-2-one (**41**) ($CDCl_3$, 125 MHz).
Figure S55. ^{1}H NMR spectrum of 7-(4-(4-(benzo[*d*][1,3]dioxol-5-ylmethyl)piperazin-1-yl)but-2-ynyloxy)-4-methyl-2*H*-chromen-2-one (**42**) ($CDCl_3$, 300 MHz).
Figure S56. ^{13}C NMR spectrum of 7-(4-(4-(benzo[*d*][1,3]dioxol-5-ylmethyl)piperazin-1-yl)but-2-ynyloxy)-4-methyl-2*H*-chromen-2-one (**42**) ($CDCl_3$, 125 MHz).
Figure S57. ^{1}H NMR spectrum of 7-(4-(3,4-dihydroisoquinolin-2(1*H*)-yl)but-2-ynyloxy)-2*H*-chromen-2-one (**44**) ($CDCl_3$, 300 MHz).
Figure S58. ^{13}C NMR spectrum of 7-(4-(3,4-dihydroisoquinolin-2(1*H*)-yl)but-2-ynyloxy)-2*H*-chromen-2-one (**44**) $CDCl_3$, 125 MHz).
Figure S59. ^{1}H NMR spectrum of 7-(4-(3,4-dihydroisoquinolin-2(1*H*)-yl)but-2-ynyloxy)-4-methyl-2*H*-chromen-2-one (**45**) ($CDCl_3$, 300 MHz).
Figure S60. ^{13}C NMR spectrum of 7-(4-(3,4-dihydroisoquinolin-2(1*H*)-yl)but-2-ynyloxy)-4-methyl-2*H*-chromen-2-one (**45**) $CDCl_3$, 125 MHz).

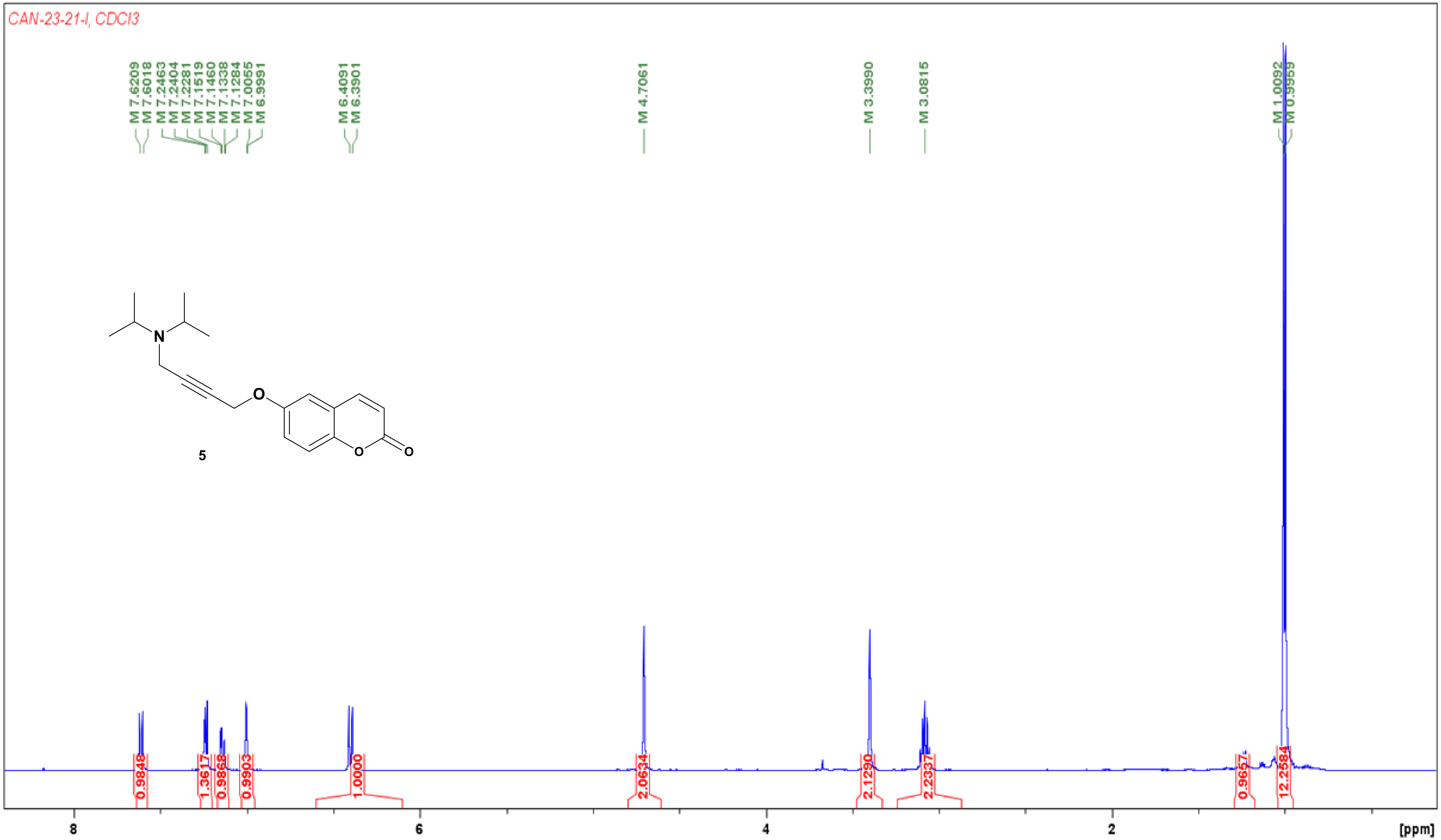

Figure S1. ^{1}H NMR spectrum of 6-(4-(diisopropylamino)but-2-ynyloxy)-2*H*-chromen-2-one (**5**) ($CDCl_3$, 300 MHz).

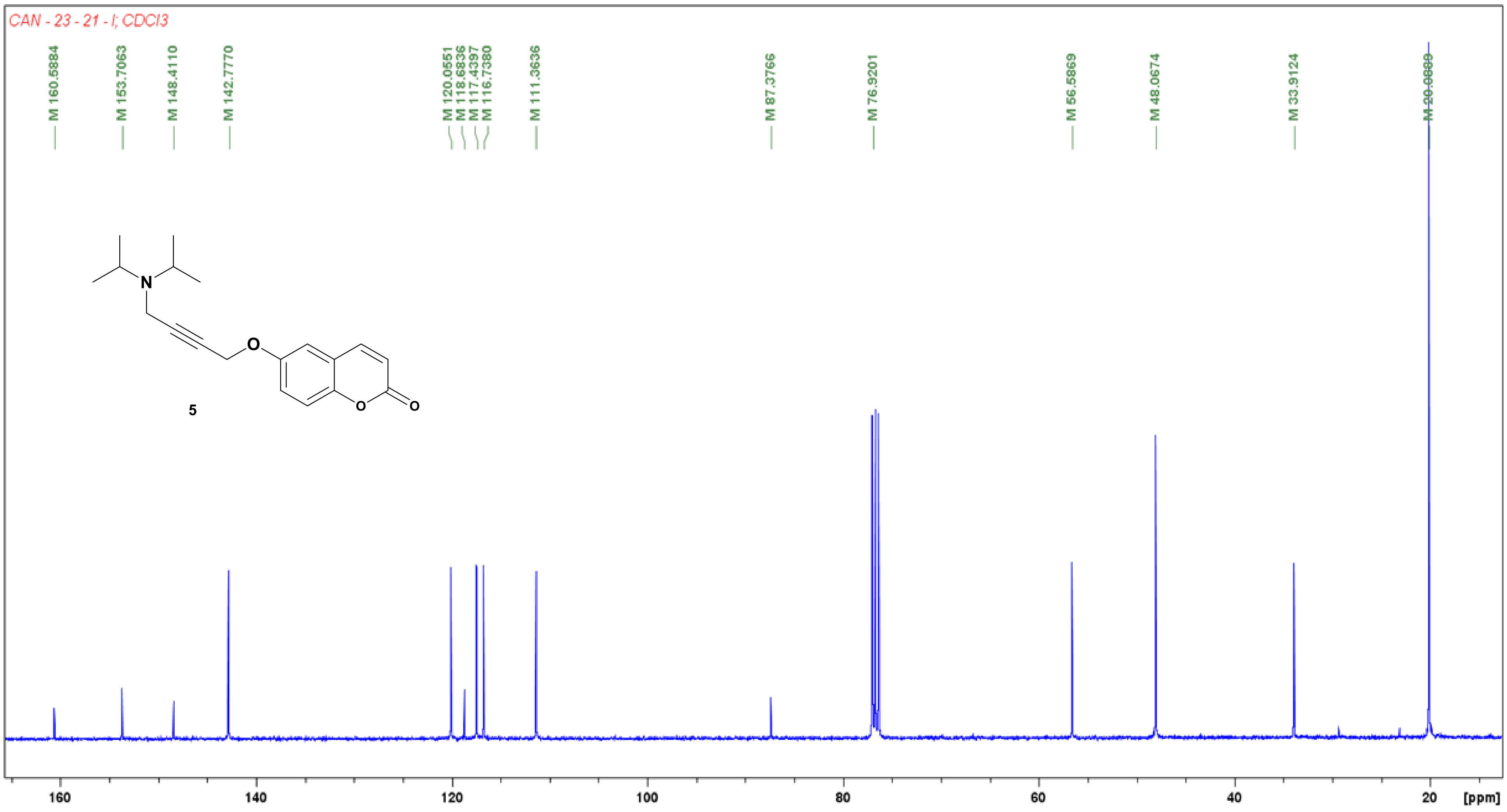

Figure S2. ^{13}C NMR spectrum of 6-(4-(diisopropylamino)but-2-ynyloxy)-2*H*-chromen-2-one (**5**) ($CDCl_3$, 125 MHz).

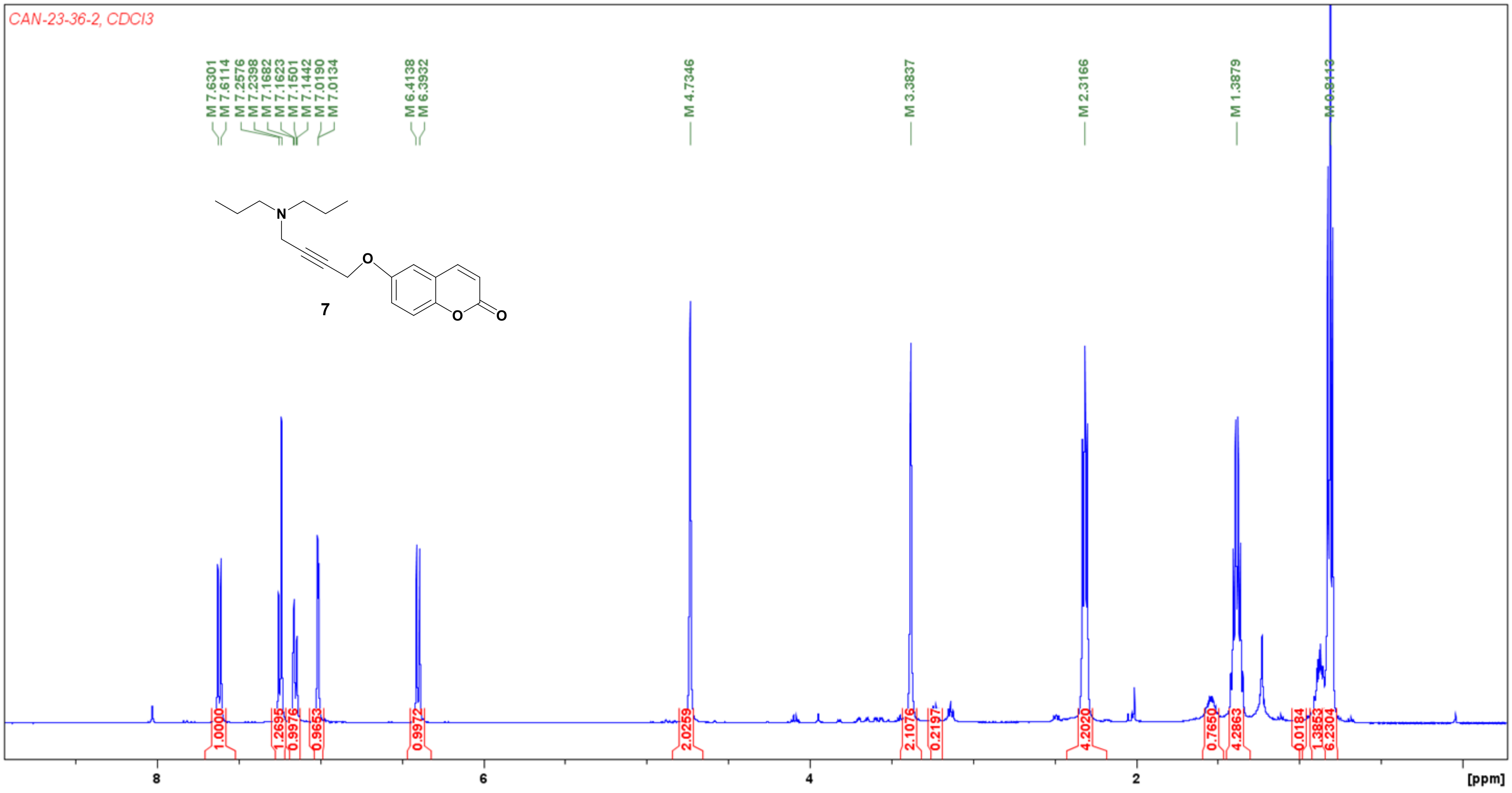

Figure S3. ^{1}H NMR spectrum of 6- (4-(dipropylamino)but-2-ynyloxy)-2*H*-chromen-2-one (**7**) ($CDCl_3$, 300 MHz).

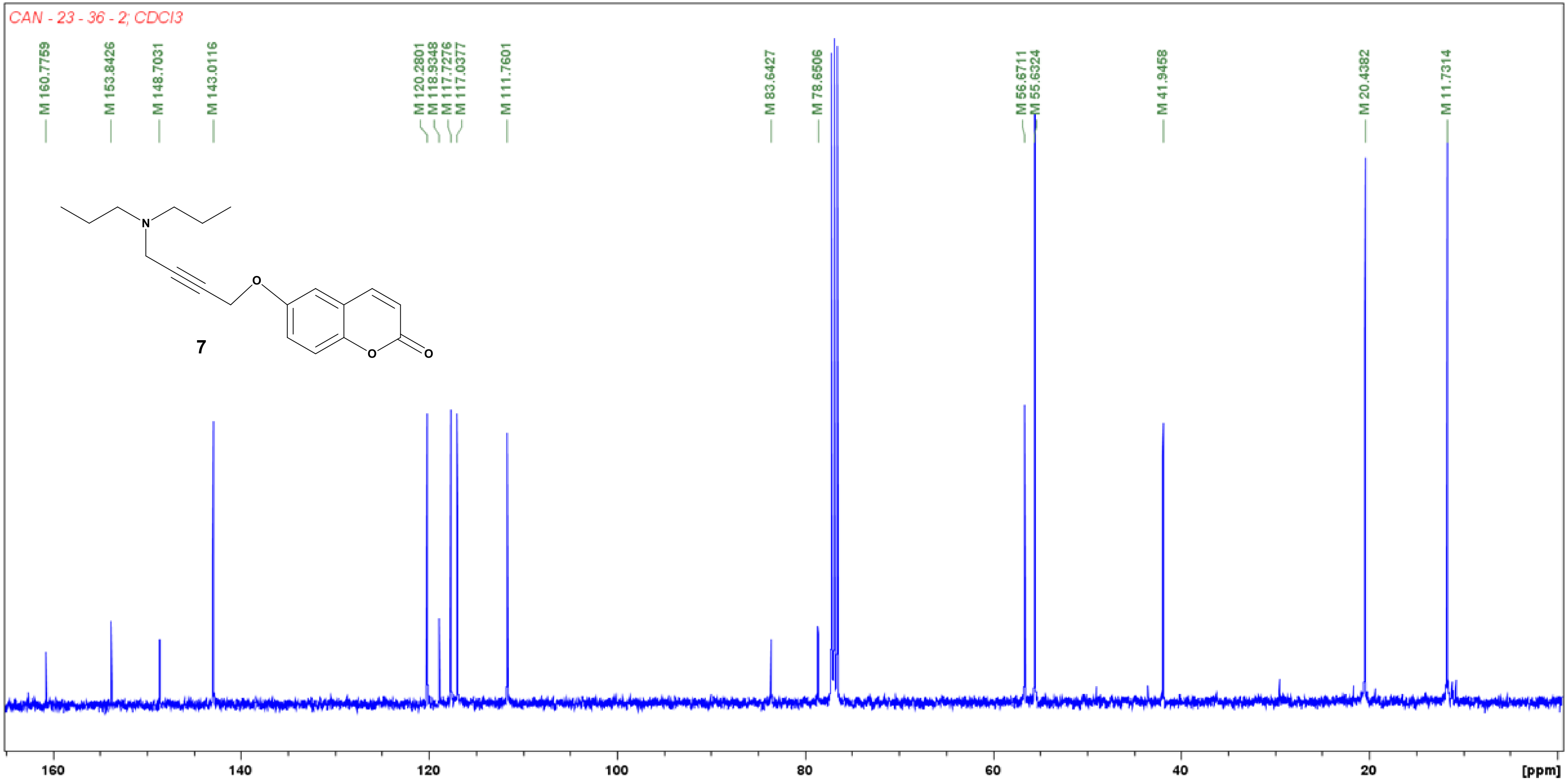

Figure S4. ^{13}C NMR spectrum of 6-(4-(dipropylamino)but-2-ynyloxy)-2*H*-chromen-2-one (**7**) ($CDCl_3$, 125 MHz).

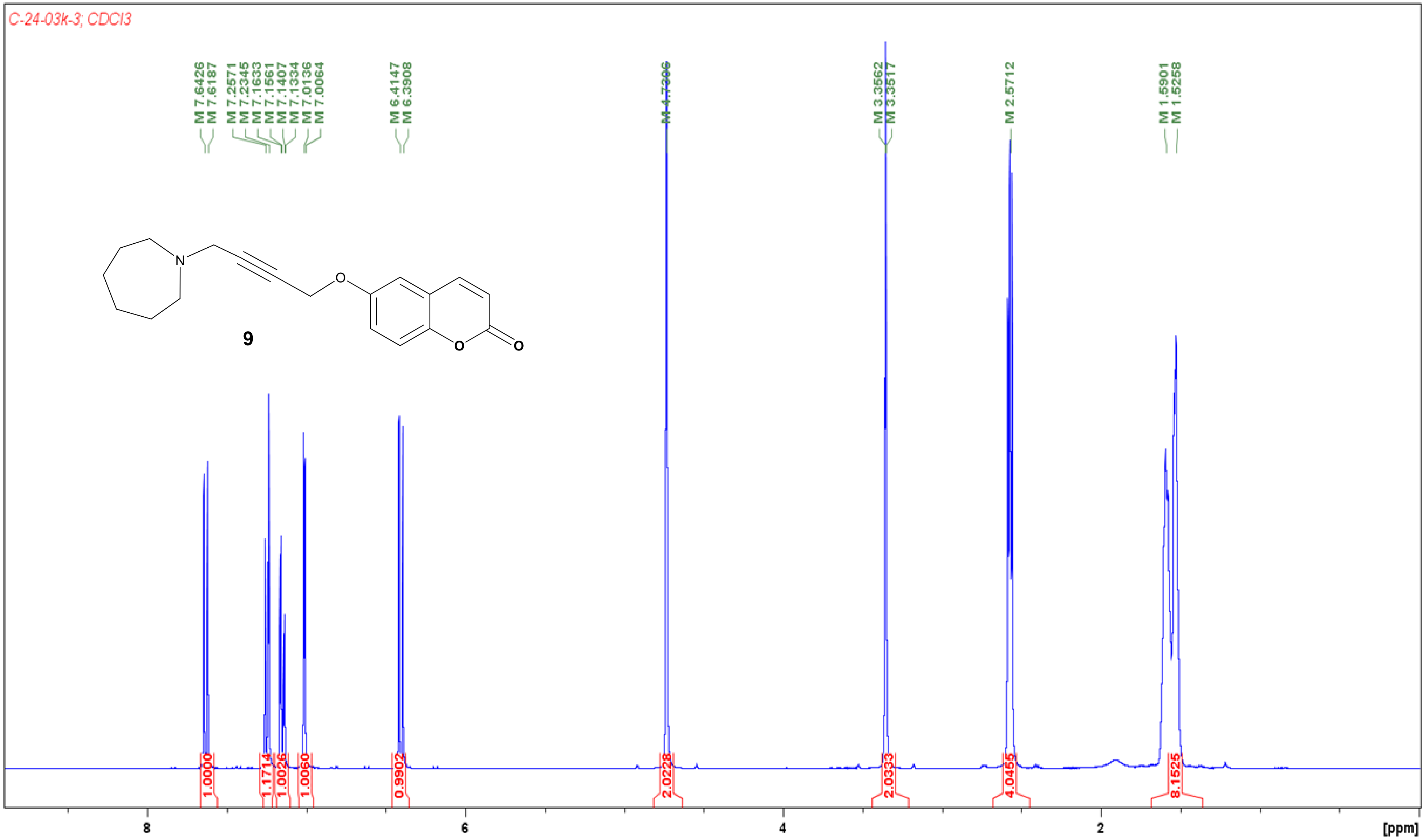

Figure S5. ¹H NMR spectrum of 6-(4-(azepan-1-yl)but-2-ynyloxy)-2*H*-chromen-2-one (**9**) ($CDCl_3$, 300 MHz).

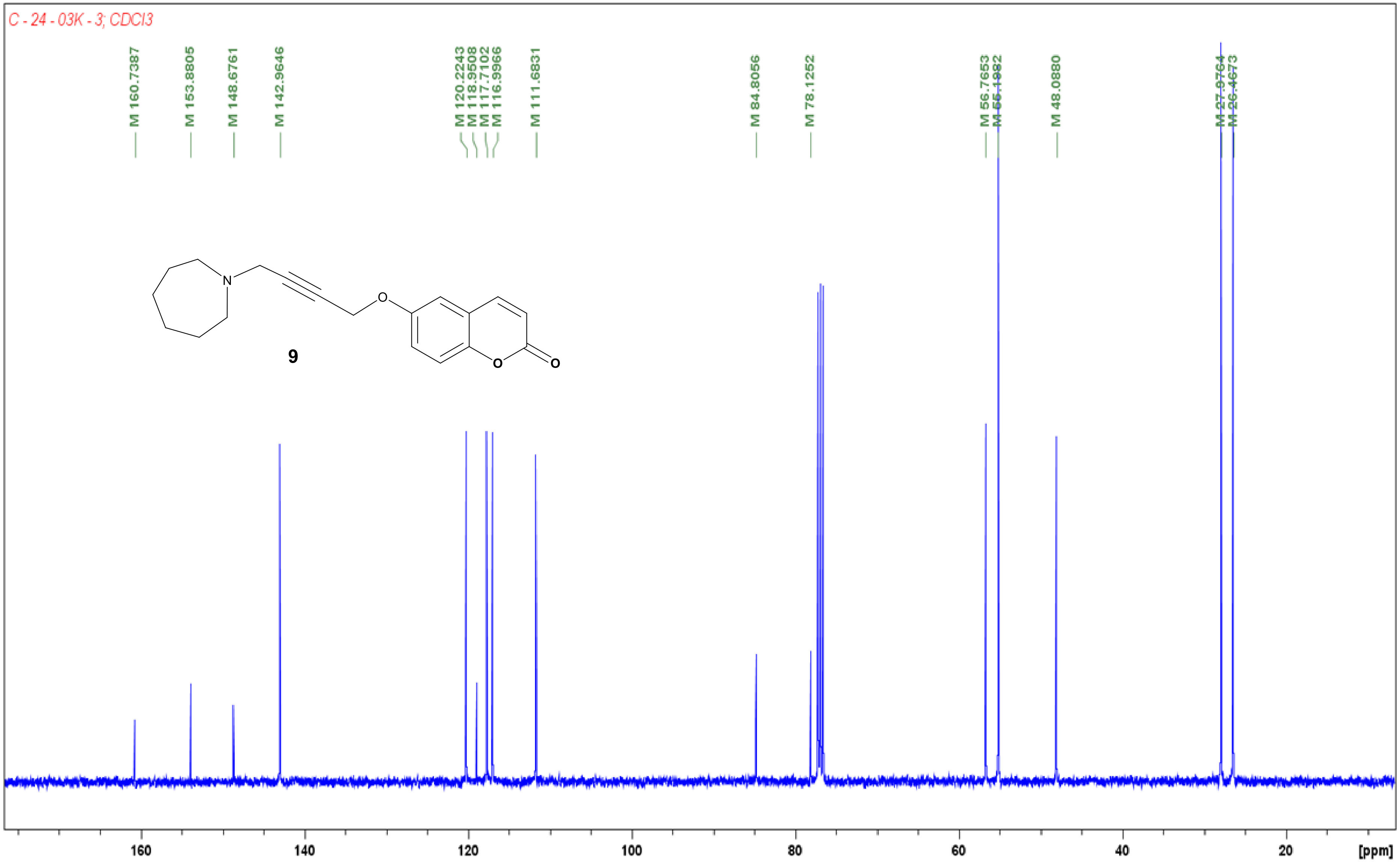

Figure S6. ^{13}C NMR spectrum of 6-(4-(azepan-1-yl)but-2-ynyloxy)-2*H*-chromen-2-one (**9**) ($CDCl_3$,125 MHz).

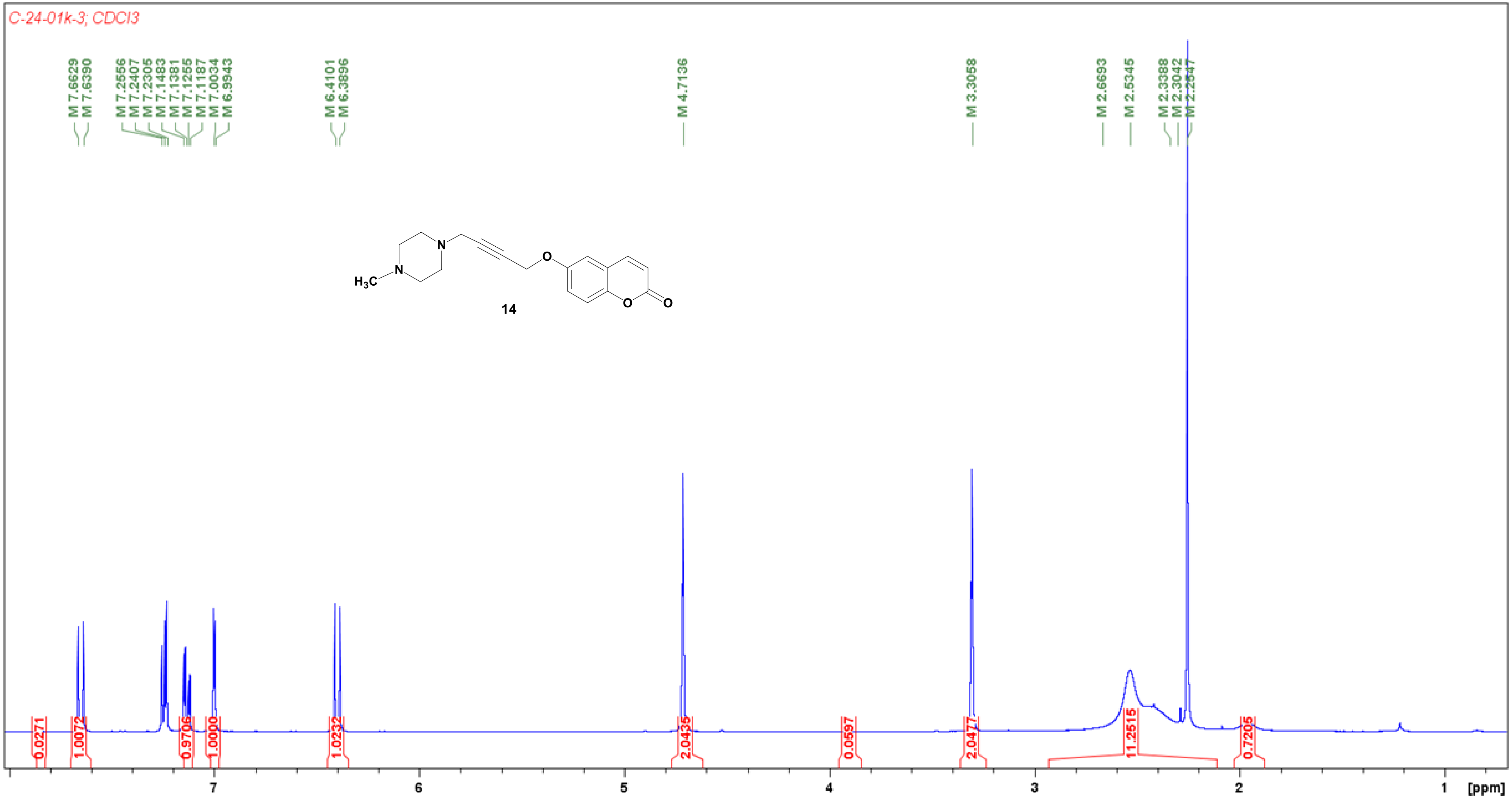

Figure S7. ^{1}H NMR spectrum of 6-(4-(4-methylpiperazin-1-yl)but-2-ynyloxy)-2*H*-chromen-2-one (**14**) ($CDCl_3$, 300 MHz).

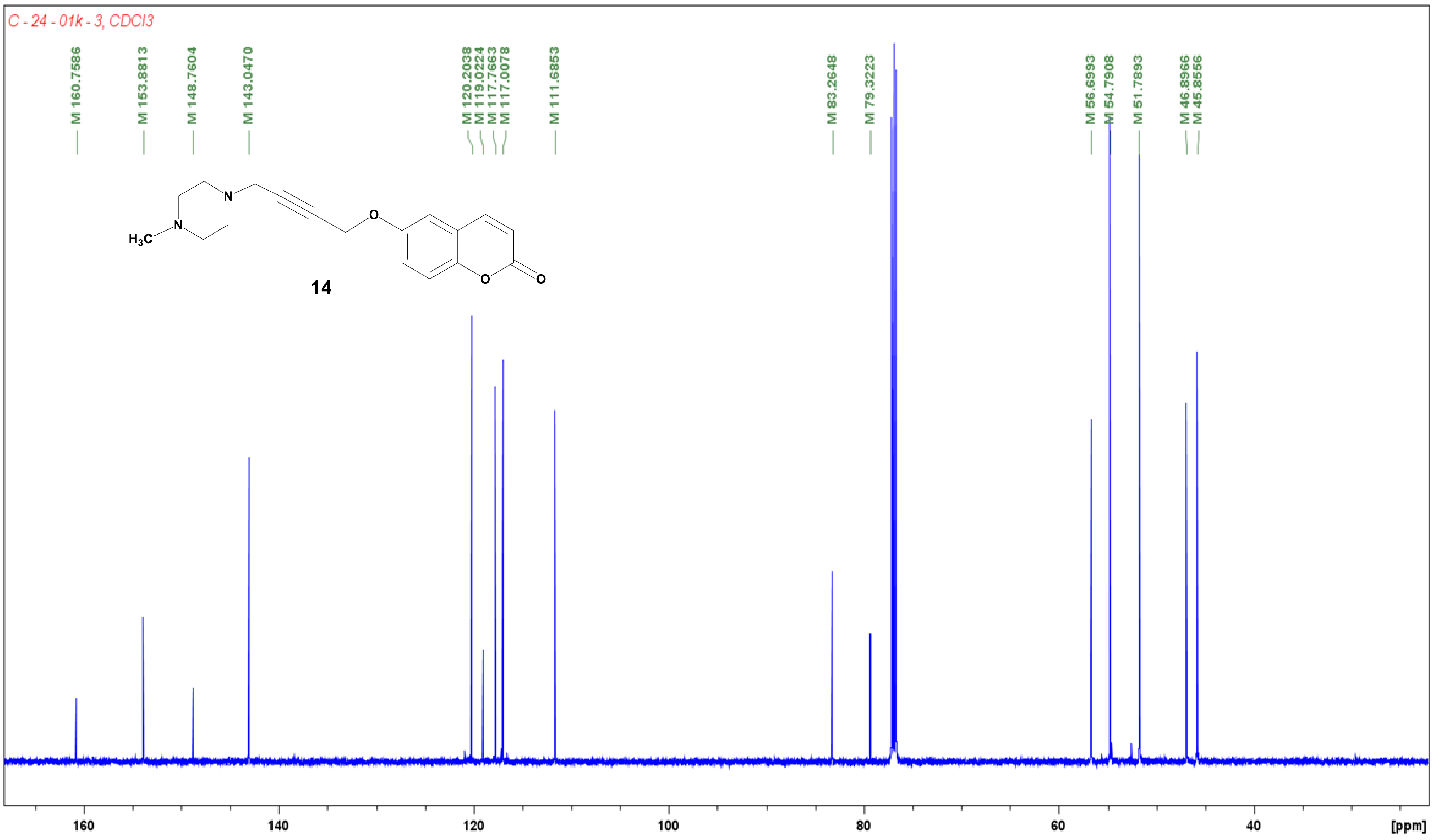

Figure S8. ^{13}C NMR spectrum of 6-(4-(4-methylpiperazin-1-yl)but-2-ynyloxy)-2*H*-chromen-2-one (**14**) $CDCl_3$,125 MHz).

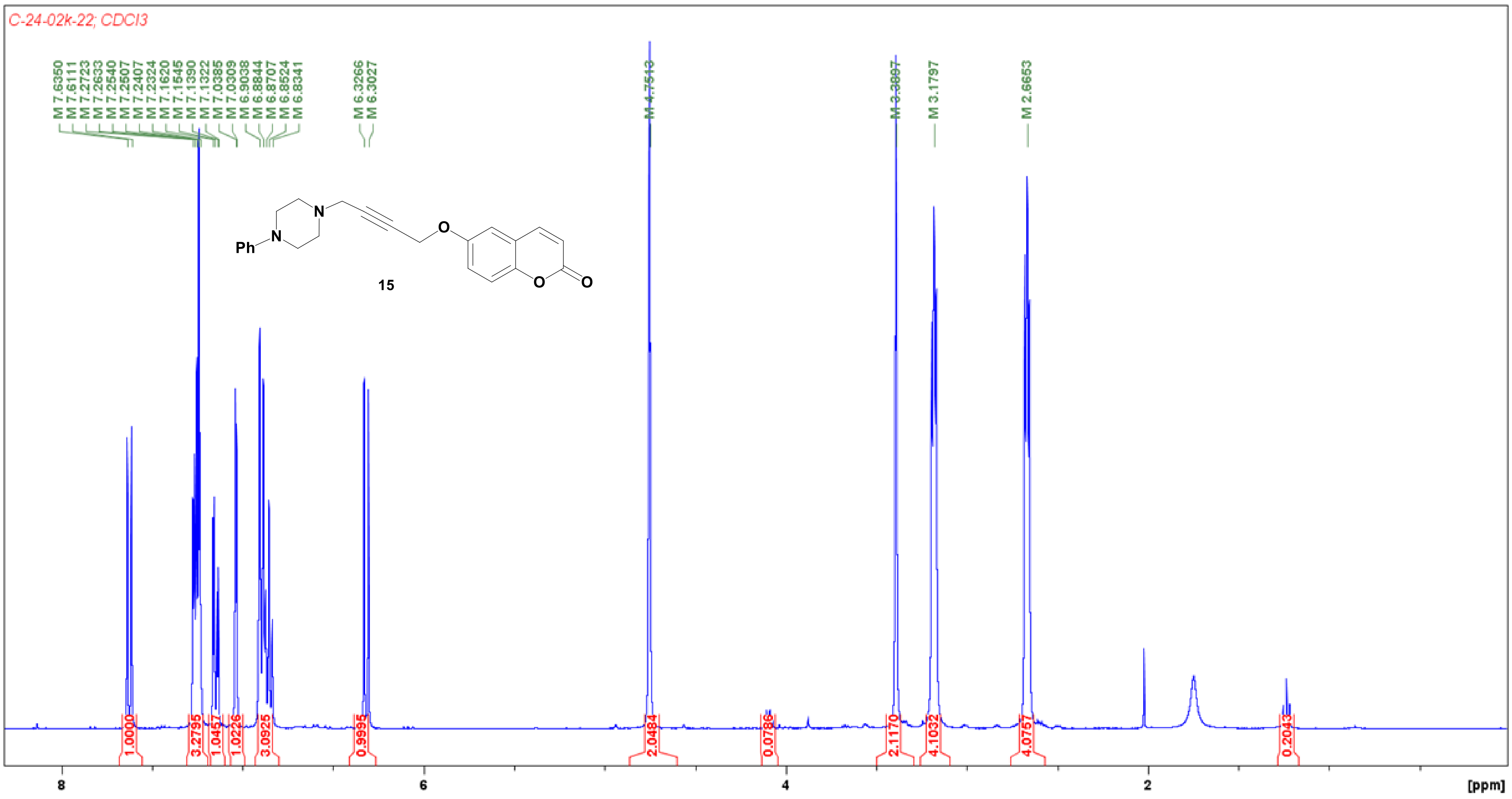

Figure S9. ^{1}H NMR spectrum of 6-(4-(4-phenylpiperazin-1-yl)but-2-ynyloxy)-2*H*-chromen-2-one (**15**) ($CDCl_3$, 300 MHz).

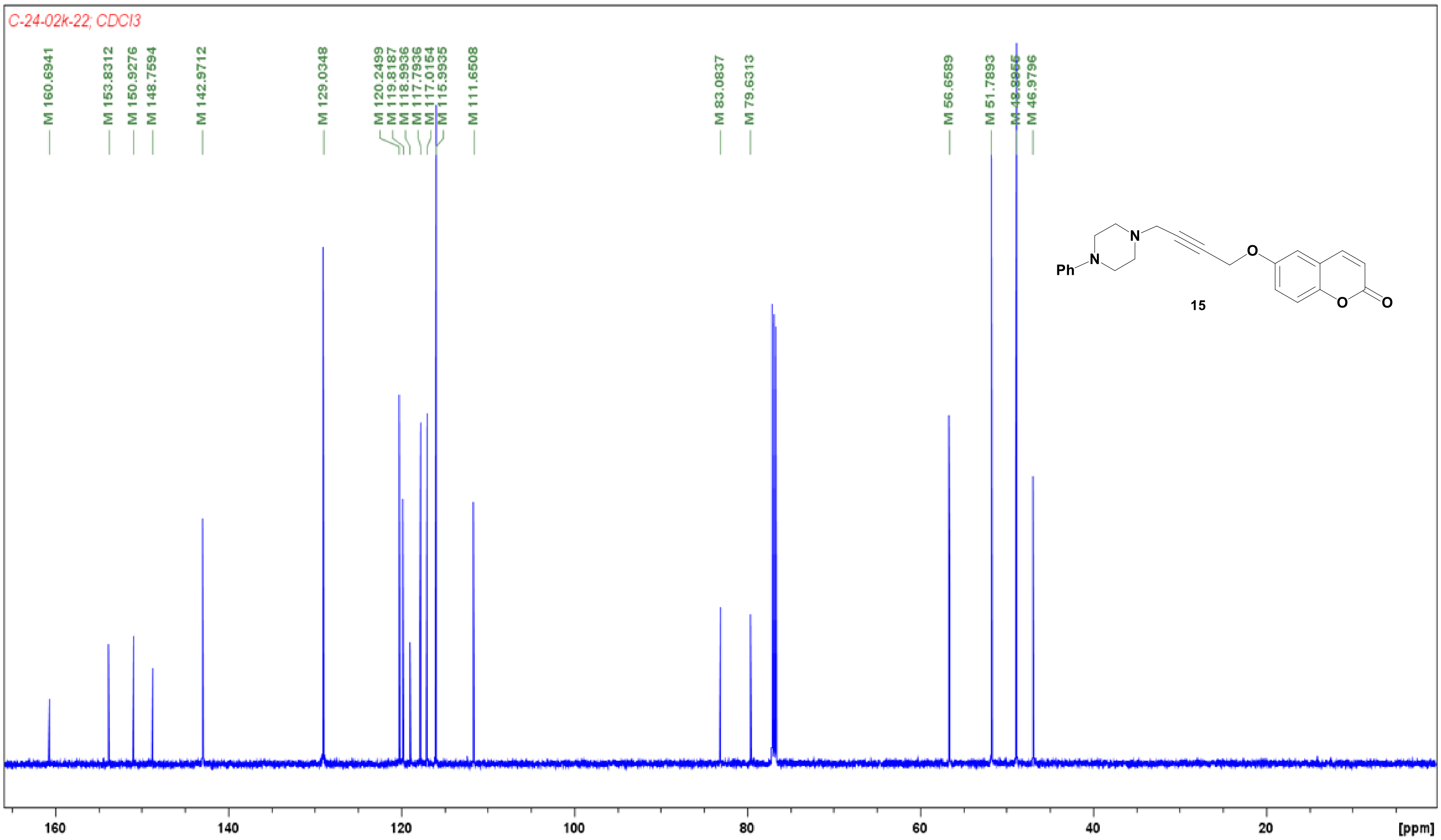

Figure S10. ^{13}C NMR spectrum of 6-(4-(4-phenylpiperazin-1-yl)but-2-ynyloxy)-2*H*-chromen-2-one (**15**) $CDCl_3$,125 MHz).

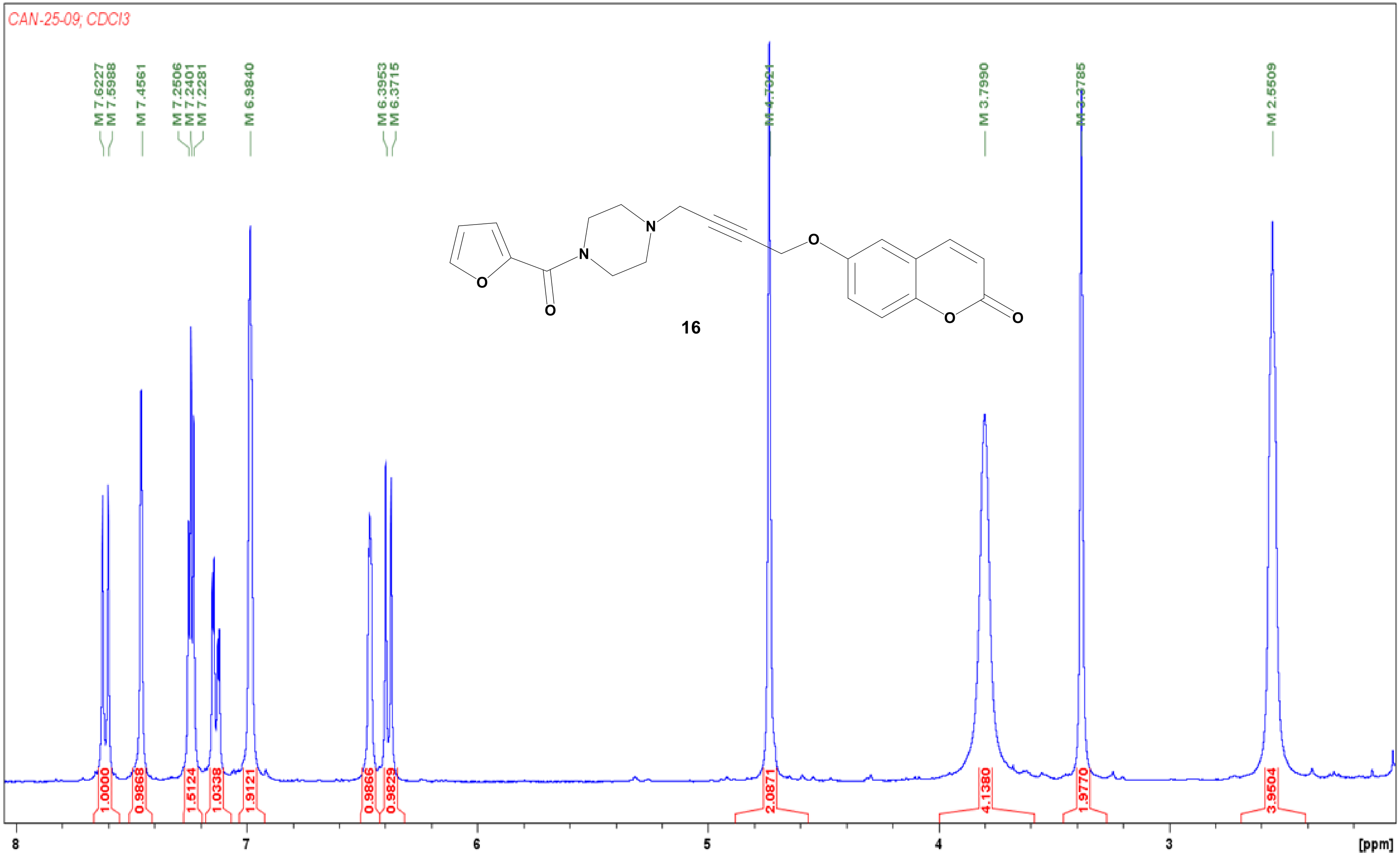

Figure S11. ^{1}H NMR spectrum of 6-(4-(4-(furan-2-carbonyl)piperazin-1-yl)but-2-ynyloxy)-2*H*-chromen-2-one (**16**) ($CDCl_3$, 300 MHz).

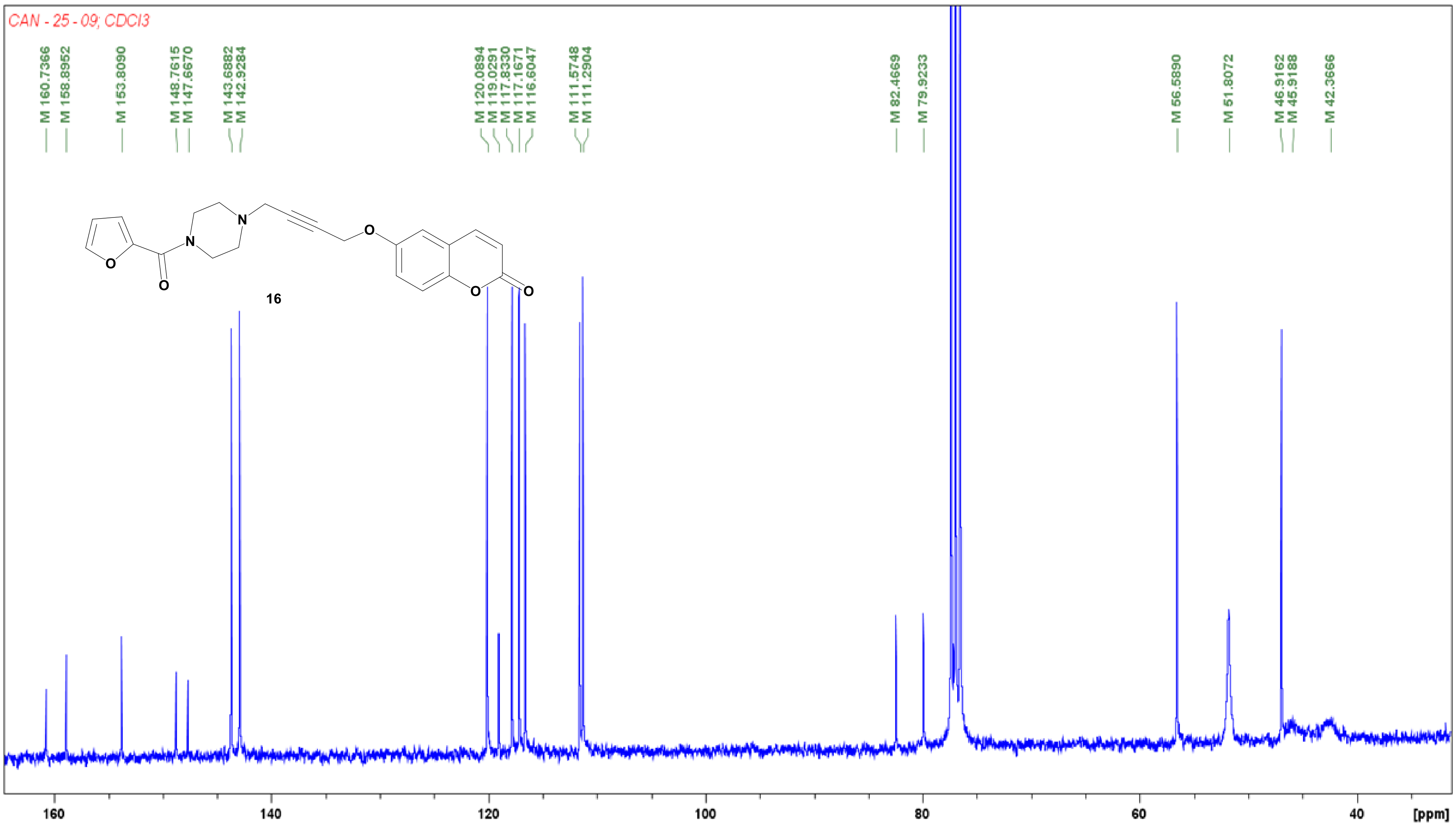

Figure S12. ^{13}C NMR spectrum of 6-(4-(4-(furan-2-carbonyl)piperazin-1-yl)but-2-ynyloxy)-2*H*-chromen-2-one (**16**) ($CDCl_3$,125 MHz).

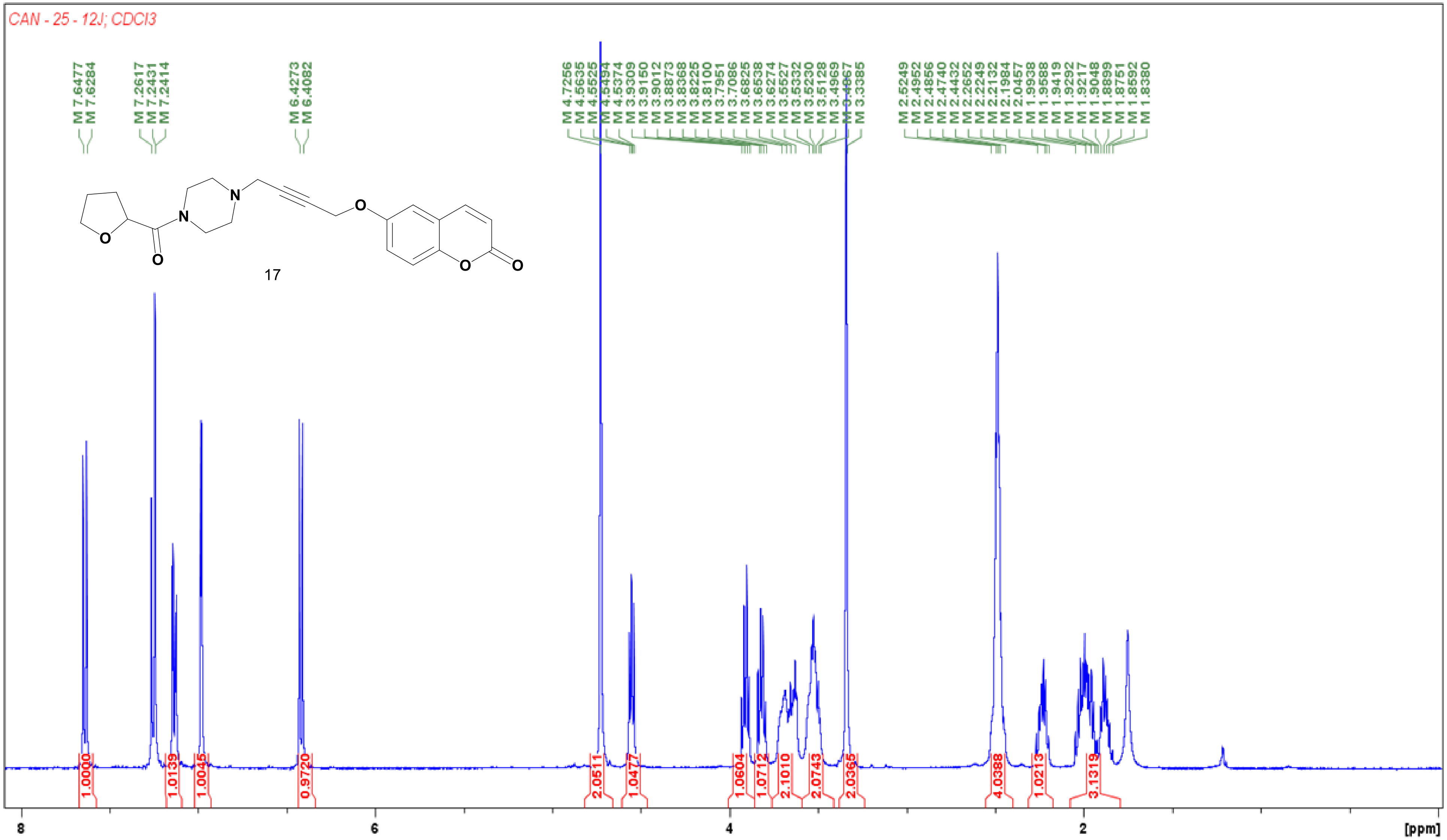

Figure S13. ¹H NMR spectrum of 6-(4-(4-(tetrahydrofuran-2-carbonyl)piperazin-1-yl)but-2-ynyloxy)-2*H*-chromen-2-one (**17**) $CDCl_3$, 300 MHz).

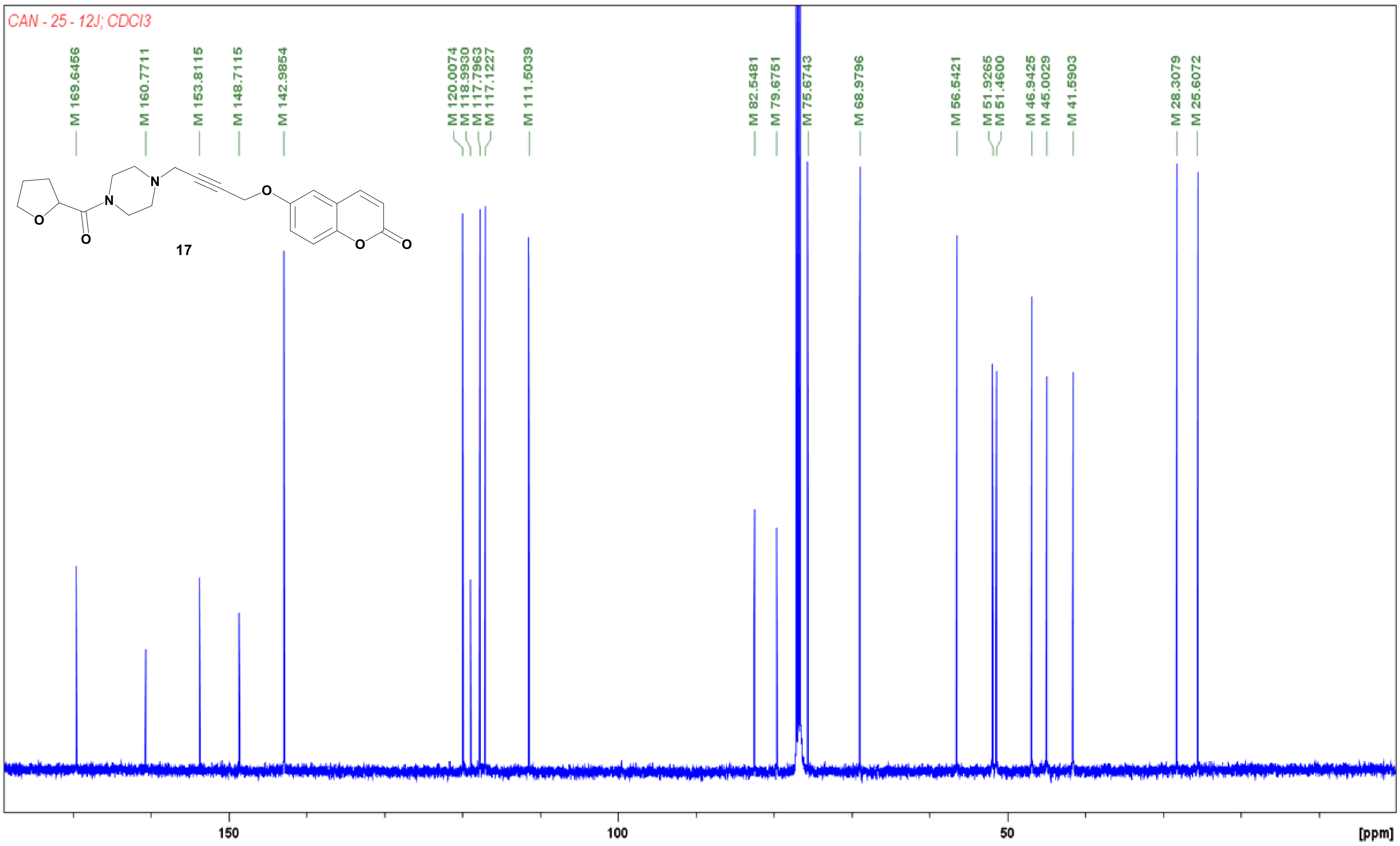

Figure S14. ^{13}C NMR spectrum of 6-(4-(4-(tetrahydrofuran-2-carbonyl)piperazin-1-yl)but-2-ynyloxy)-2*H*-chromen-2-one (**16**) ($CDCl_3$,125 MHz).

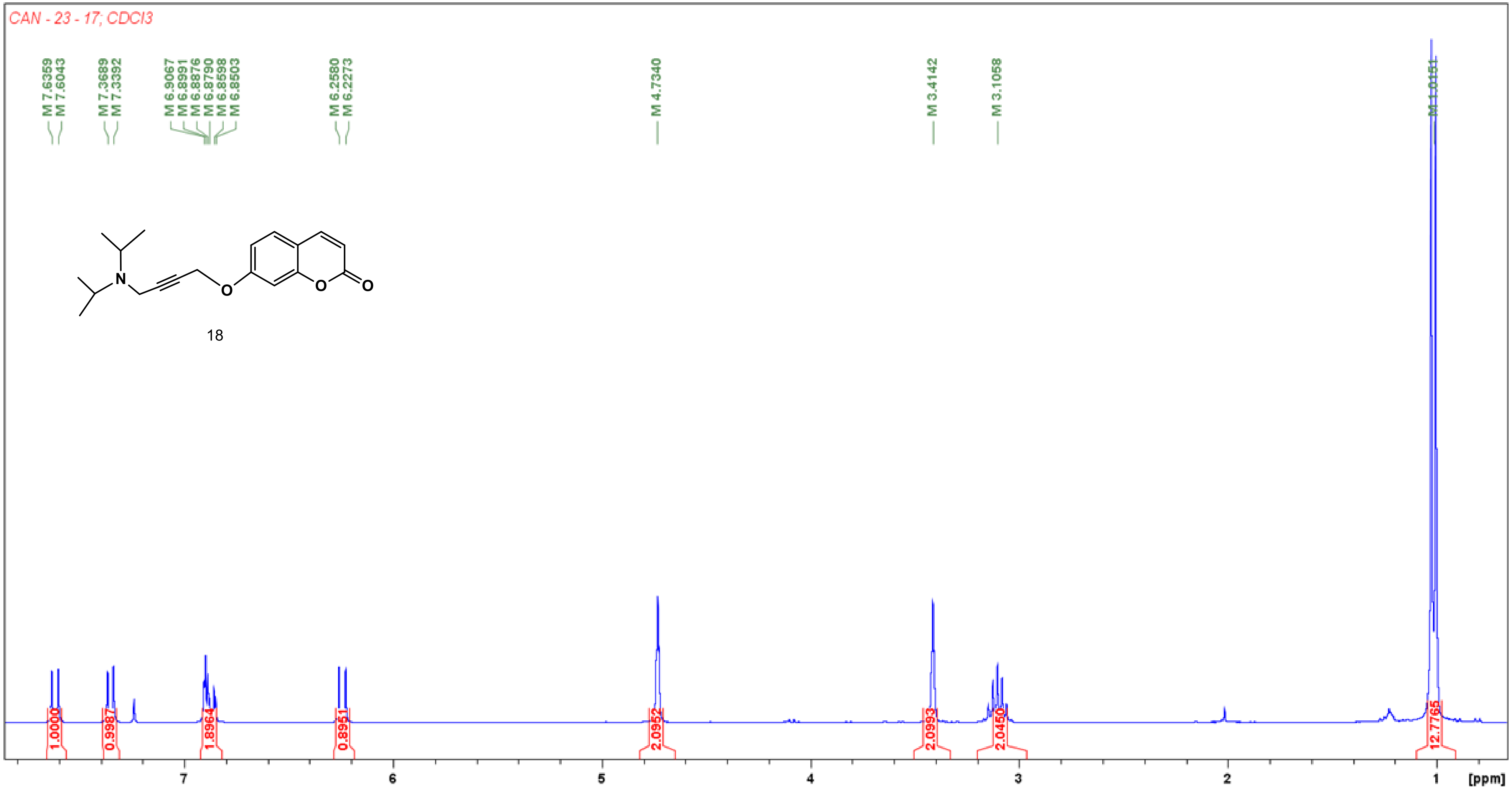

Figure S15. ¹H NMR spectrum of 7-(4-(diisopropylamino)but-2-ynyloxy)-2*H*-chromen-2-one (**18**) ($CDCl_3$, 300 MHz).

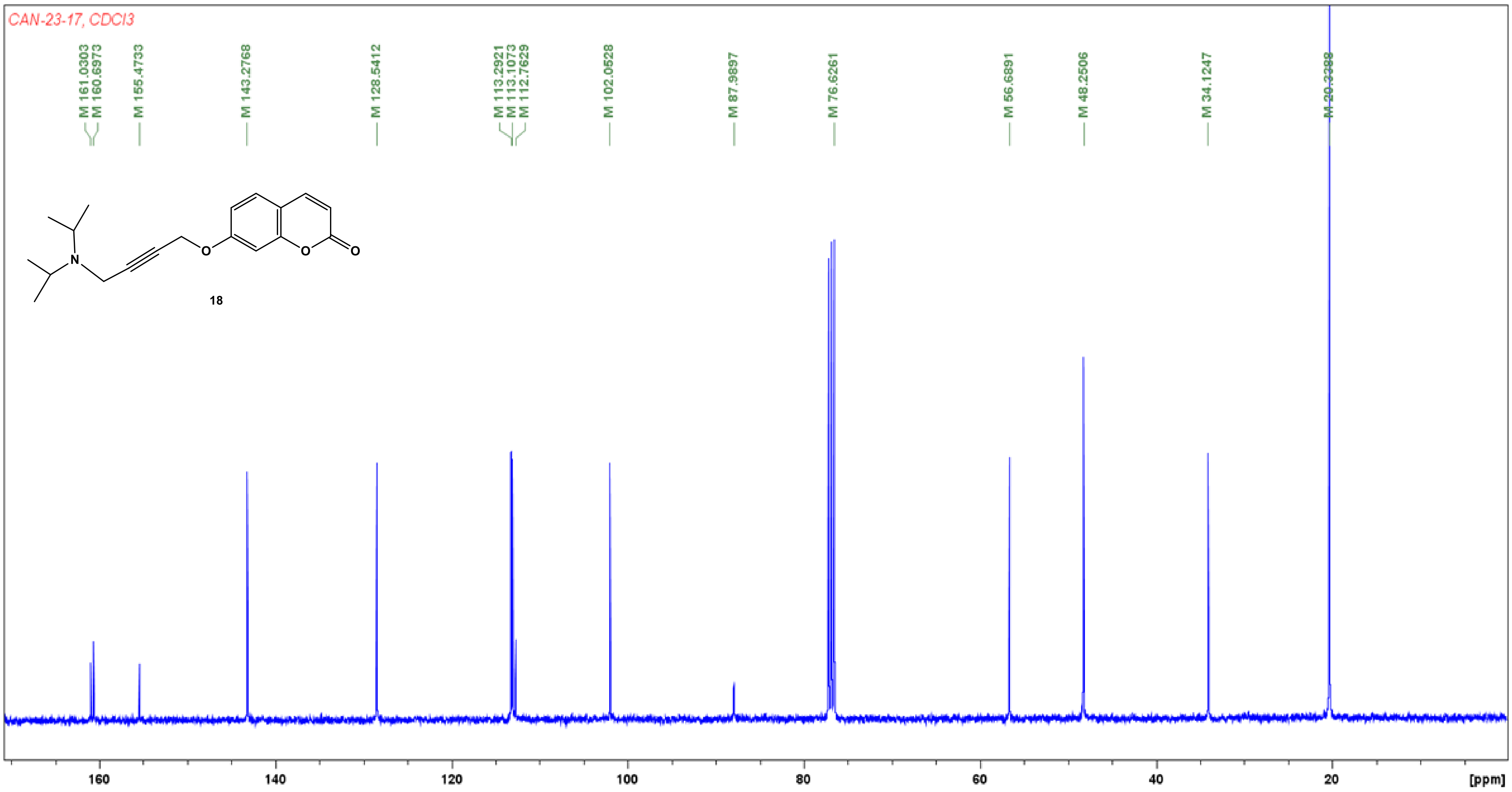

Figure S16. ^{13}C NMR spectrum of 7-(4-(diisopropylamino)but-2-ynyloxy)-2*H*-chromen-2-one (**18**) ($CDCl_3$, 125 MHz).

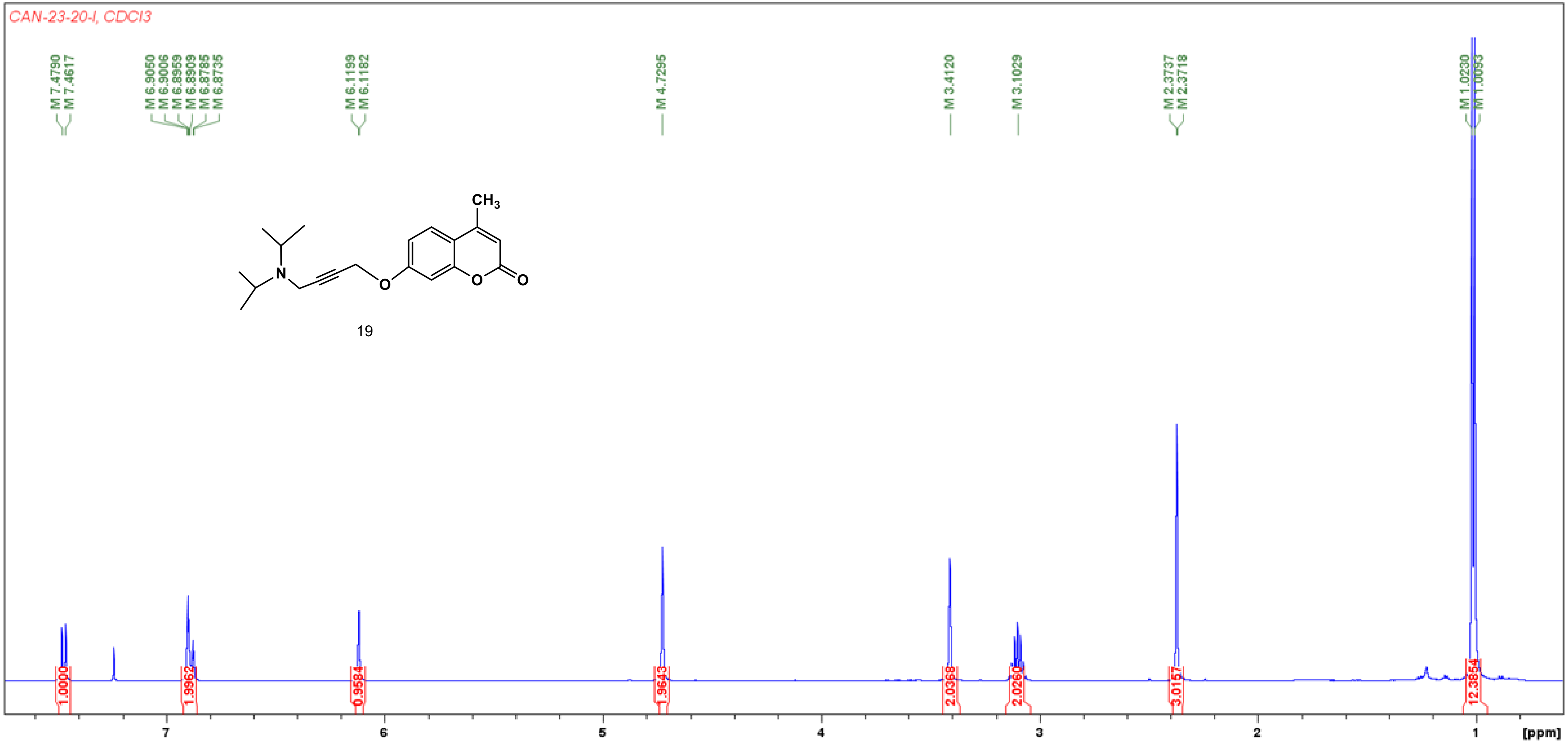

Figure S17. ^{1}H NMR spectrum of 7-(4-(diisopropylamino)but-2-ynyloxy)-4-methyl-2*H*-chromen-2-one (**19**) ($CDCl_3$, 300 MHz).

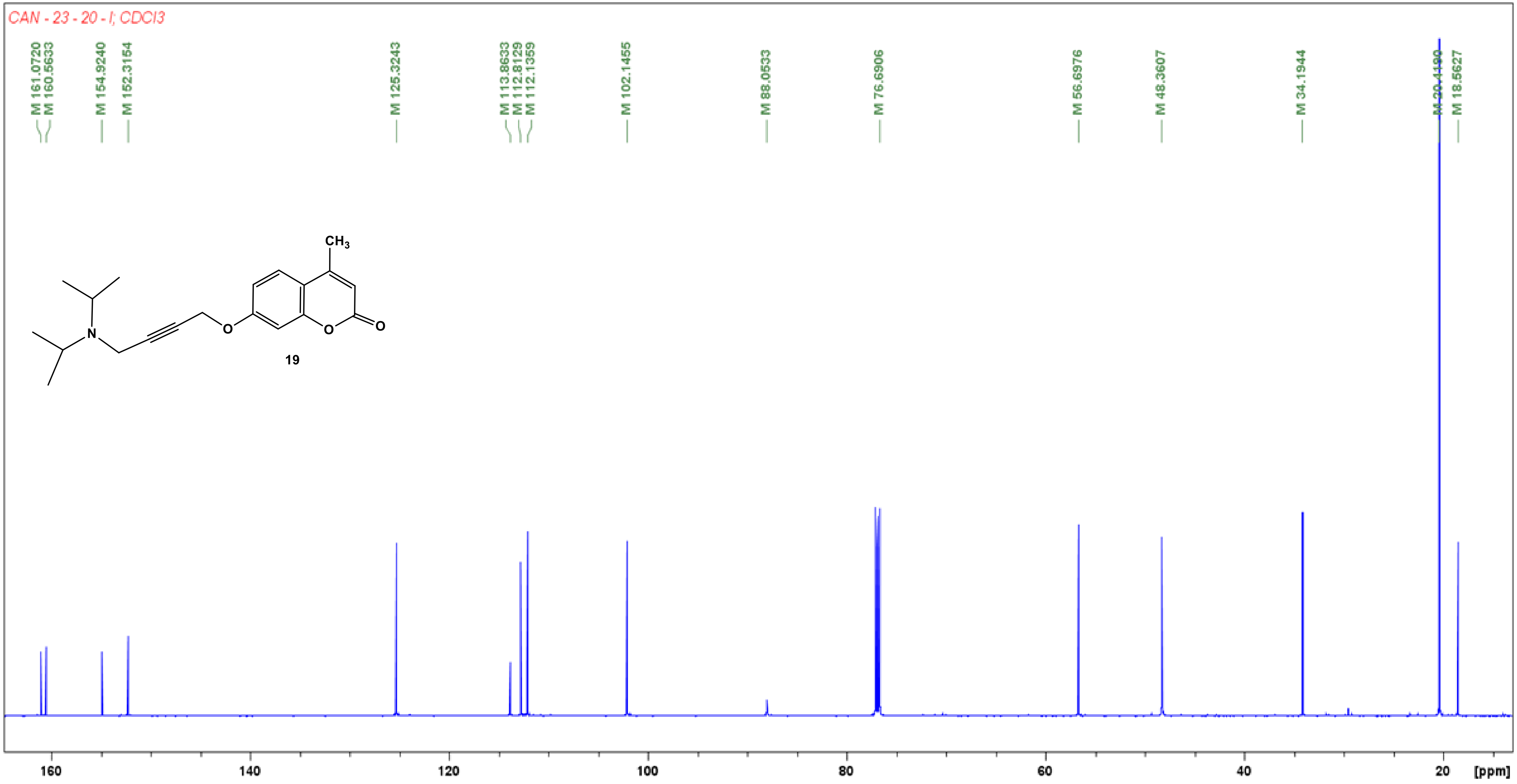

Figure S18. ^{13}C NMR spectrum of 7-(4-(diisopropylamino)but-2-ynyloxy)-4-methyl-2*H*-chromen-2-one (**19**) ($CDCl_3$, 125 MHz).

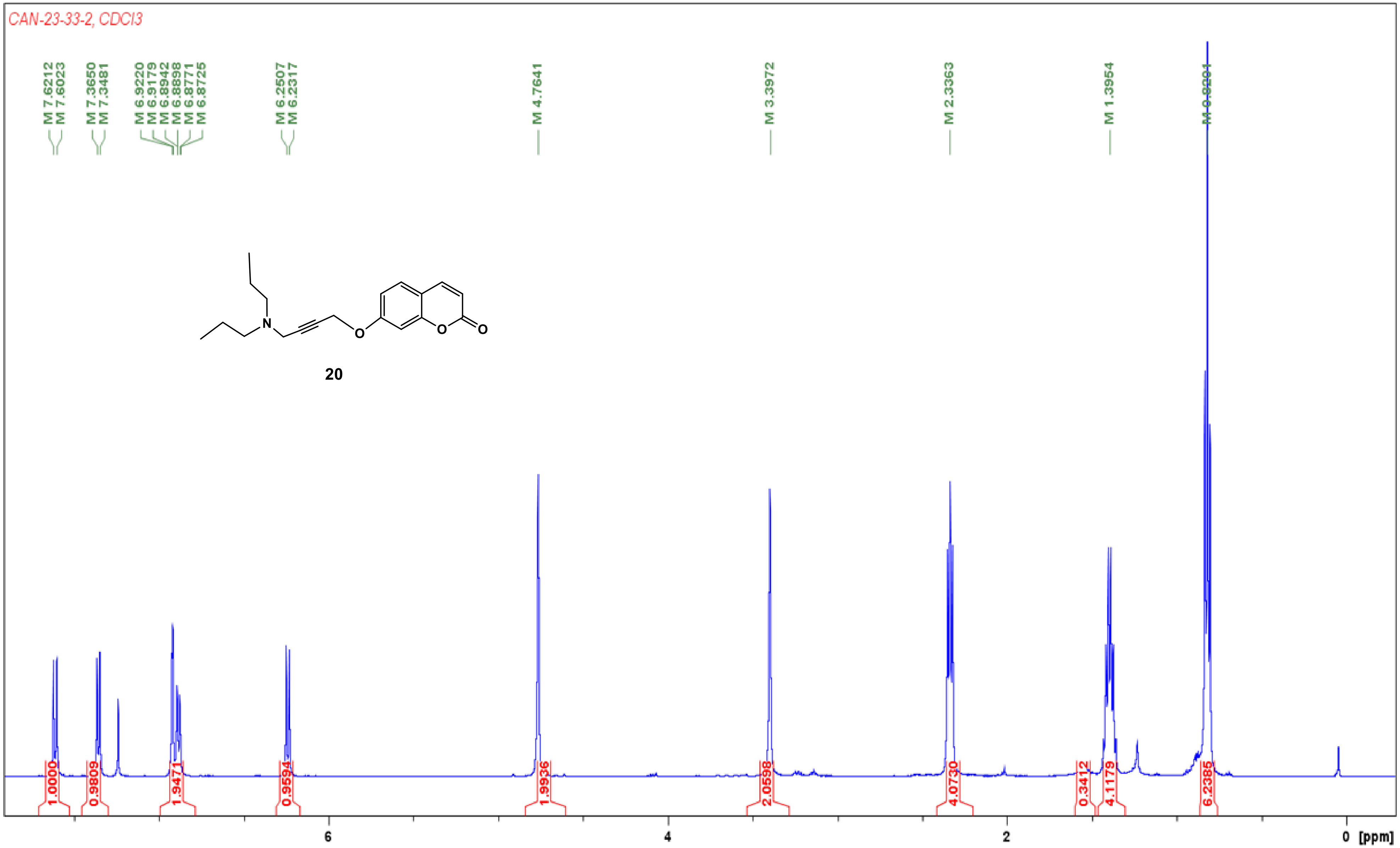

Figure S19. ^{1}H NMR spectrum of 7-(4-(dipropylamino)but-2-ynyloxy)-2*H*-chromen-2-one (**20**) ($CDCl_3$, 300 MHz).

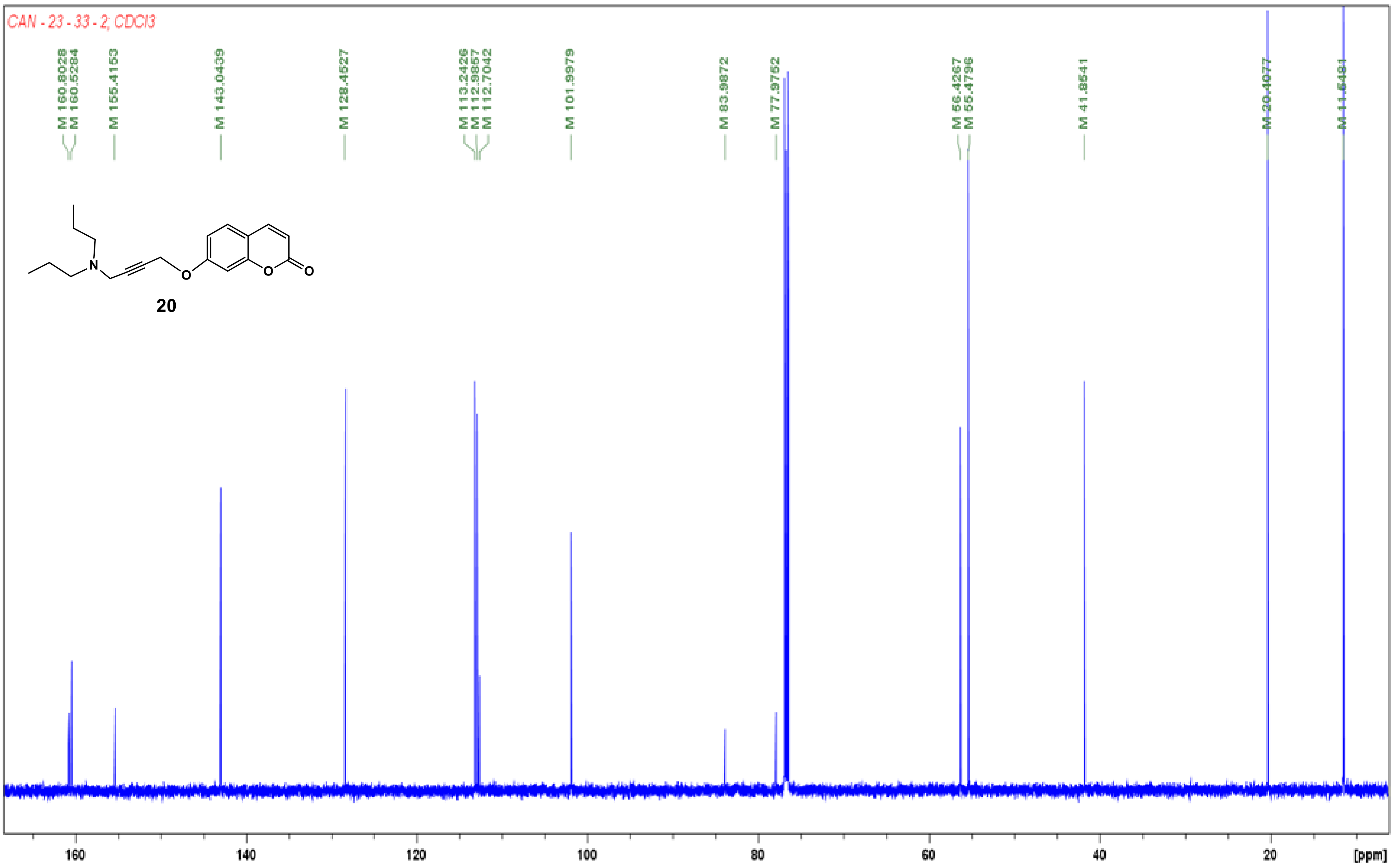

Figure S20. ^{13}C NMR spectrum of 7-(4-(dipropylamino)but-2-ynyloxy)-2*H*-chromen-2-one (**20**) ($CDCl_3$, 125 MHz).

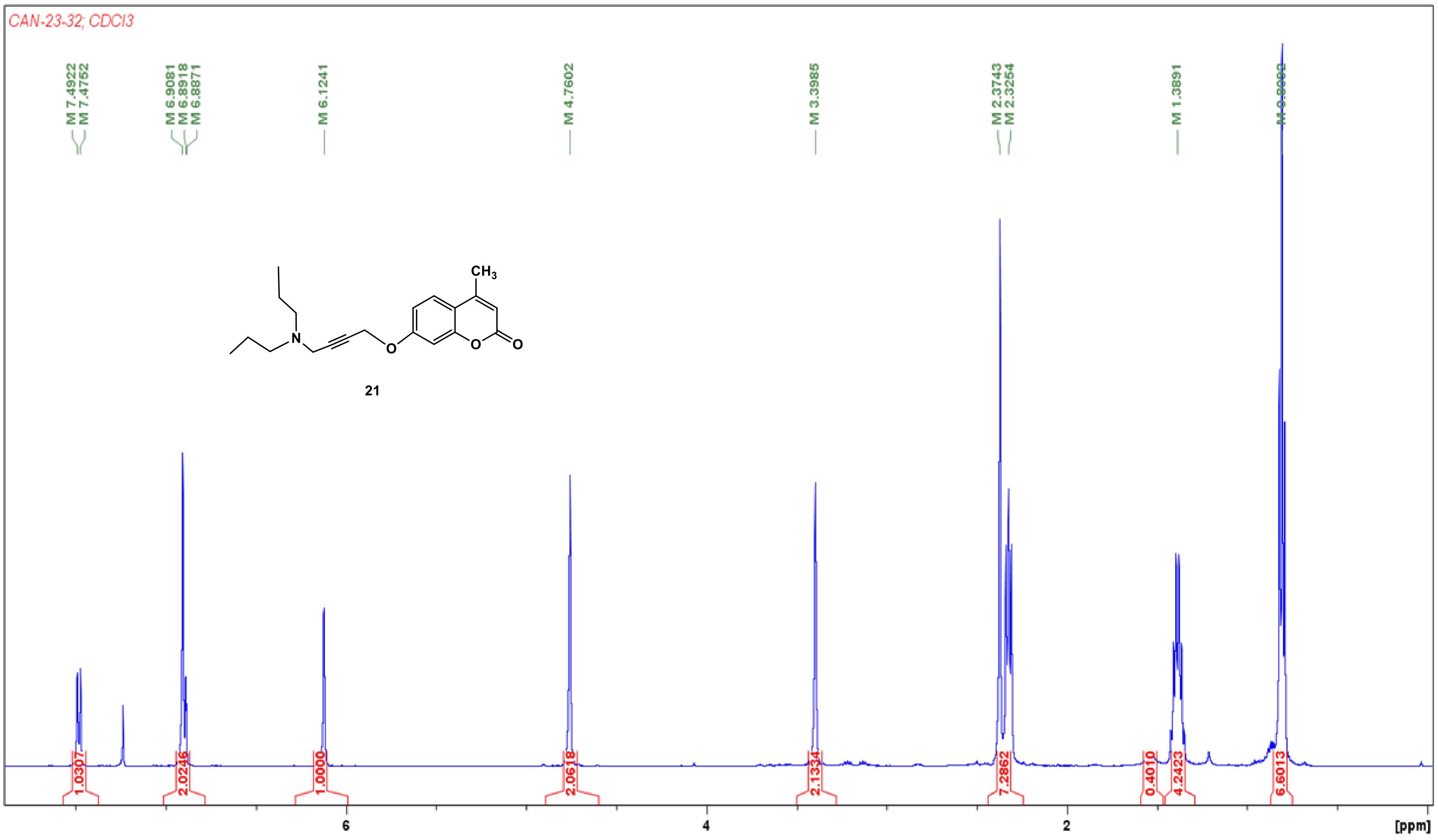

Figure S21. ¹H NMR spectrum of 7-(4-(dipropylamino)but-2-ynyloxy)-4-methyl-2*H*-chromen-2-one (**21**) ($CDCl_3$, 300 MHz).

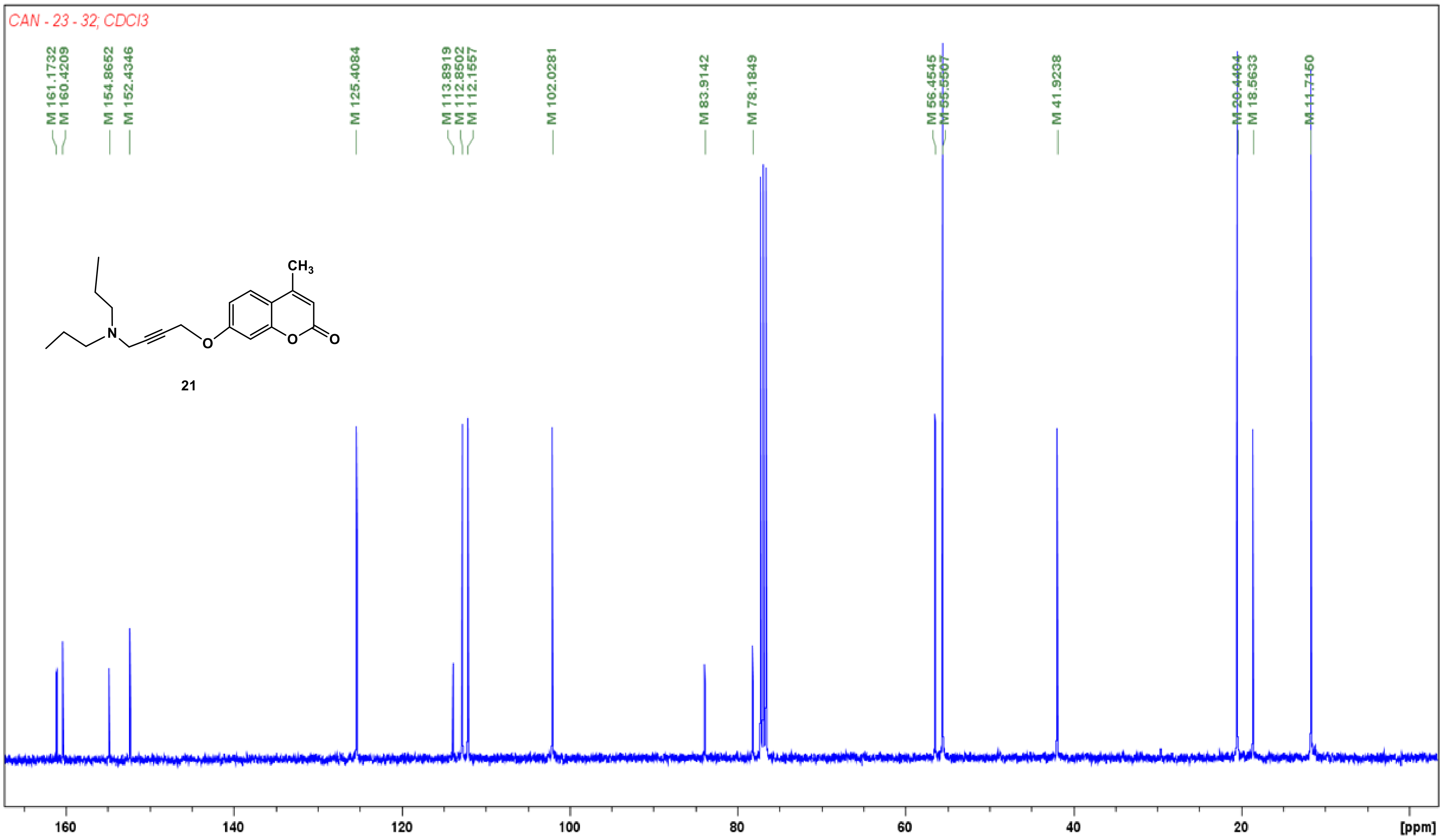

Figure S22. ^{13}C NMR spectrum of 7-(4-(dipropylamino)but-2-ynyloxy)-4-methyl-2*H*-chromen-2-one (**21**) ($CDCl_3$, 125 MHz).

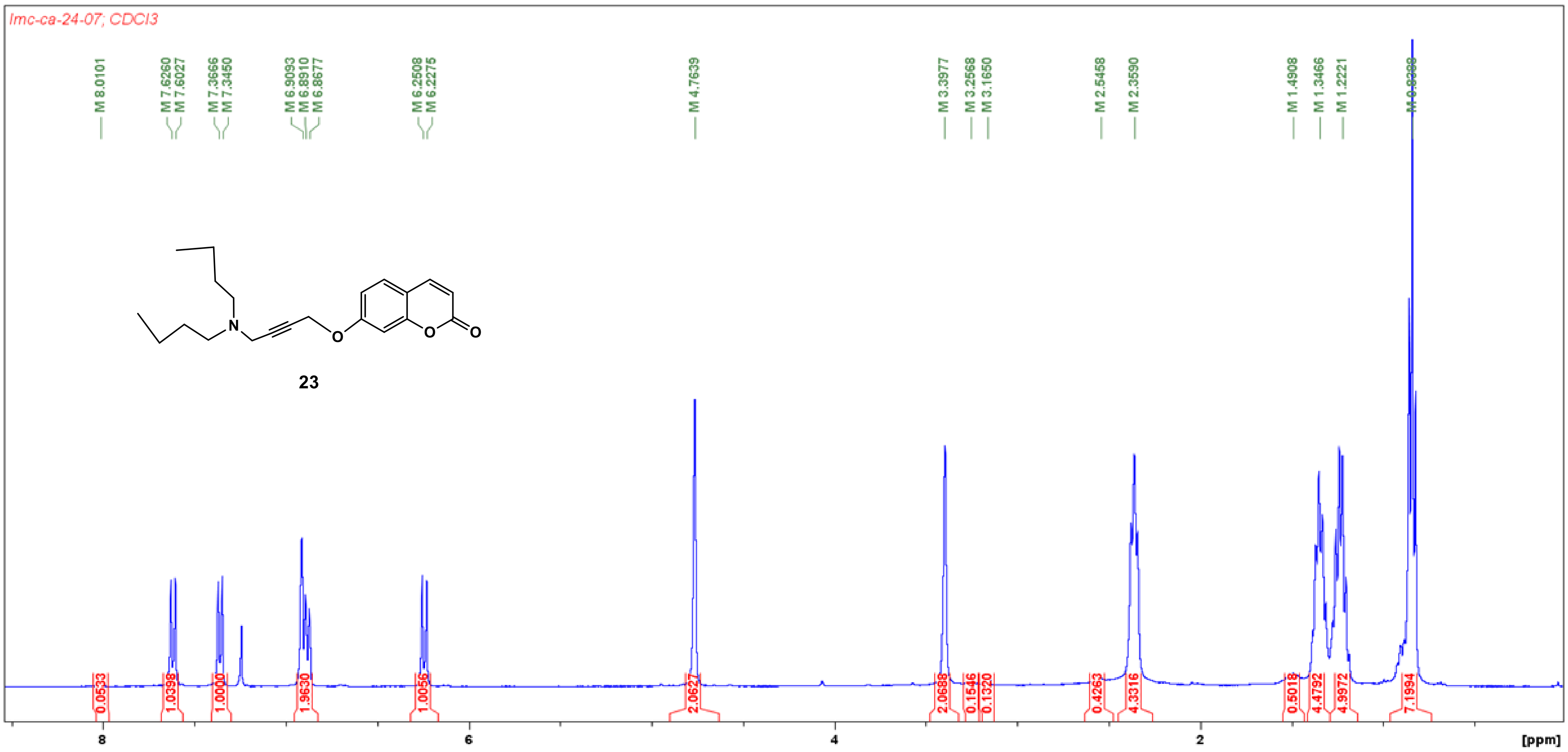

Figure S23. ¹H NMR spectrum of 7-(4-(dibutylamino)but-2-ynyloxy)-2*H*-chromen-2-one (**23**) (CDCl$_3$, 300 MHz).

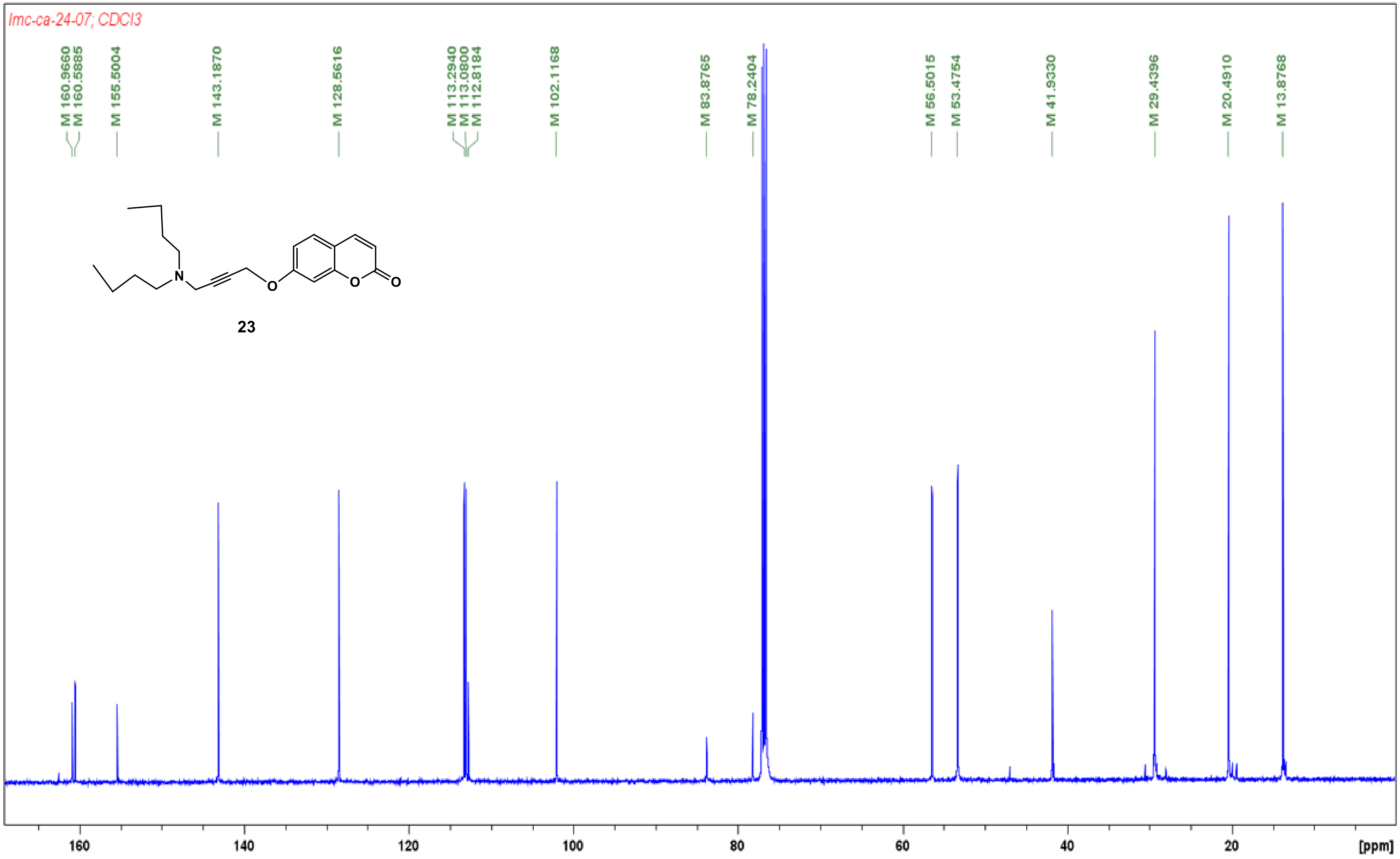

Figure S24. ^{13}C NMR spectrum of 7-(4-(dibutylamino)but-2-ynyloxy)-2*H*-chromen-2-one (**23**) ($CDCl_3$, 125 MHz).

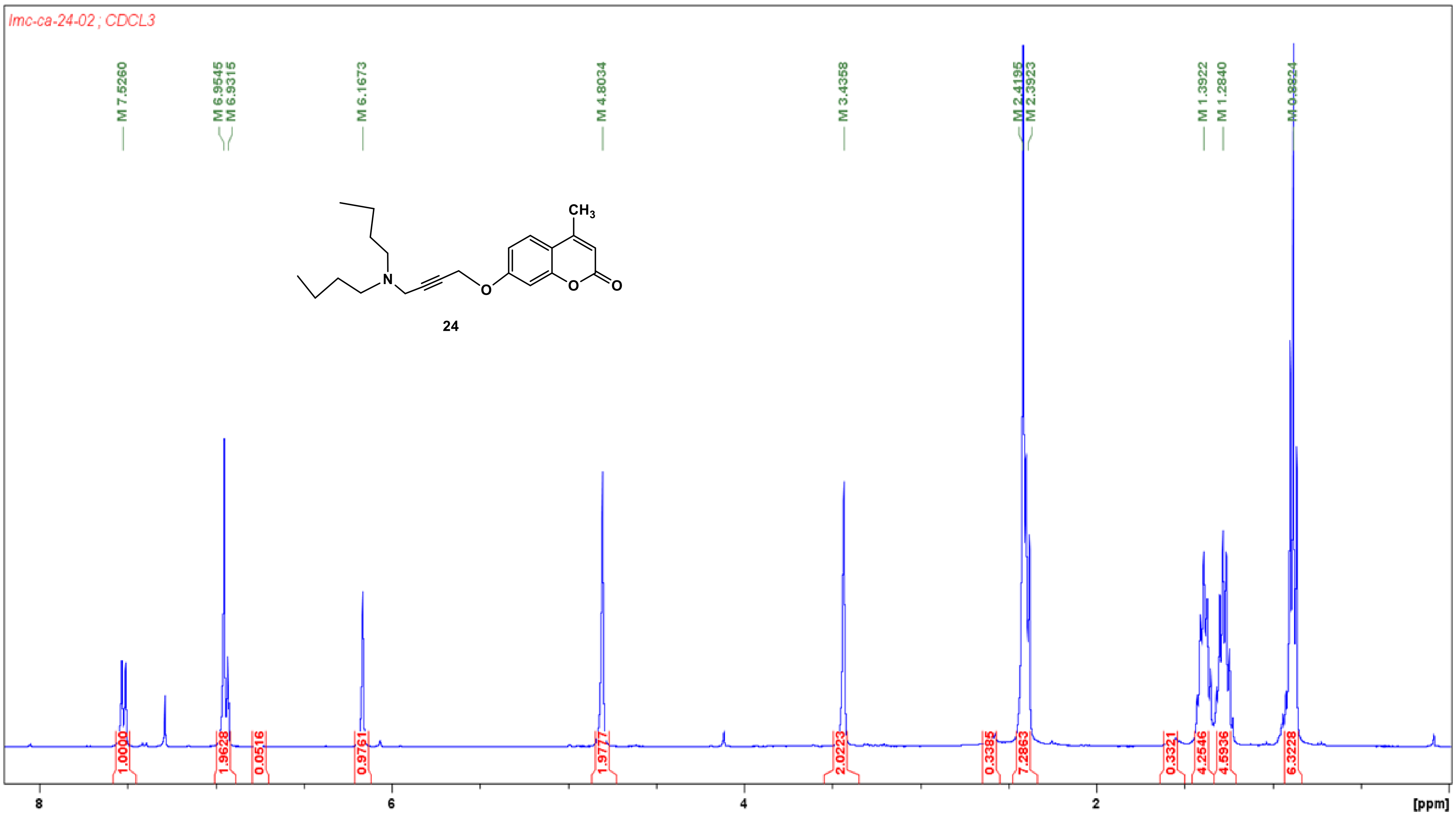

Figure S25. ^{1}H NMR spectrum of 7-(4-(dibutylamino)but-2-ynyloxy)-4-methyl-2*H*-chromen-2-one (**24**) ($CDCl_3$, 300 MHz).

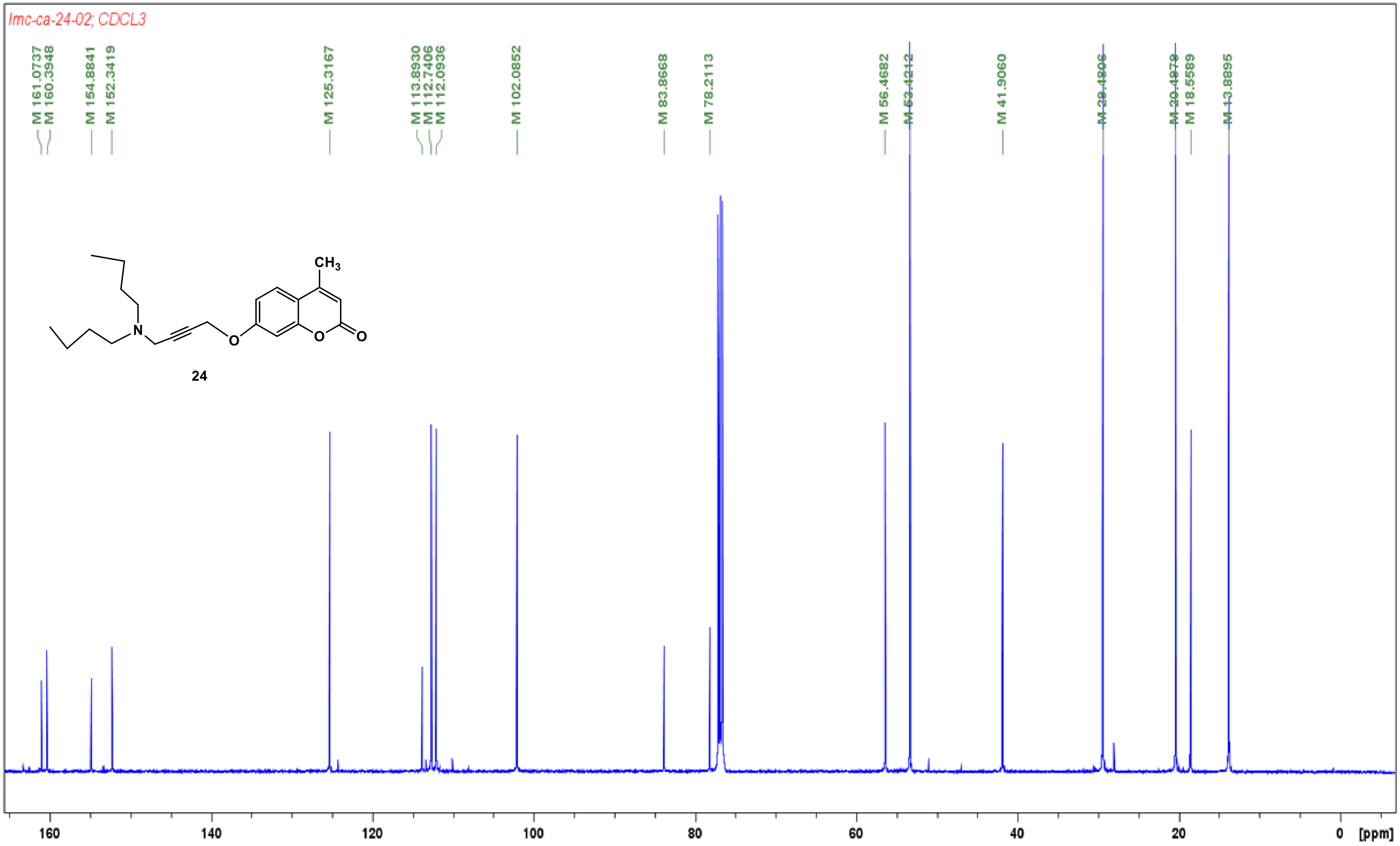

Figure S26. ^{13}C NMR spectrum of 7-(4-(dibutylamino)but-2-ynyloxy)-4-methyl-2*H*-chromen-2-one (**24**) ($CDCl_3$, 125 MHz).

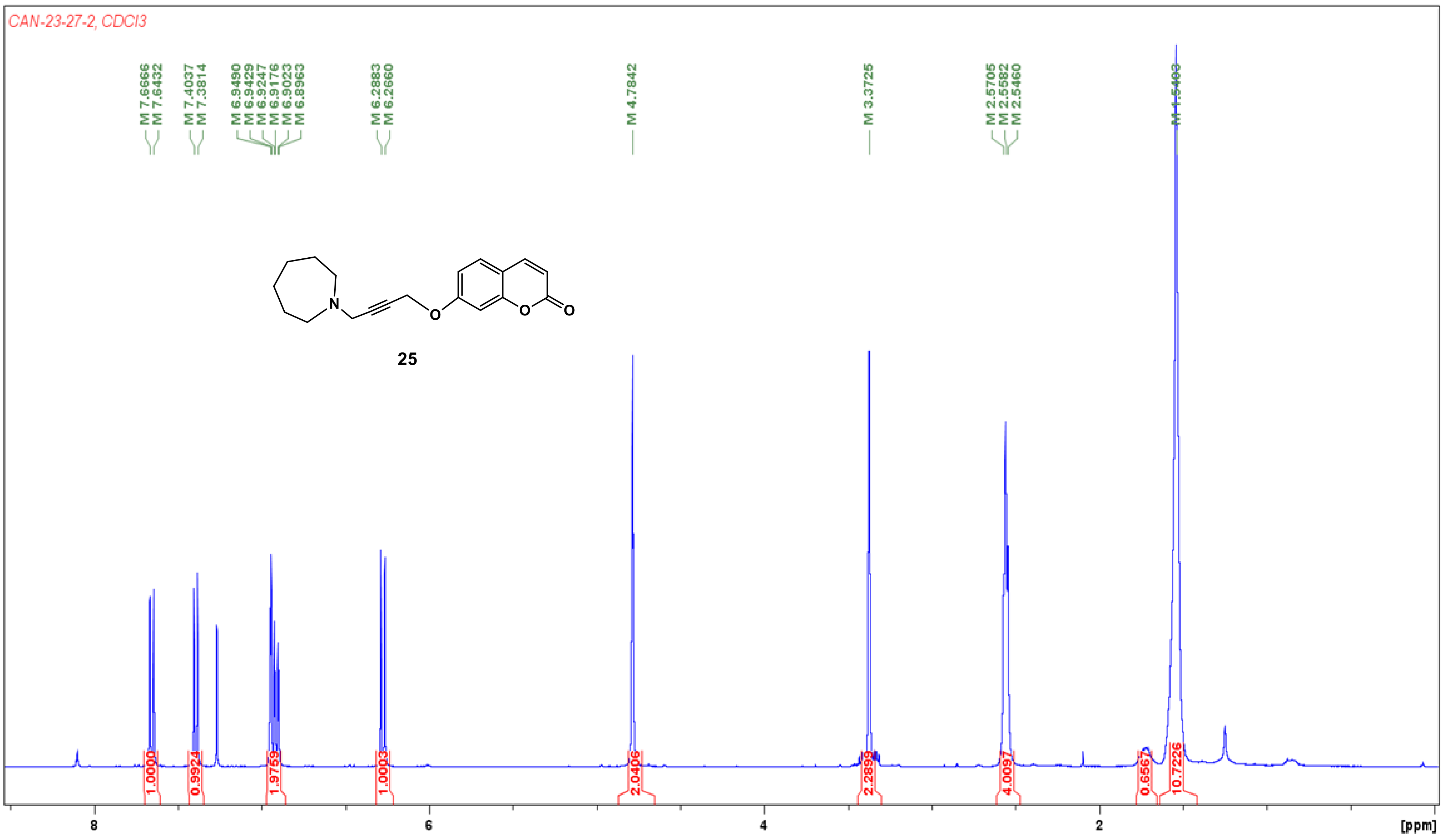

Figure S27. ^{1}H NMR spectrum of 7-(4-(azepan-1-yl)but-2-ynyloxy)-2*H*-chromen-2-one (**25**) ($CDCl_3$, 300 MHz).

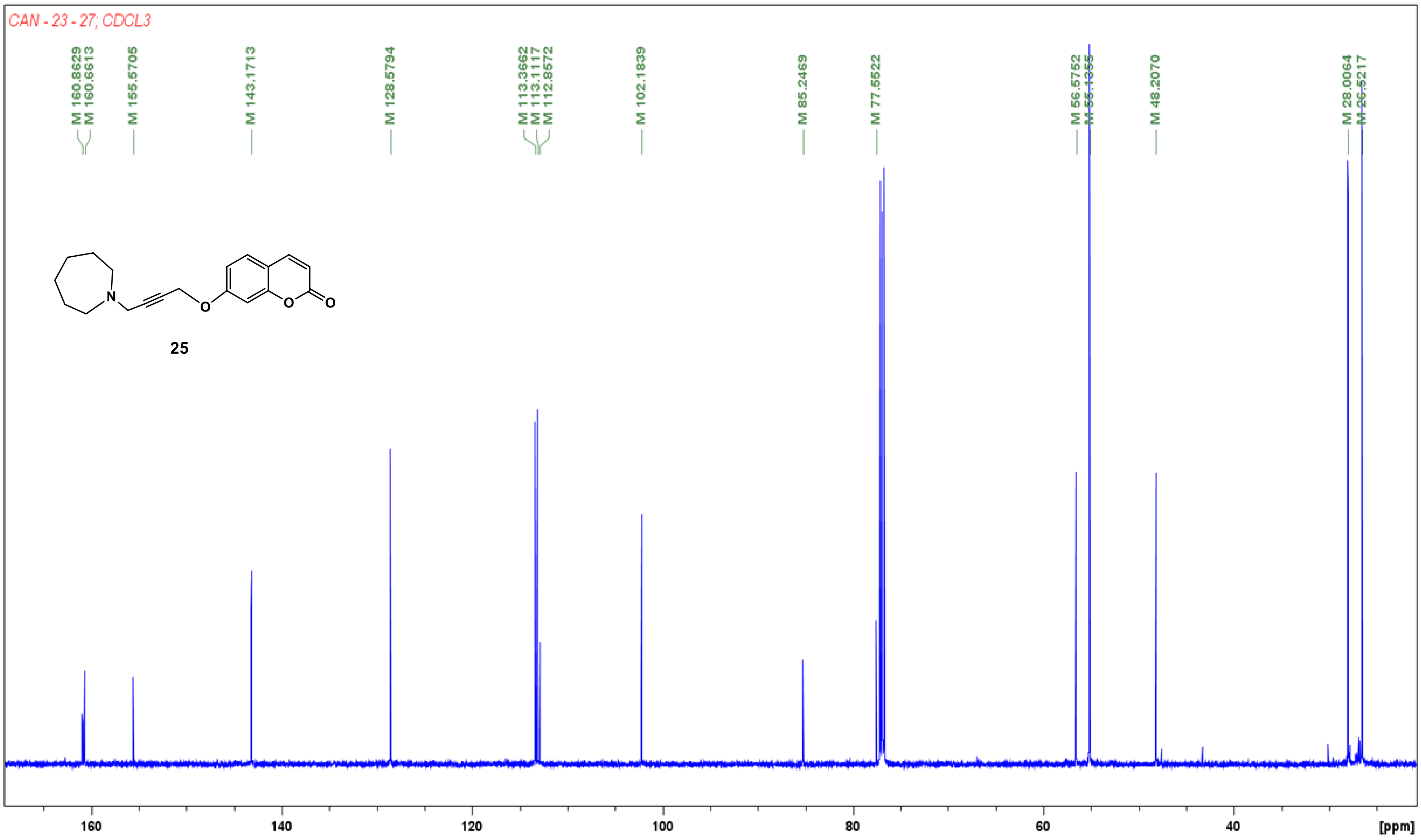

Figure S28. ^{13}C NMR spectrum of 7-(4-(azepan-1-yl)but-2-ynyloxy)-2*H*-chromen-2-one (**25**) ($CDCl_3$,125 MHz).

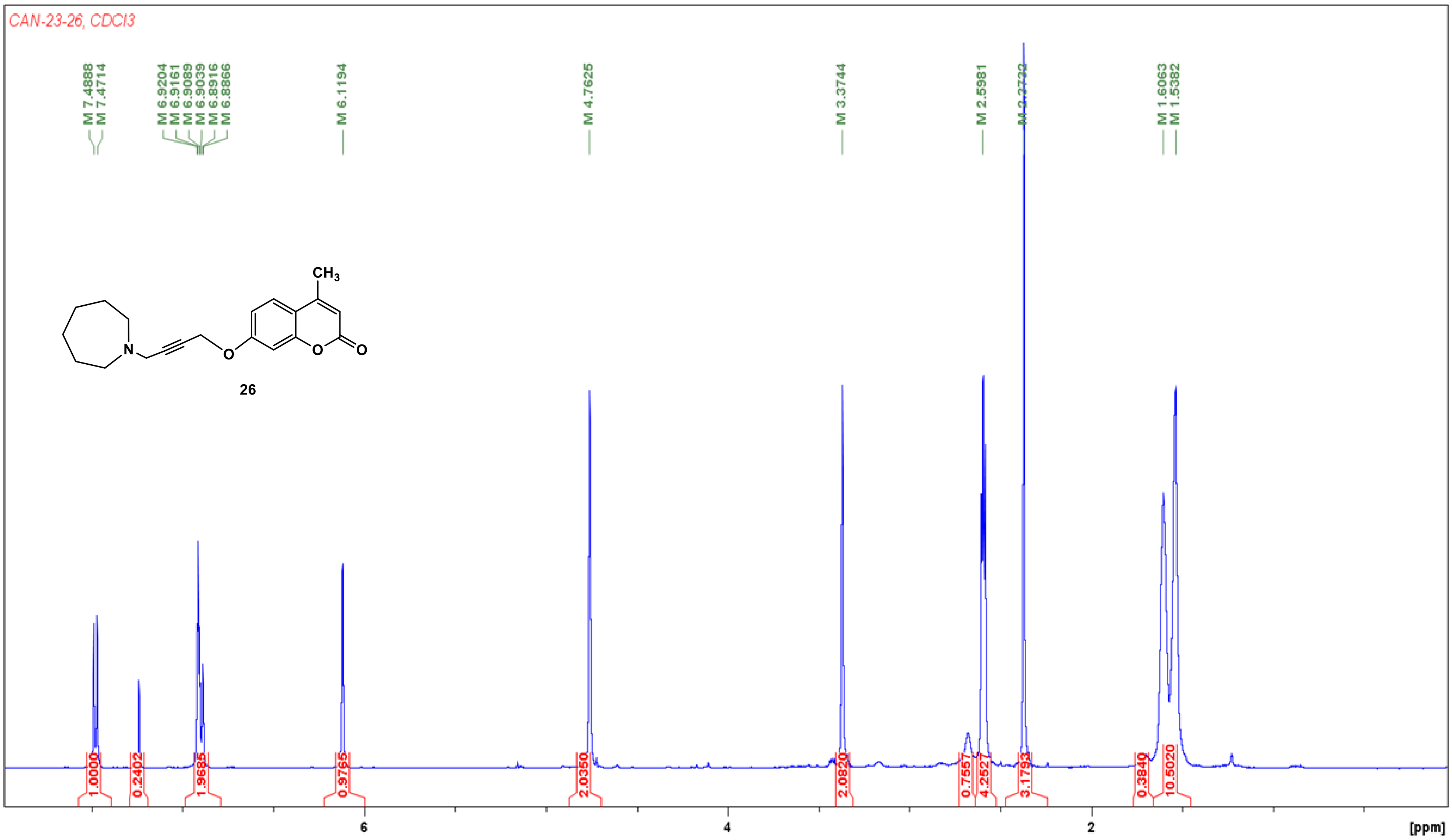

Figure S29. ^{1}H NMR spectrum of 7-(4-(azepan-1-yl)but-2-ynyloxy)-4-methyl-2*H*-chromen-2-one (**26**) ($CDCl_3$, 300 MHz).

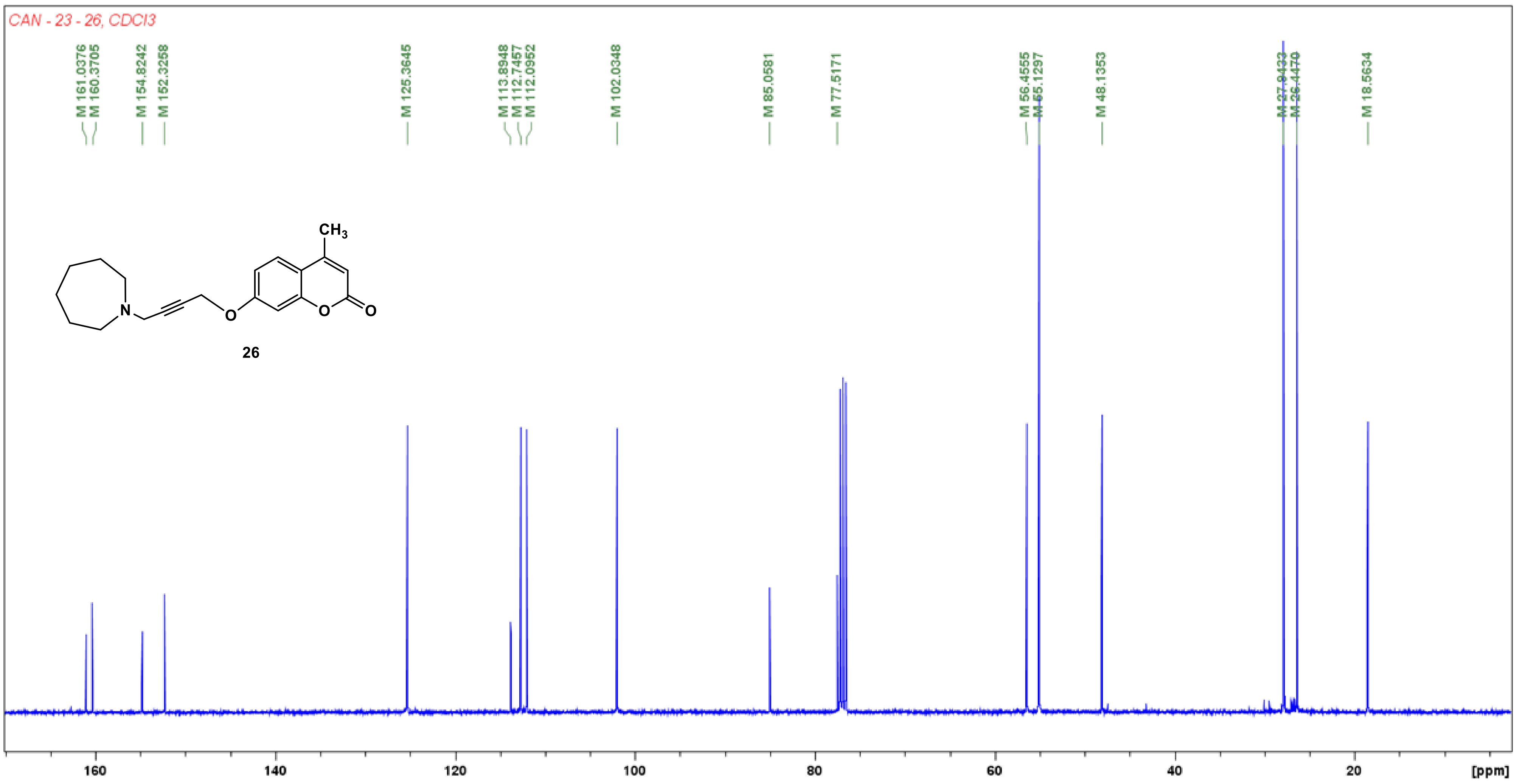

Figure S30. ^{13}C NMR spectrum of 7-(4-(azepan-1-yl)but-2-ynyloxy)-4-methyl-2*H*-chromen-2-one (**26**) ($CDCl_3$,125 MHz).

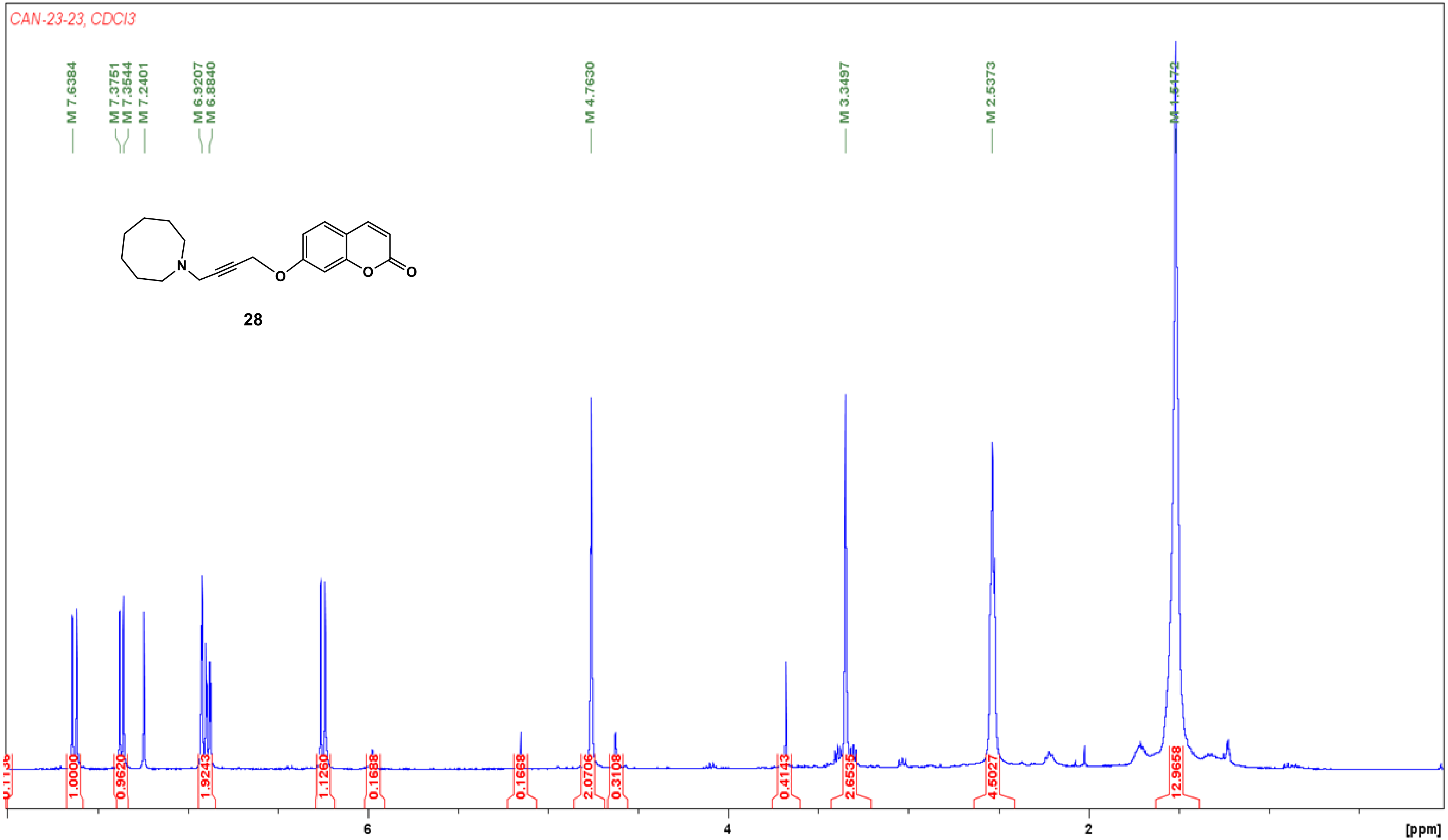

Figure S31. ¹H NMR spectrum of 7-(4-(azocan-1-yl)but-2-ynyloxy)-2*H*-chromen-2-one (**28**) ($CDCl_3$, 300 MHz).

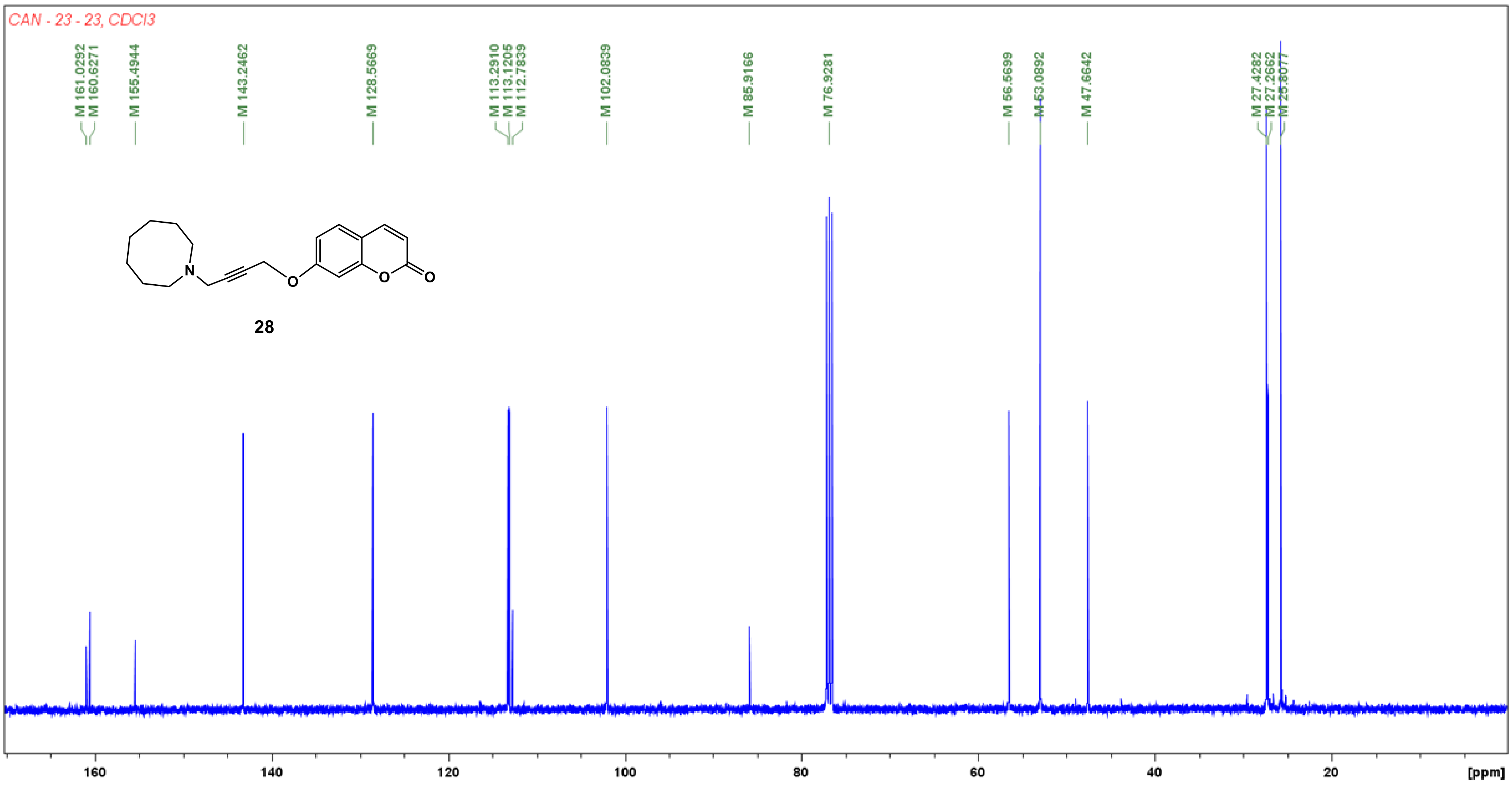

Figure S32. ^{13}C NMR spectrum of 7-(4-(azocan-1-yl)but-2-ynyloxy)-2*H*-chromen-2-one (**28**) ($CDCl_3$,125 MHz).

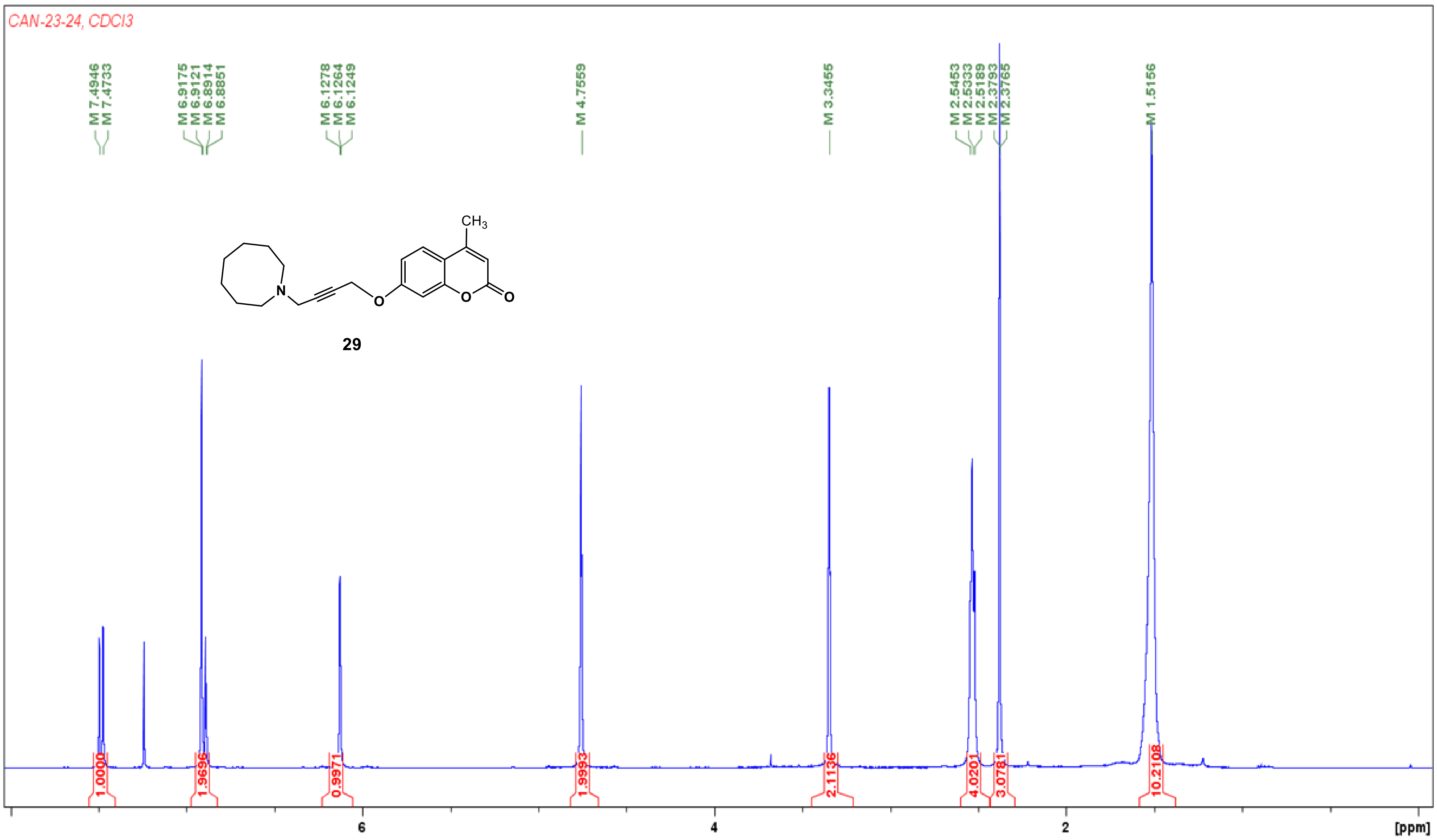

Figure S33. ^{1}H NMR spectrum of 7-(4-(azocan-1-yl)but-2-ynyloxy)-4-methyl-2*H*-chromen-2-one (**29**) ($CDCl_3$, 300 MHz).

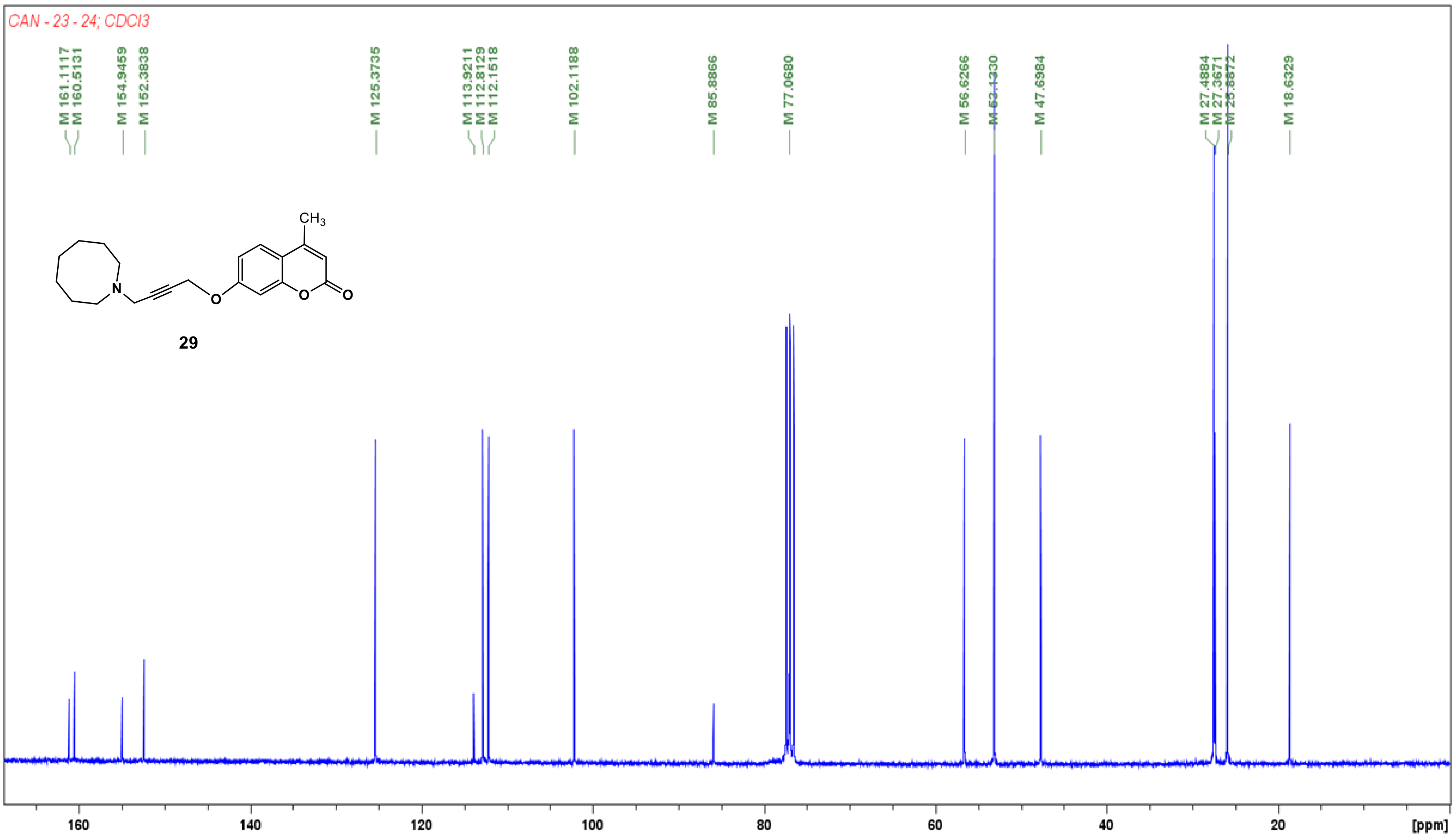

Figure S34. ¹³C NMR spectrum of 7-(4-(azocan-1-yl)but-2-ynyloxy)-4-methyl-2*H*-chromen-2-one (**29**) ($CDCl_3$,125 MHz).

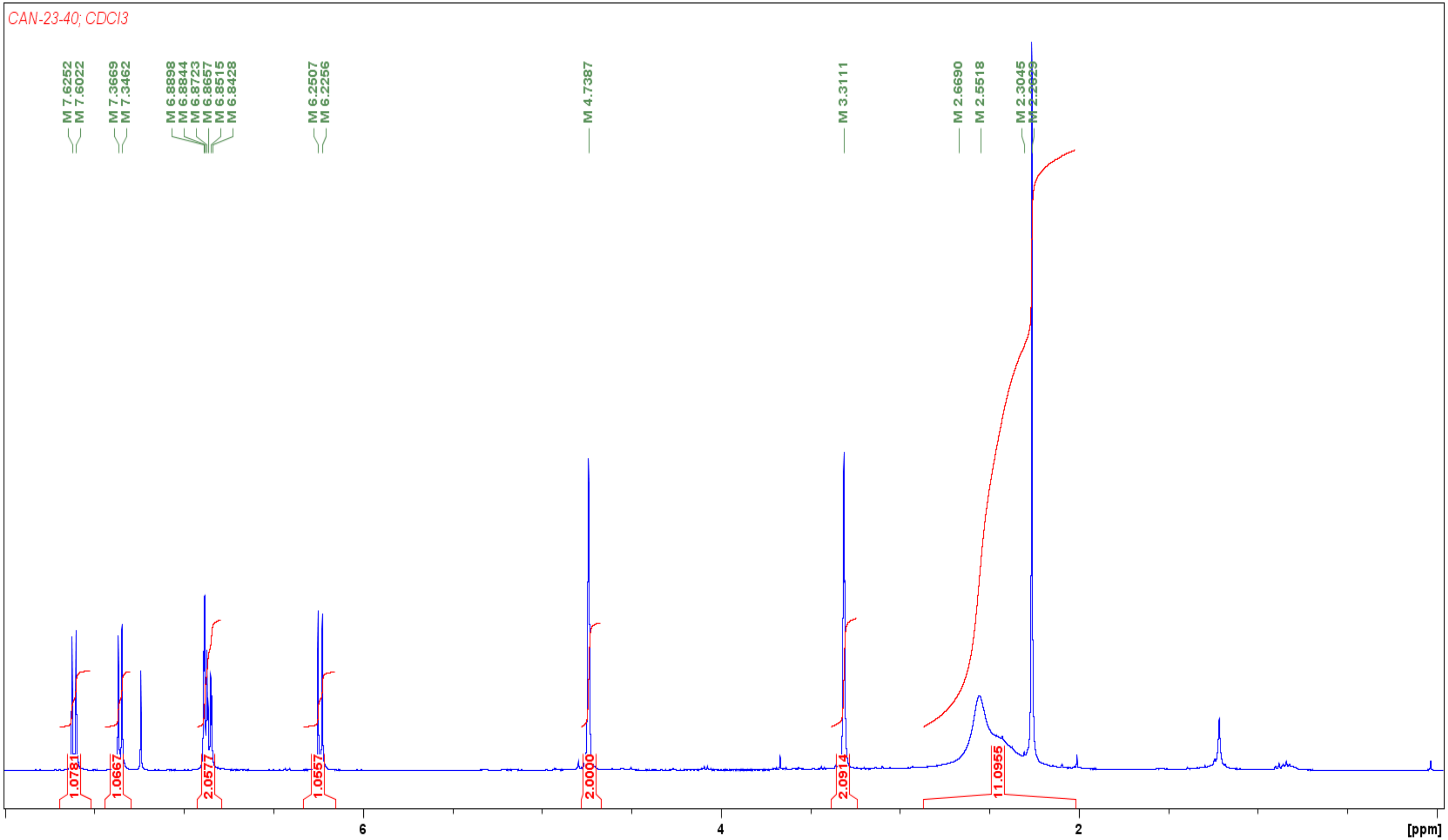

Figure S35. ^{1}H NMR spectrum of 7-(4-(4-methylpiperazin-1-yl)but-2-ynyloxy)-2*H*-chromen-2-one (**30**) ($CDCl_3$, 300 MHz).

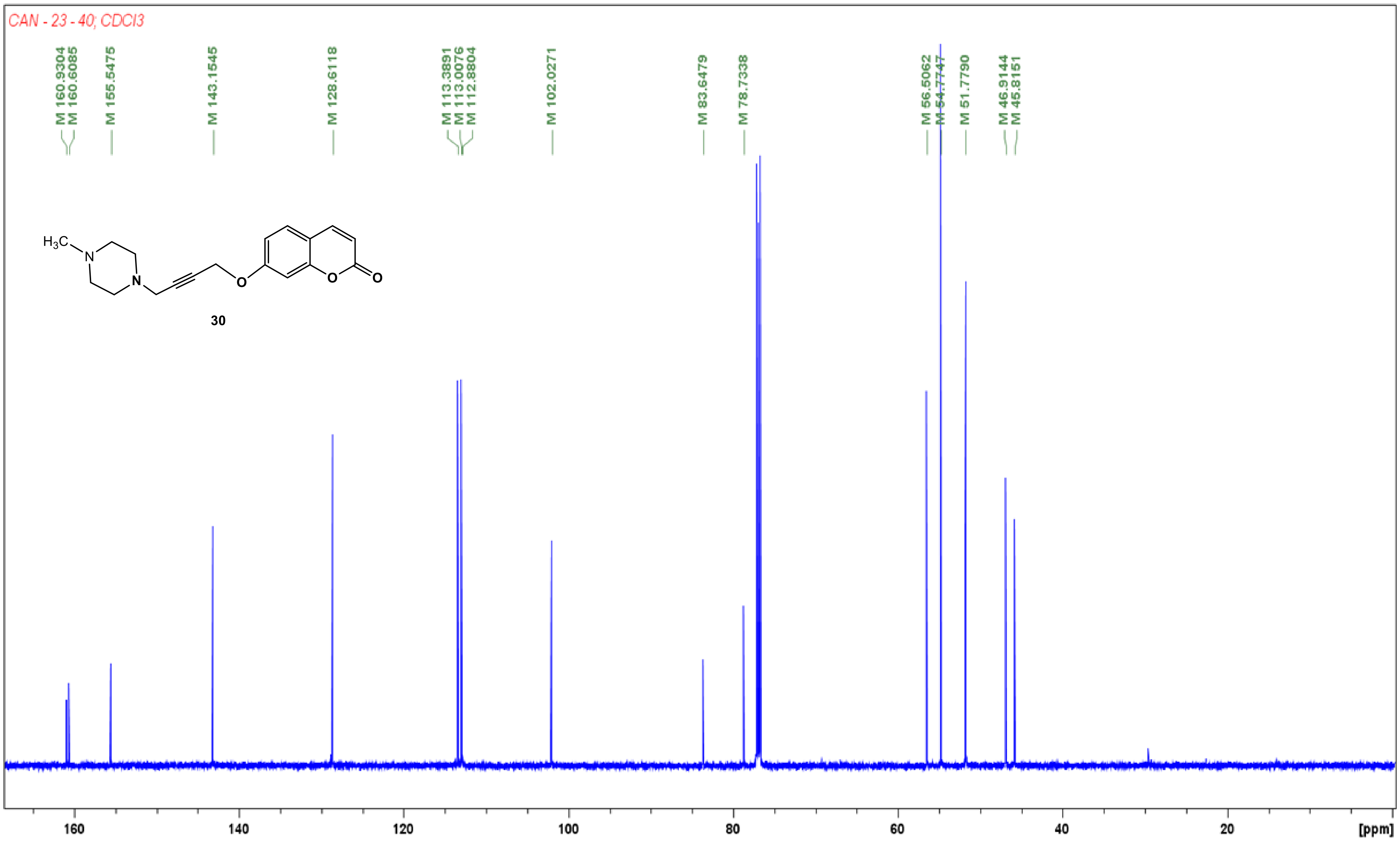

Figure S36. ^{13}C NMR spectrum of 7-(4-(4-methylpiperazin-1-yl)but-2-ynyloxy)-2*H*-chromen-2-one (**30**) ($CDCl_3$, 125 MHz).

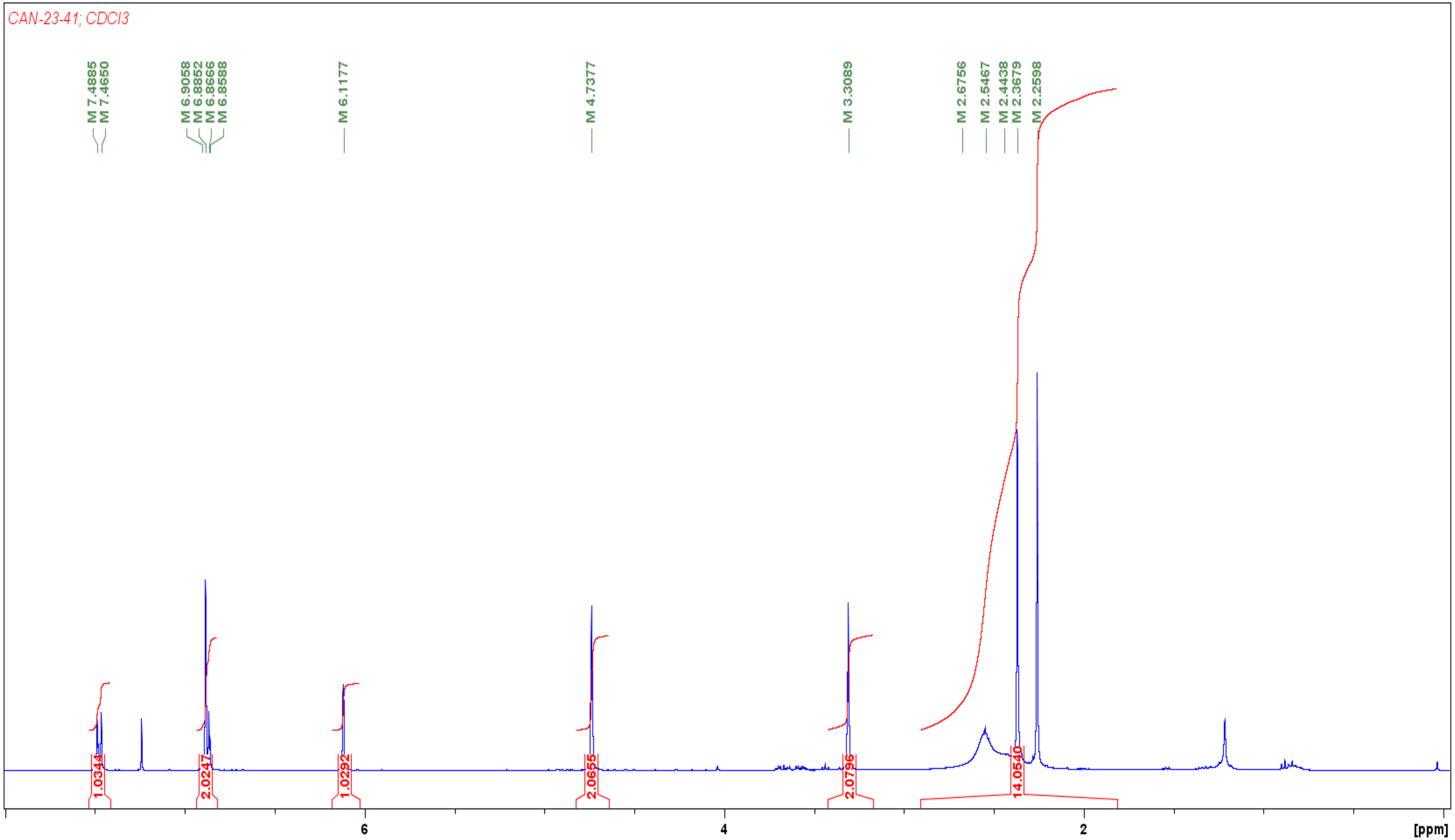

Figure S37. ^{1}H NMR spectrum of 7-(4-(4-methylpiperazin-1-yl)but-2-ynyloxy)-4-methyl-2*H*-chromen-2-one (**31**) ($CDCl_3$, 300 MHz).

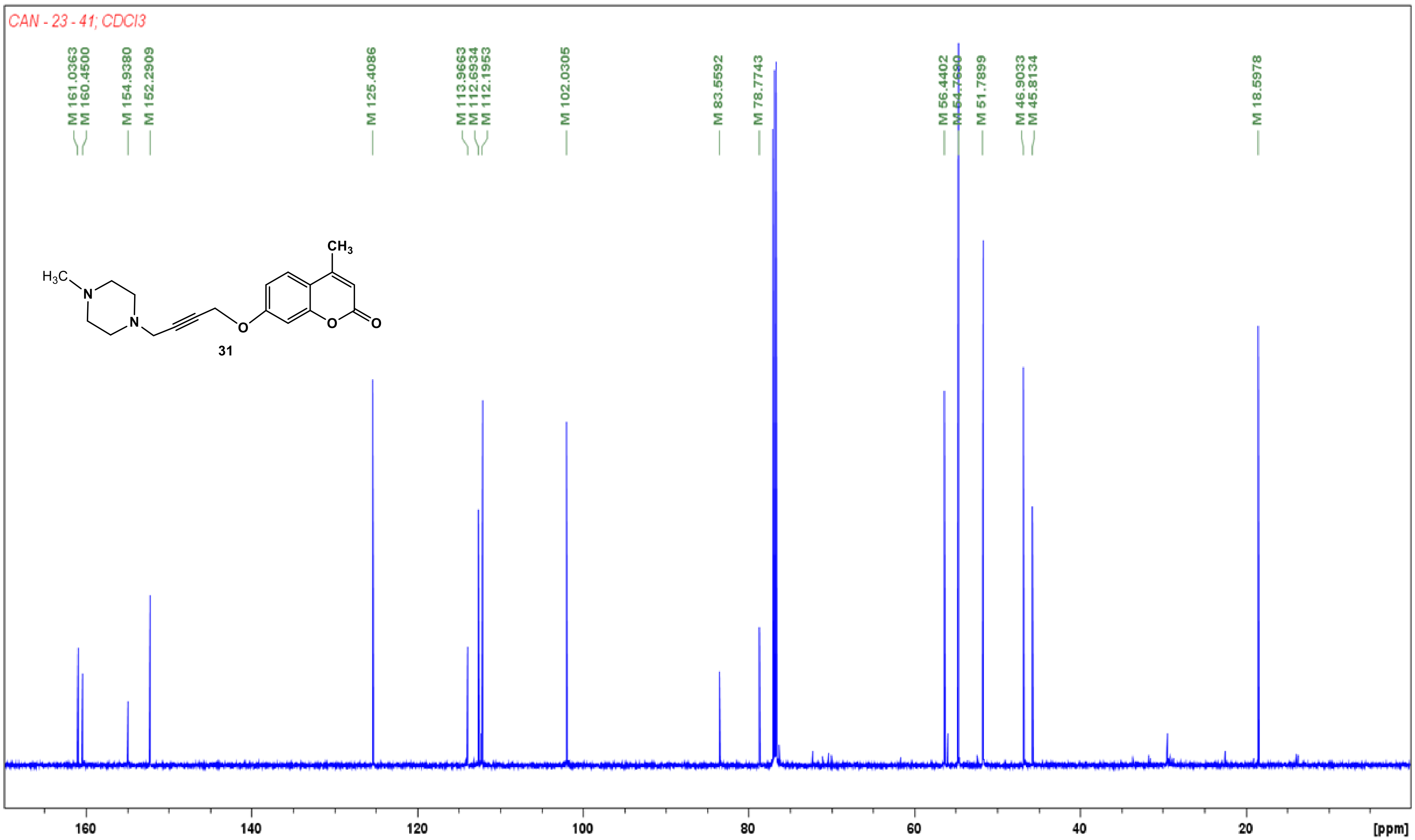

Figure S38. ^{13}C NMR spectrum of 7-(4-(4-methylpiperazin-1-yl)but-2-ynyloxy)-4-methyl-2*H*-chromen-2-one (**31**) ($CDCl_3$, 125 MHz).

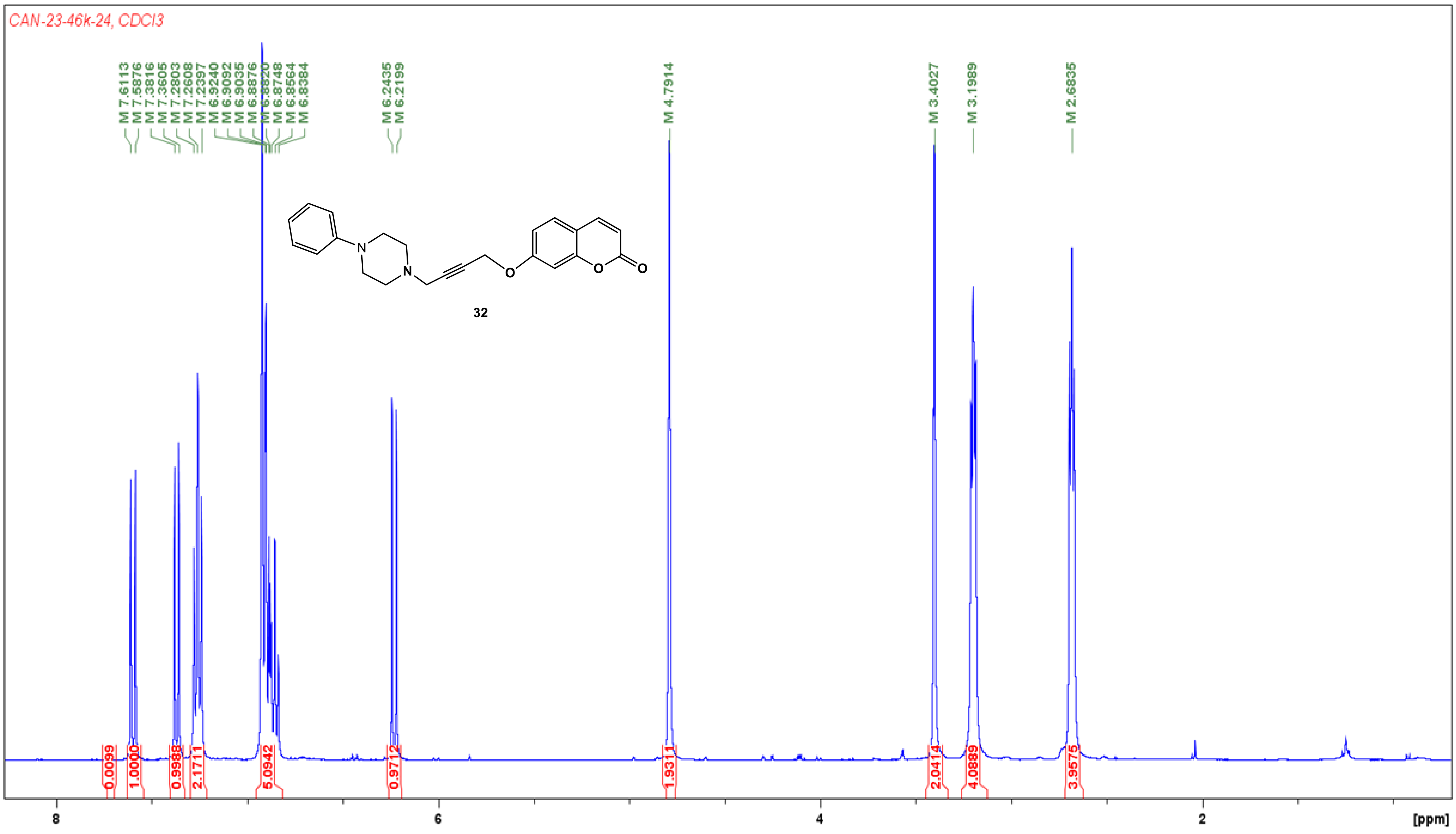

Figure S39. ^{1}H NMR spectrum of 7-(4-(4-phenylpiperazin-1-yl)but-2-ynyloxy)-2*H*-chromen-2-one (**32**) ($CDCl_3$, 300 MHz).

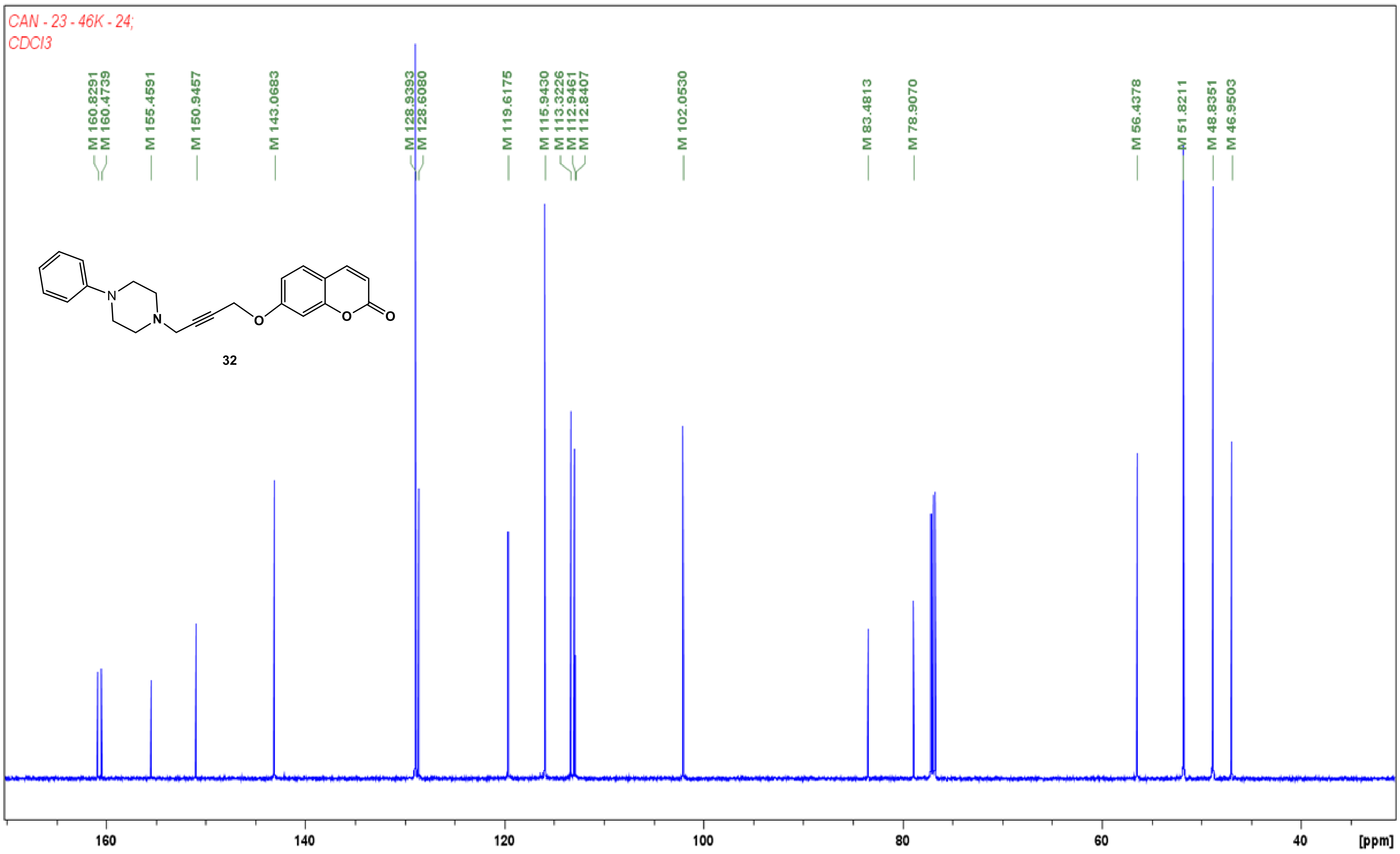

Figure S40. ^{13}C NMR spectrum of 7-(4-(4-phenylpiperazin-1-yl)but-2-ynyloxy)-2*H*-chromen-2-one (**32**) ($CDCl_3$, 125 MHz).

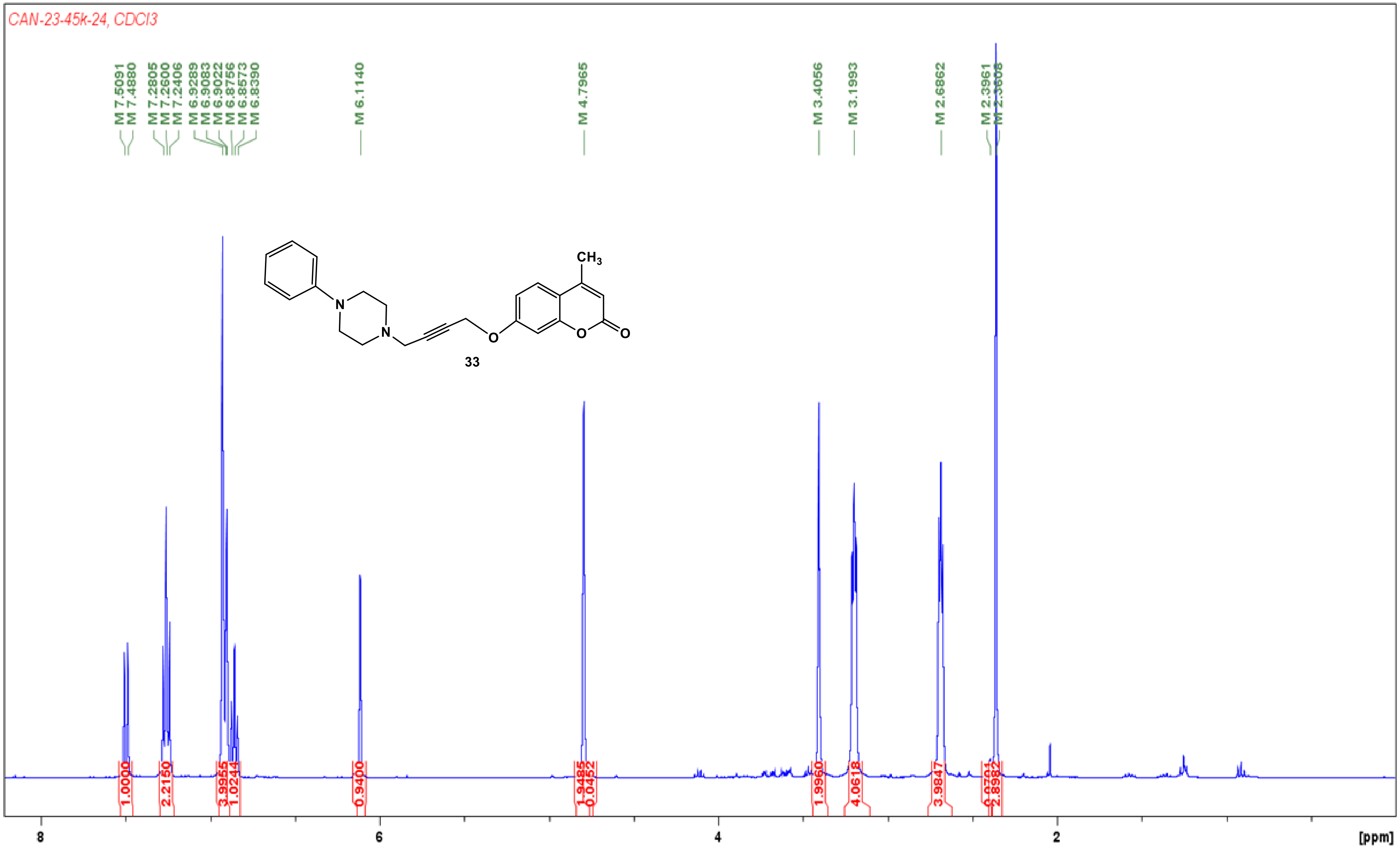

Figure S41. ^{1}H NMR spectrum of 7-(4-(4-phenylpiperazin-1-yl)but-2-ynyloxy)-4-methyl-2*H*-chromen-2-one (**33**) ($CDCl_3$, 300 MHz).

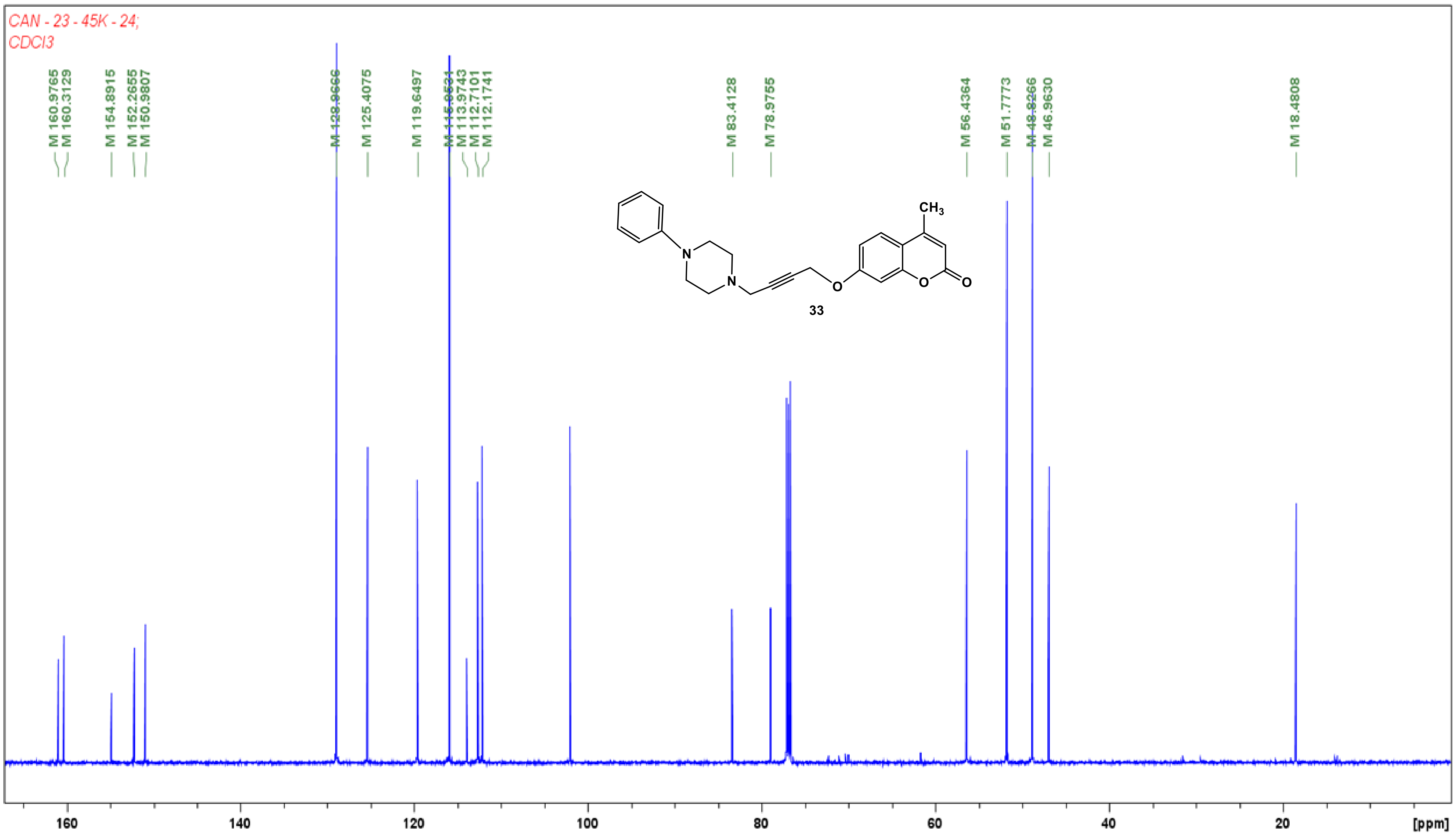

Figure S42. ^{13}C NMR spectrum of 7-(4-(4-phenylpiperazin-1-yl)but-2-ynyloxy)-4-methyl-2*H*-chromen-2-one (**33**) ($CDCl_3$, 125 MHz).

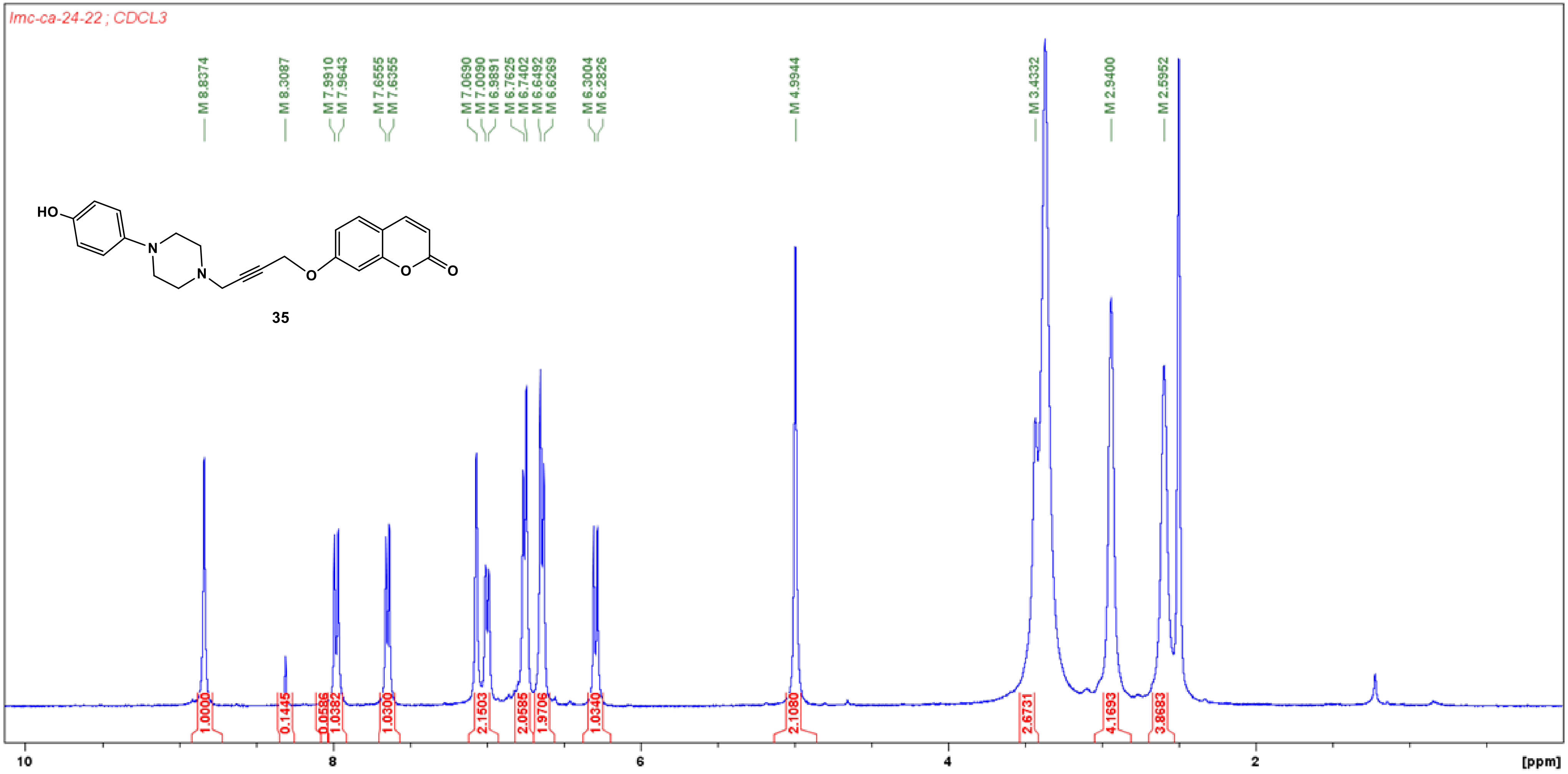

Figure S43. ^{1}H NMR spectrum of 7-(4-(4-(4-hydroxyphenyl)piperazin-1-yl)but-2-ynyloxy)-2*H*-chromen-2-one (**35**) $(CD_3)_2SO$, 300 MHz).

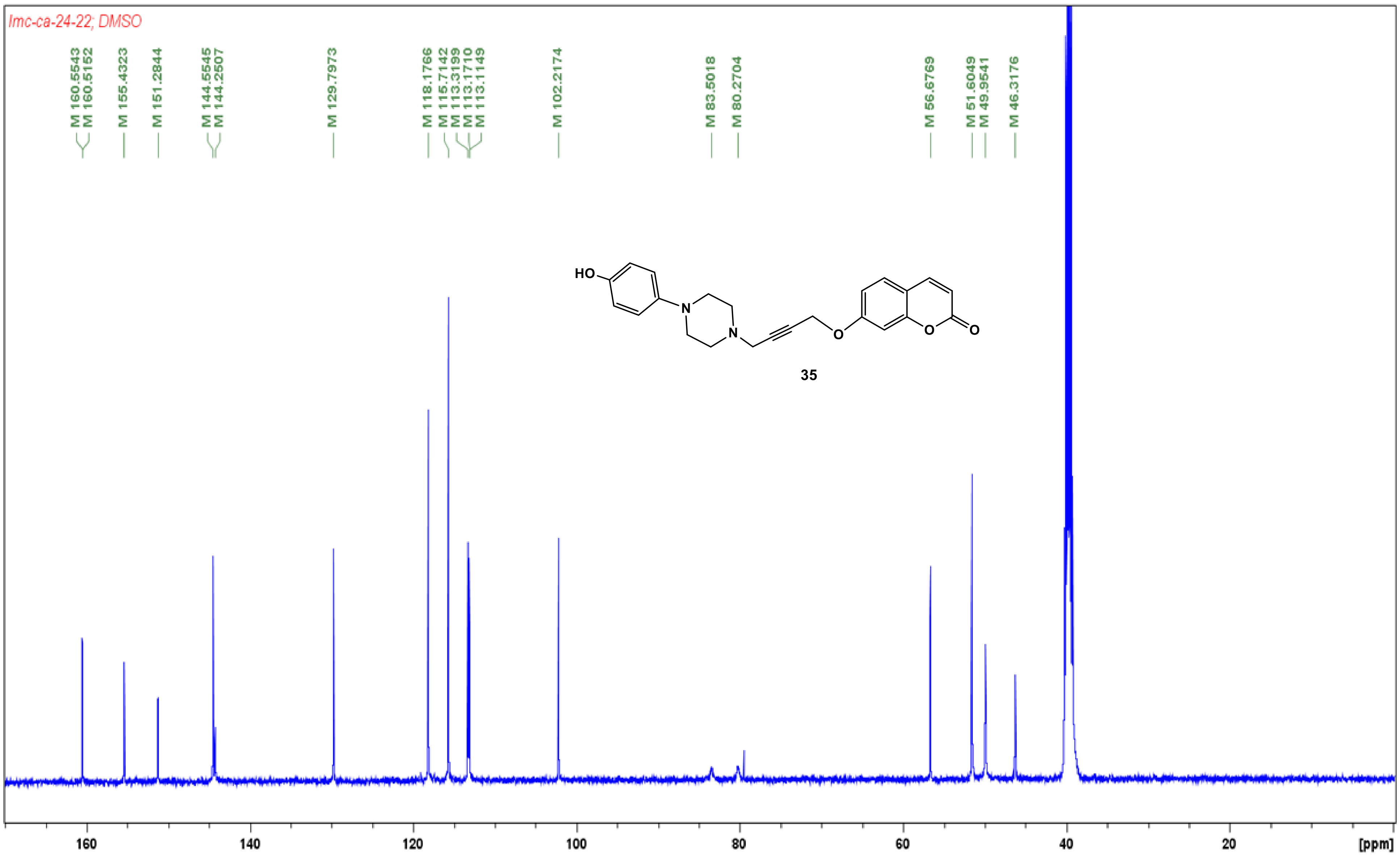

Figure S44. ^{13}C NMR spectrum of 7-(4-(4-(4-hydroxyphenyl)piperazin-1-yl)but-2-ynyloxy)-2*H*-chromen-2-one (**35**) $(CD_3)_2SO$, 125 MHz).

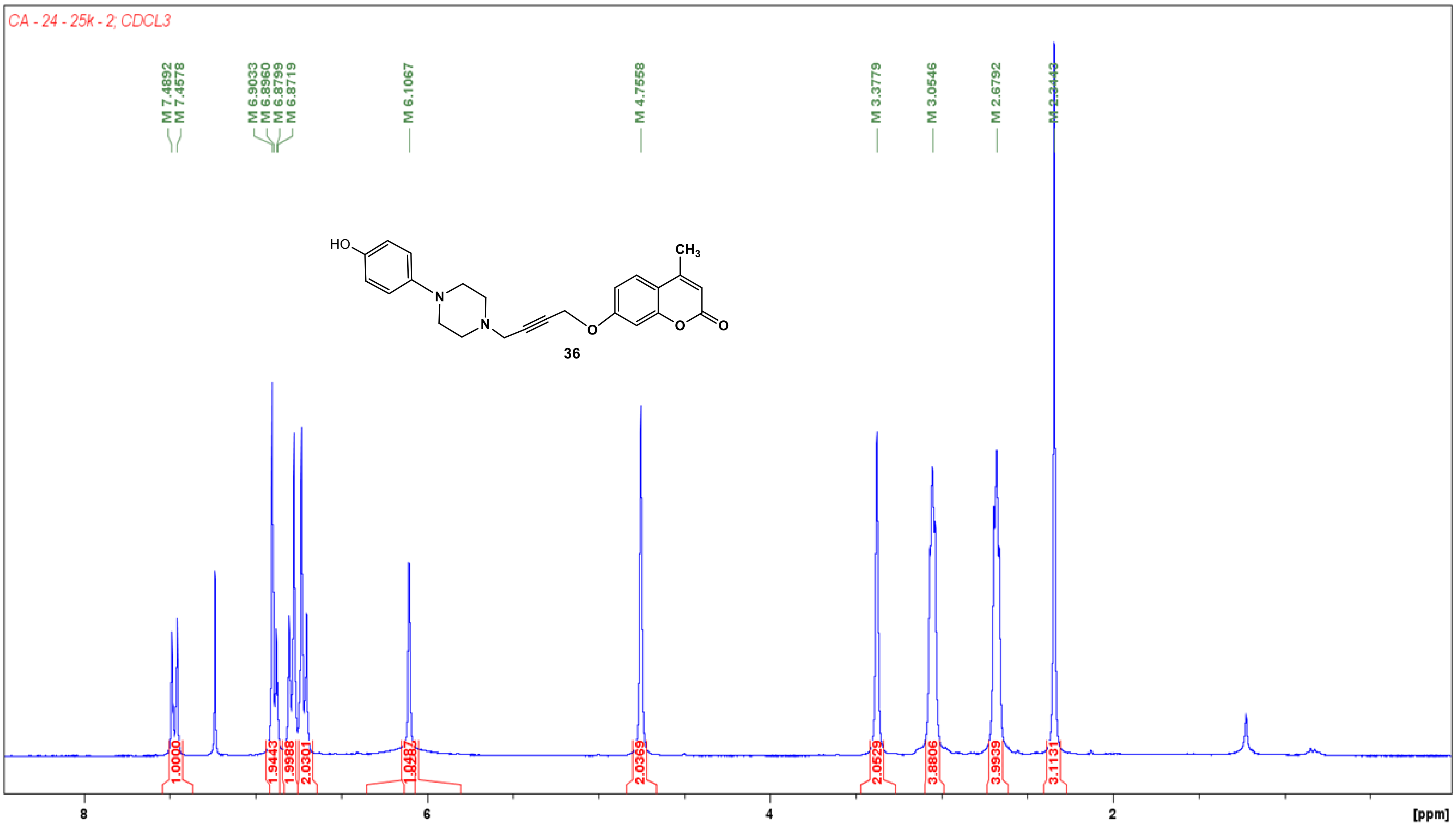

Figure S45. ^{1}H NMR spectrum of 7-(4-(4-(4-hydroxyphenyl)piperazin-1-yl)but-2-ynyloxy)-4-methyl-2*H*-chromen-2-one (**36**) ($CDCl_3$, 300 MHz).

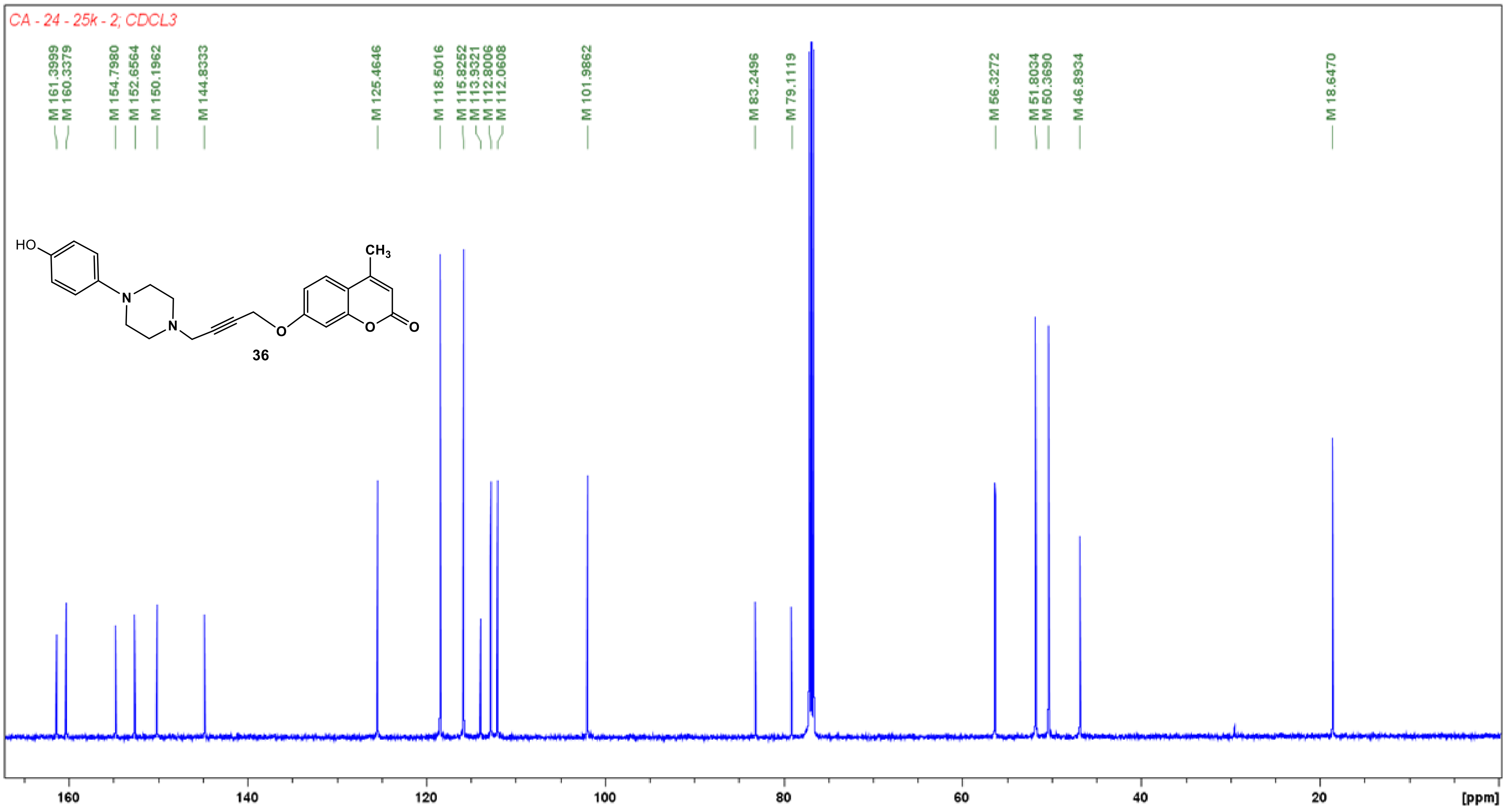

Figure S46. ^{13}C NMR spectrum of 7-(4-(4-(4-hydroxyphenyl)piperazin-1-yl)but-2-ynyloxy)-4-methyl-2*H*-chromen-2-one (**36**) ($CDCl_3$, 125 MHz).

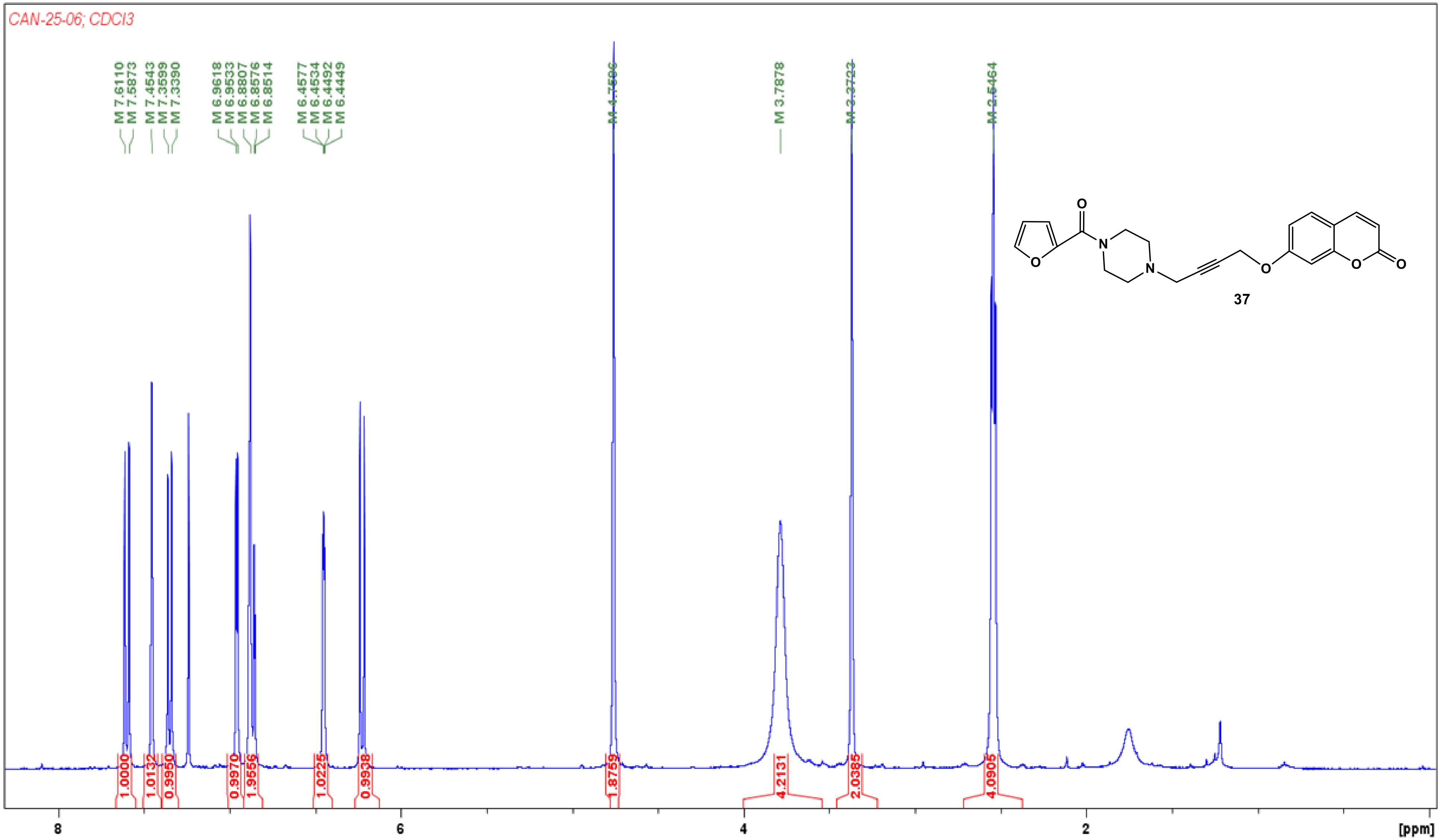

Figure S47. ^{1}H NMR spectrum of 7-(4-(4-(furan-2-carbonyl)piperazin-1-yl)but-2-ynyloxy)-2*H*-chromen-2-one (**37**) ((CDCl$_3$, 300 MHz).

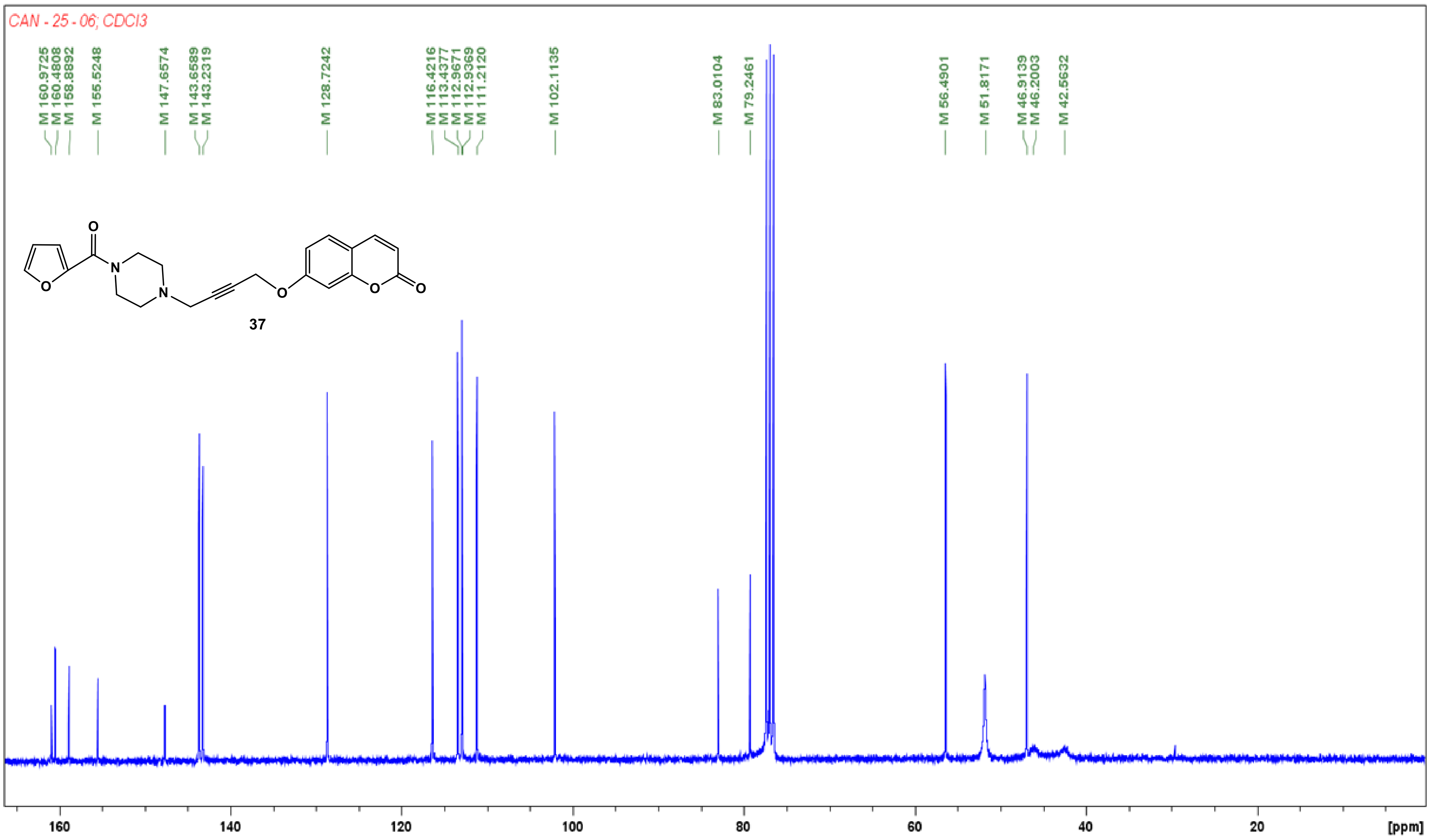

Figure S48. ^{13}C NMR spectrum of 7-(4-(4-(furan-2-carbonyl)piperazin-1-yl)but-2-ynyloxy)-2*H*-chromen-2-one (**37**) ($CDCl_3$, 125 MHz).

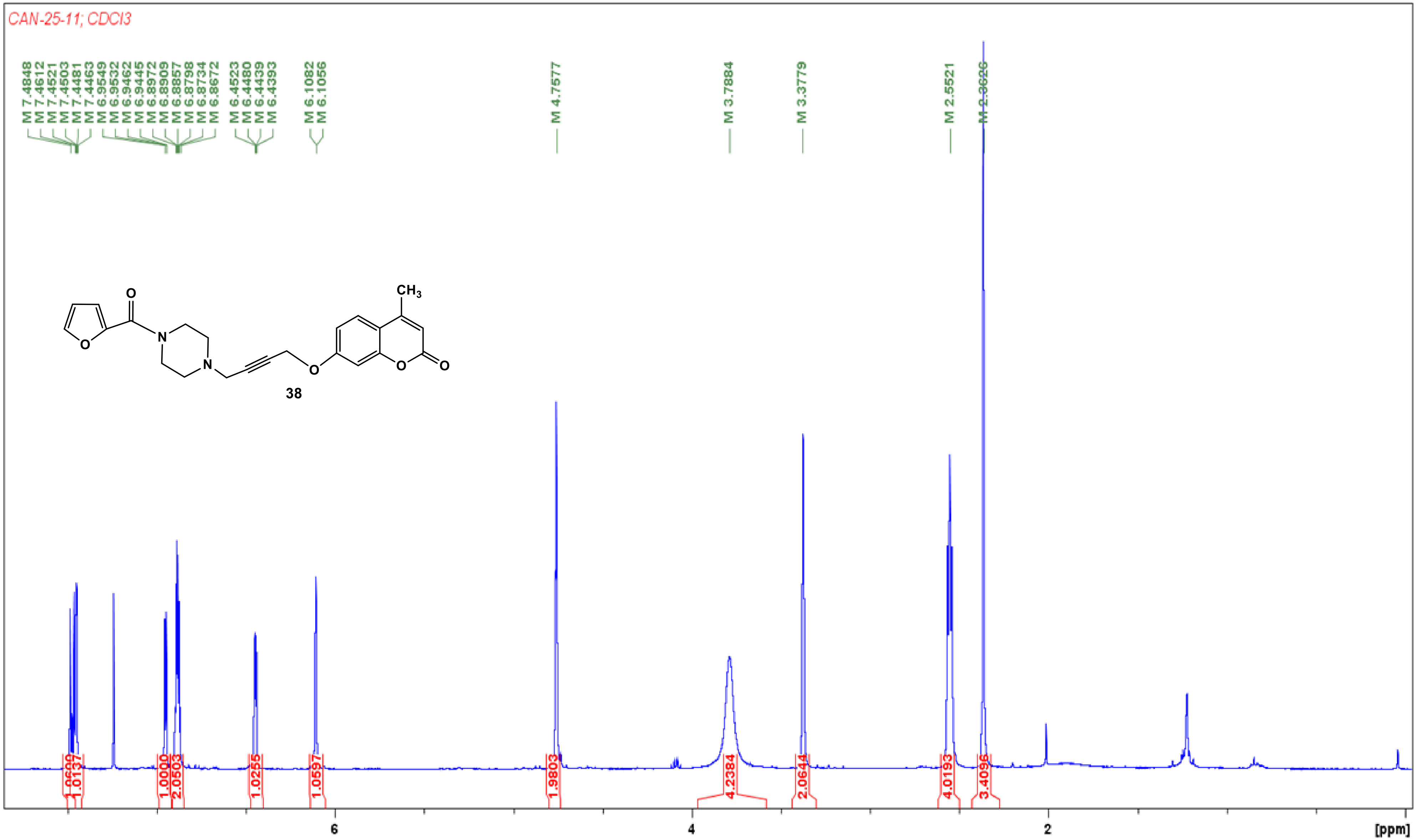

Figure S49. ¹H NMR spectrum of 7-(4-(4-(furan-2-carbonyl)piperazin-1-yl)but-2-ynyloxy)-4-methyl-2*H*-chromen-2-one (**38**) ($CDCl_3$, 300 MHz).

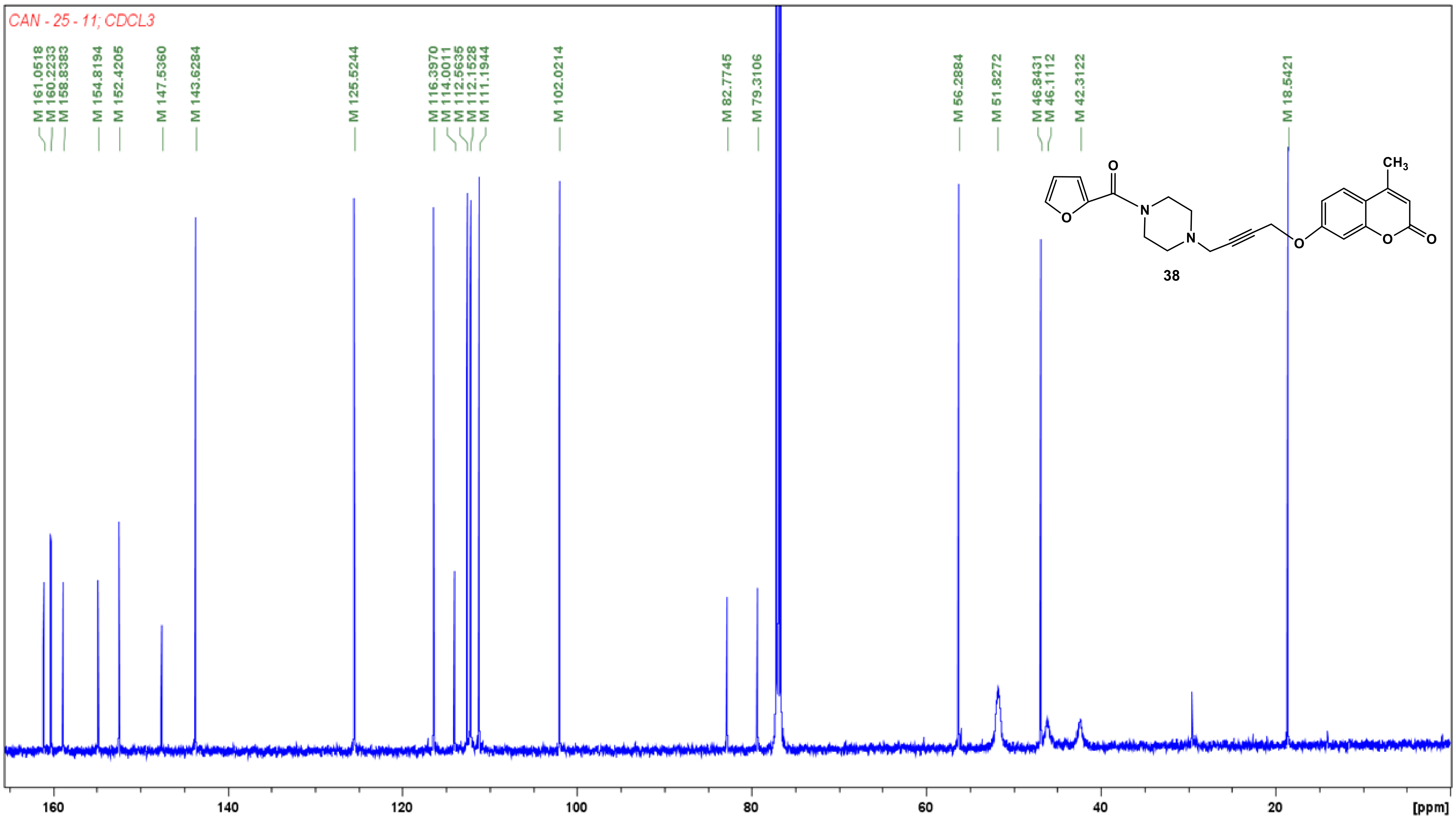

Figure S50. ^{13}C NMR spectrum of 7-(4-(4-(furan-2-carbonyl)piperazin-1-yl)but-2-ynyloxy)-4-methyl-2*H*-chromen-2-one (**38**) ($CDCl_3$, 125 MHz).

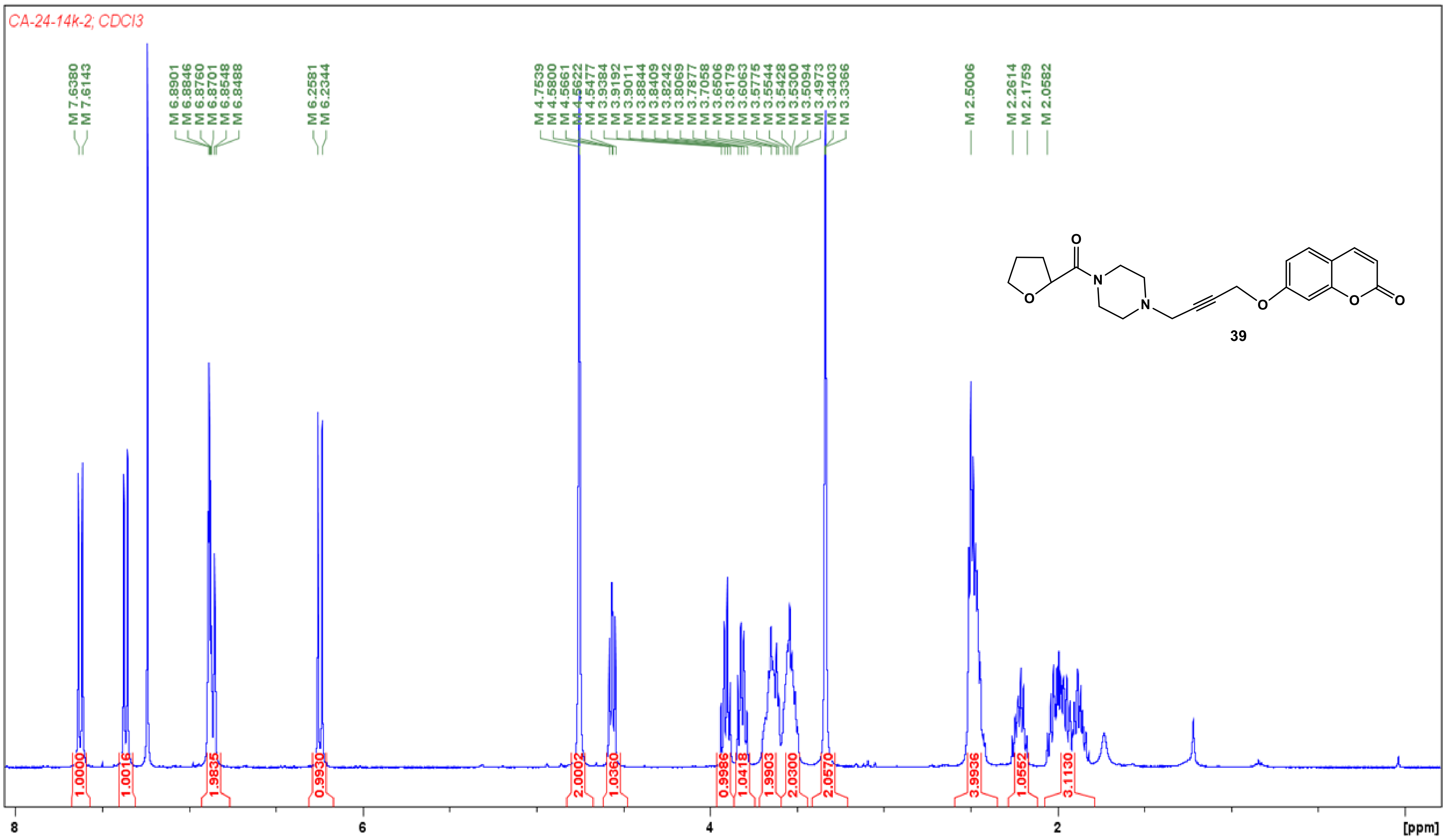

Figure S51. ^{1}H NMR spectrum of 7-(4-(4-(tetrahydrofuran-2-carbonyl)piperazin-1-yl)but-2-ynyloxy)-2*H*-chromen-2-one (**39**) ($CDCl_3$, 300 MHz).

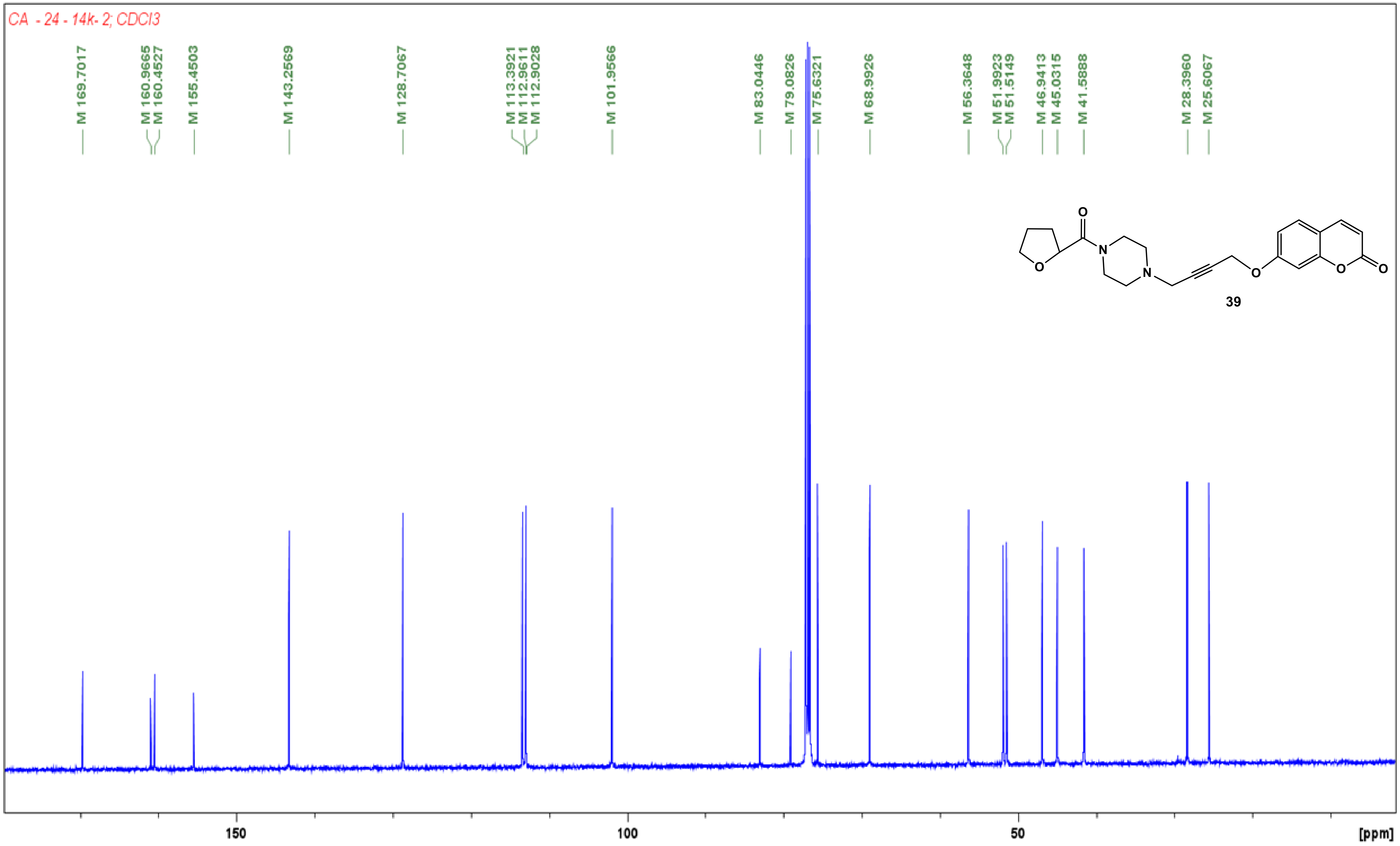

Figure S52. ^{13}C NMR spectrum of 7-(4-(4-(tetrahydrofuran-2-carbonyl)piperazin-1-yl)but-2-ynyloxy)-2*H*-chromen-2-one (**39**) ($CDCl_3$, 125 MHz).

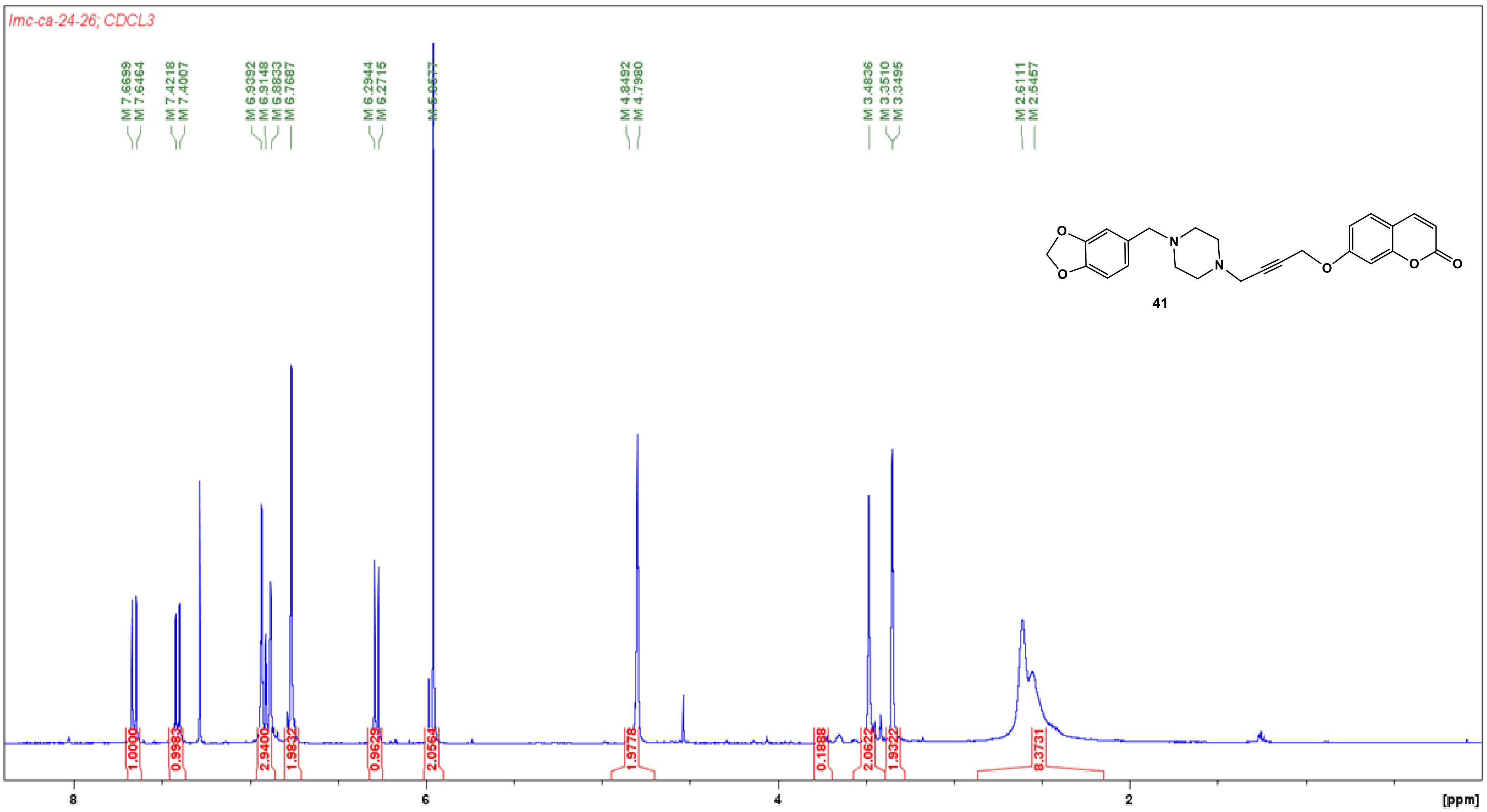

Figure S53. ^{1}H NMR spectrum of 7-(4-(4-(benzo[*d*][1,3]dioxol-5-ylmethyl)piperazin-1-yl)but-2-ynyloxy)-2*H*-chromen-2-one (**41**) ($CDCl_3$, 300 MHz).

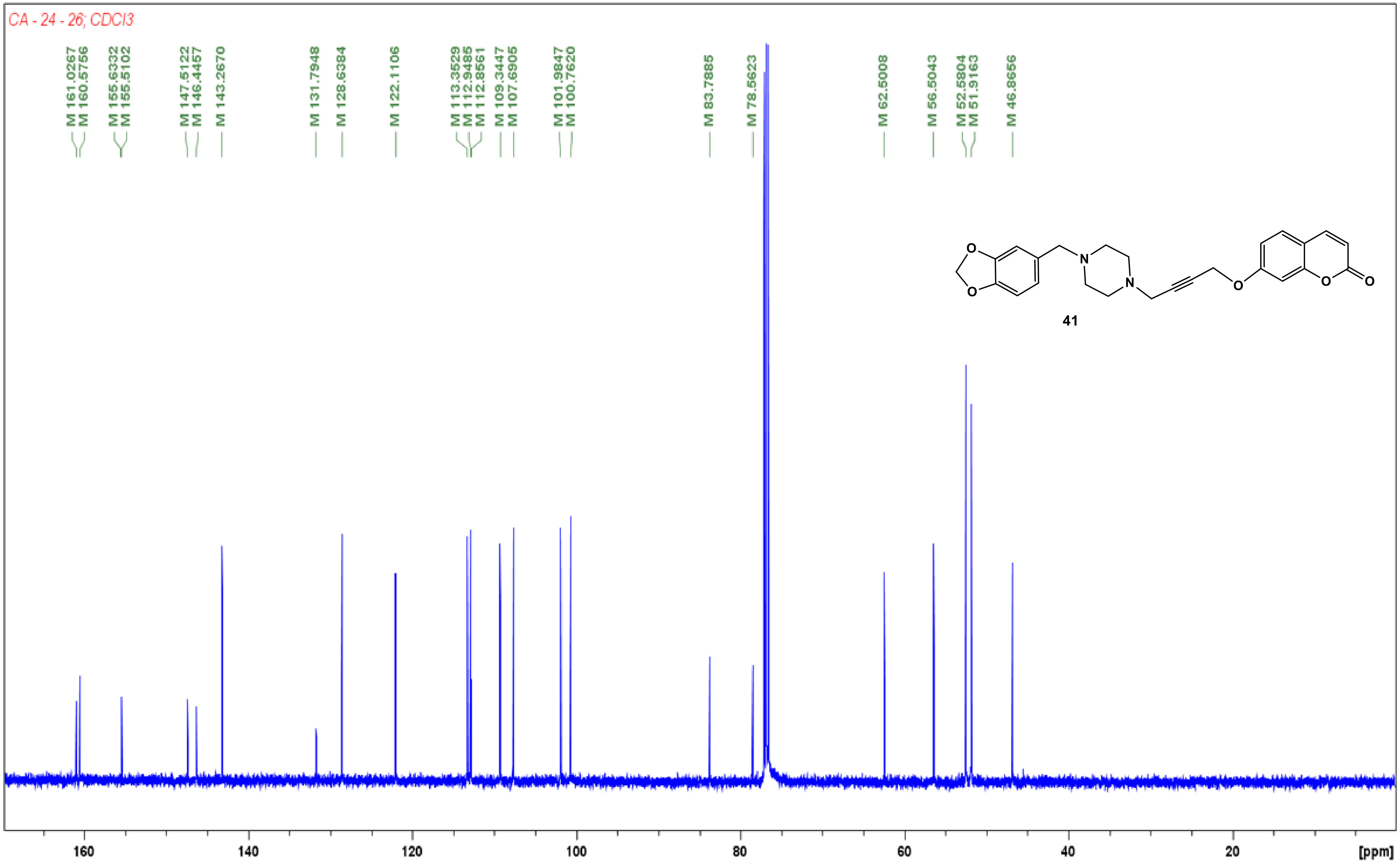

Figure S54. ^{13}C NMR spectrum of 7-(4-(4-(benzo[*d*][1,3]dioxol-5-ylmethyl)piperazin-1-yl)but-2-ynyloxy)-2*H*-chromen-2-one (**41**) ($CDCl_3$, 125 MHz).

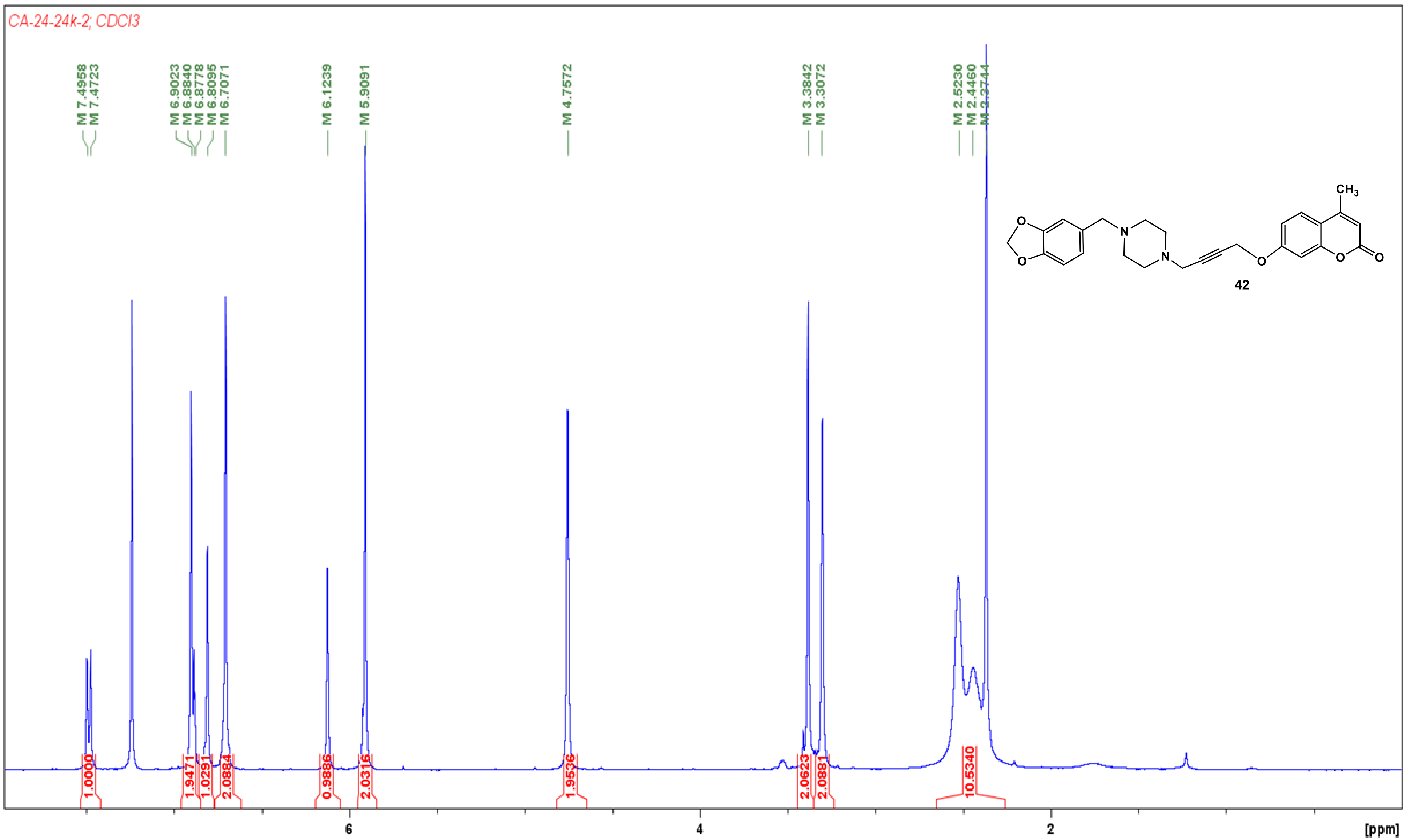

Figure S55. ^{1}H NMR spectrum of 7-(4-(4-(benzo[*d*][1,3]dioxol-5-ylmethyl)piperazin-1-yl)but-2-ynyloxy)-4-methyl-2*H*-chromen-2-one (**42**) ($CDCl_3$, 300 MHz).

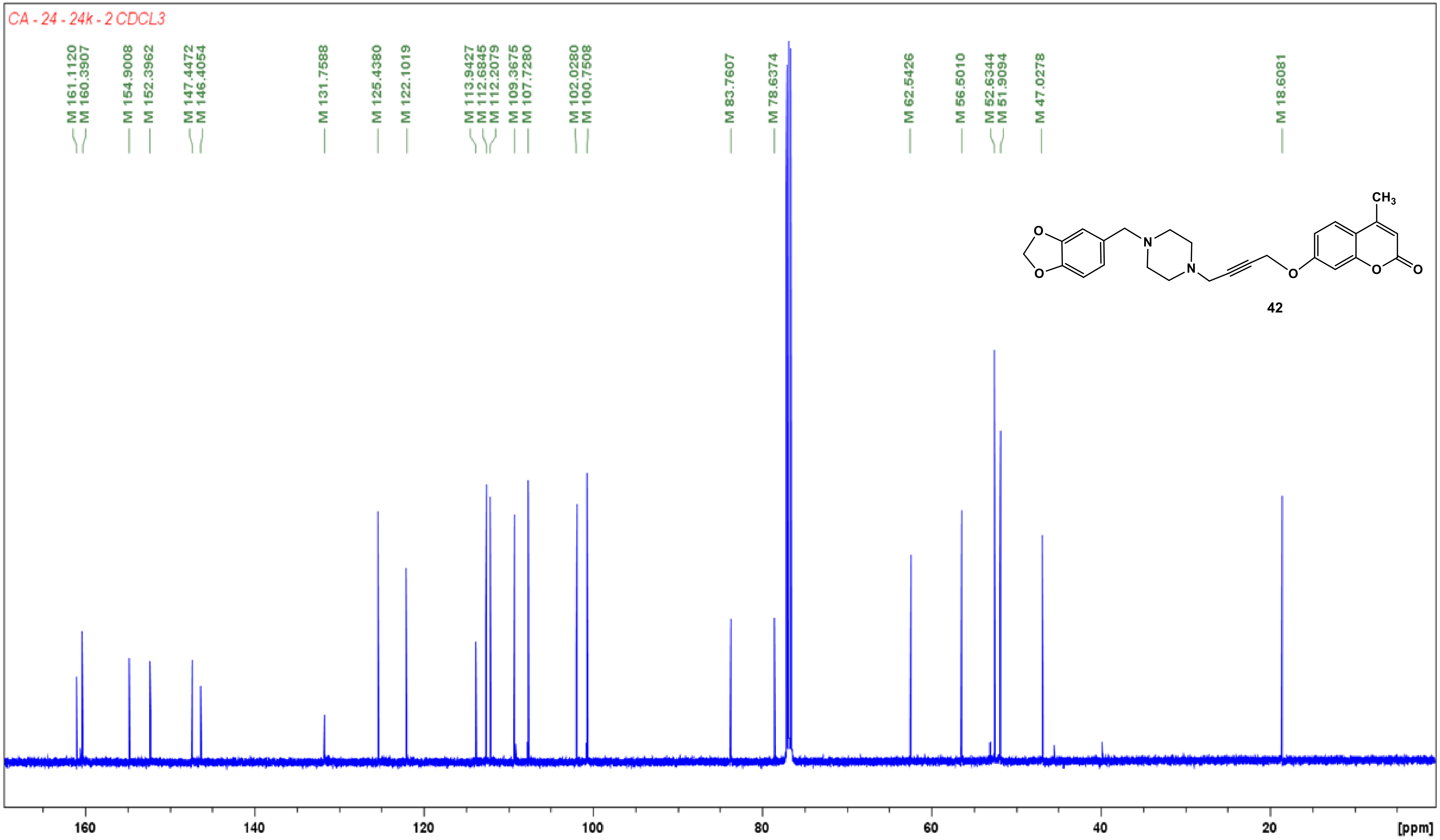

Figure S56. ^{13}C NMR spectrum of 7-(4-(4-(benzo[*d*][1,3]dioxol-5-ylmethyl)piperazin-1-yl)but-2-ynyloxy)-4-methyl-2*H*-chromen-2-one (**42**) ($CDCl_3$, 125 MHz).

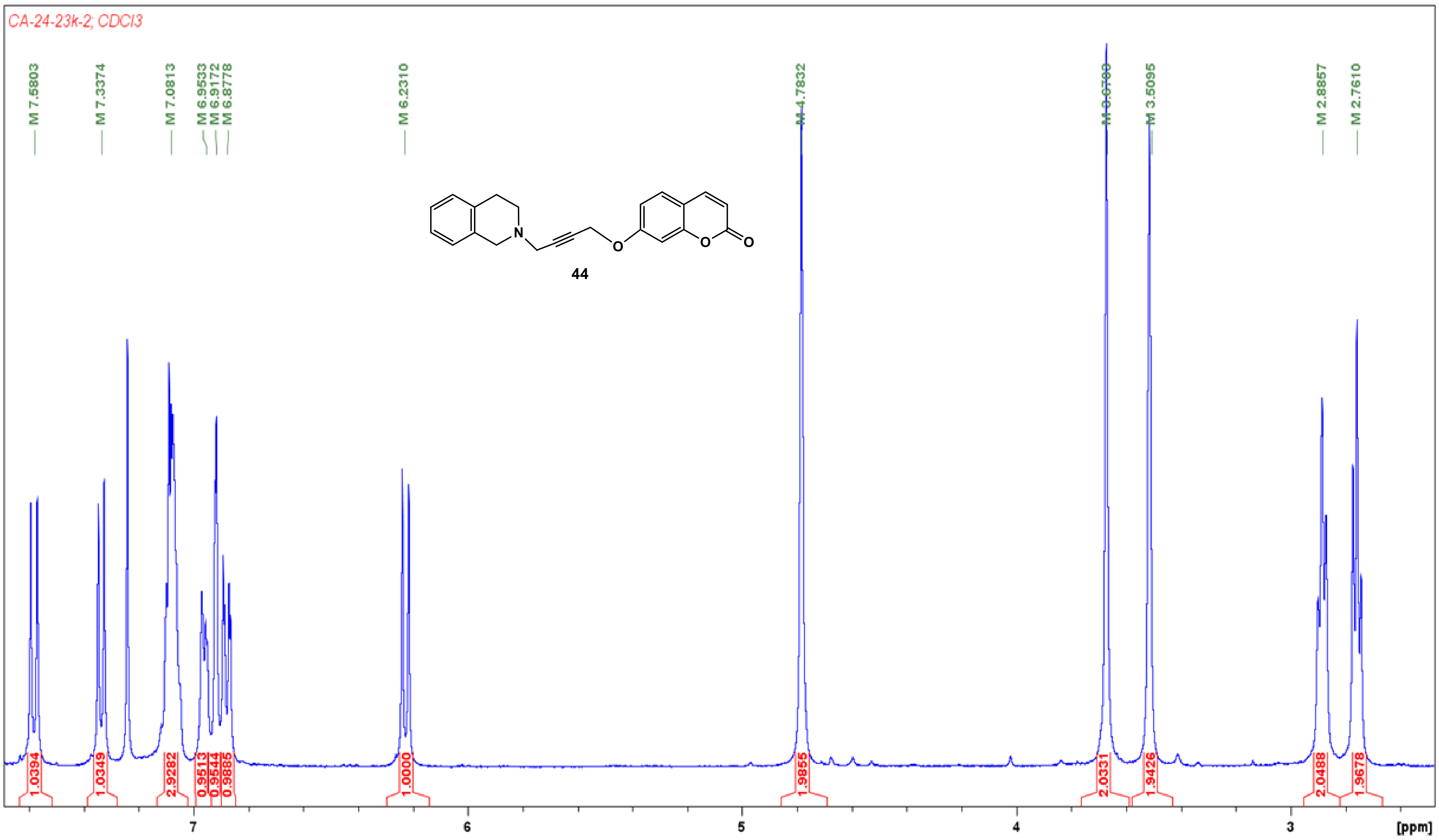

Figure S57. ^{1}H NMR spectrum of 7-(4-(3,4-dihydroisoquinolin-2(1*H*)-yl)but-2-ynyloxy)-2*H*-chromen-2-one (**44**) ($CDCl_3$, 300 MHz).

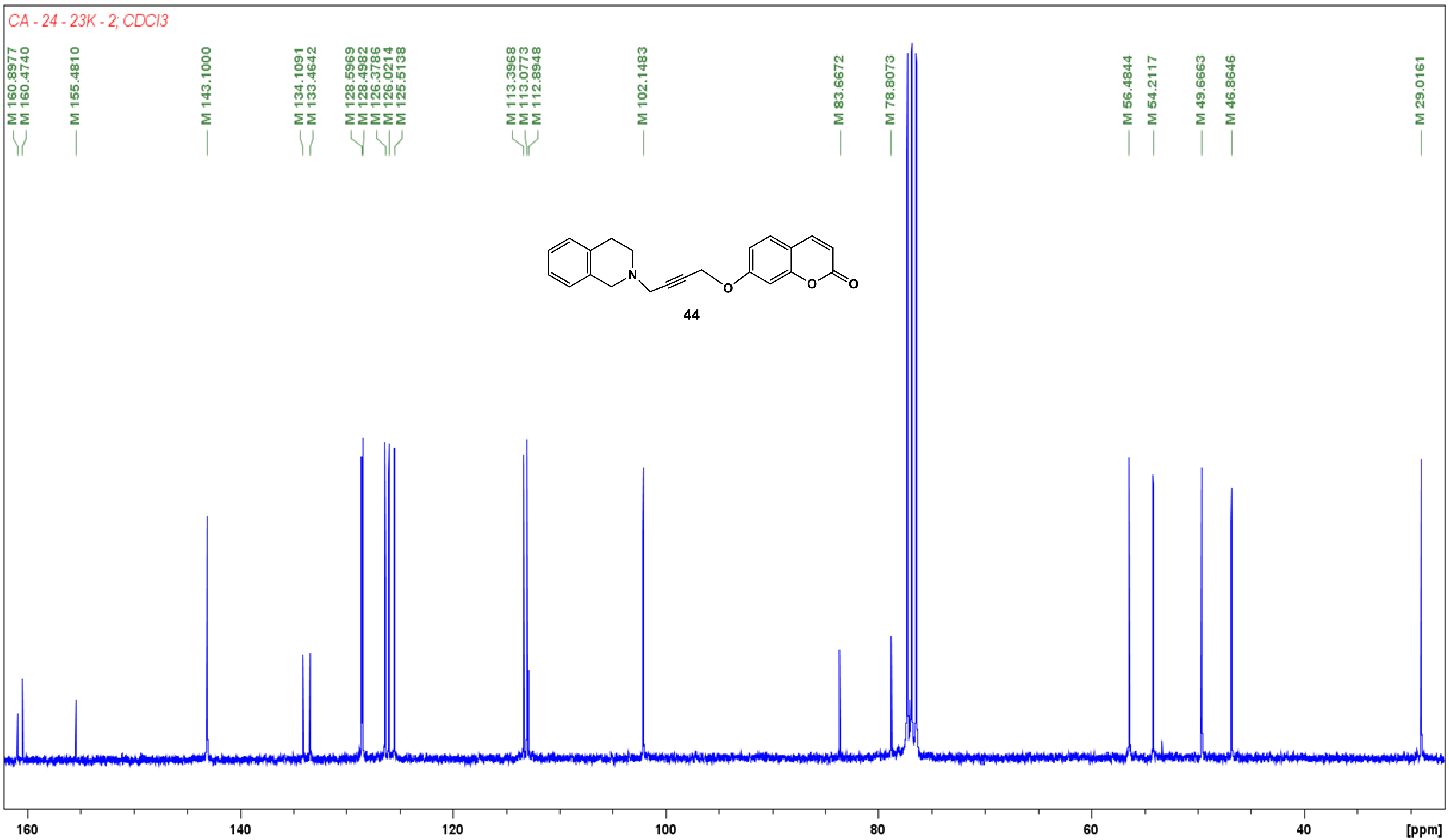

Figure S58. ^{13}C NMR spectrum of 7-(4-(3,4-dihydroisoquinolin-2(1*H*)-yl)but-2-ynyloxy)-2*H*-chromen-2-one (**44**) $CDCl_3$, 125 MHz).

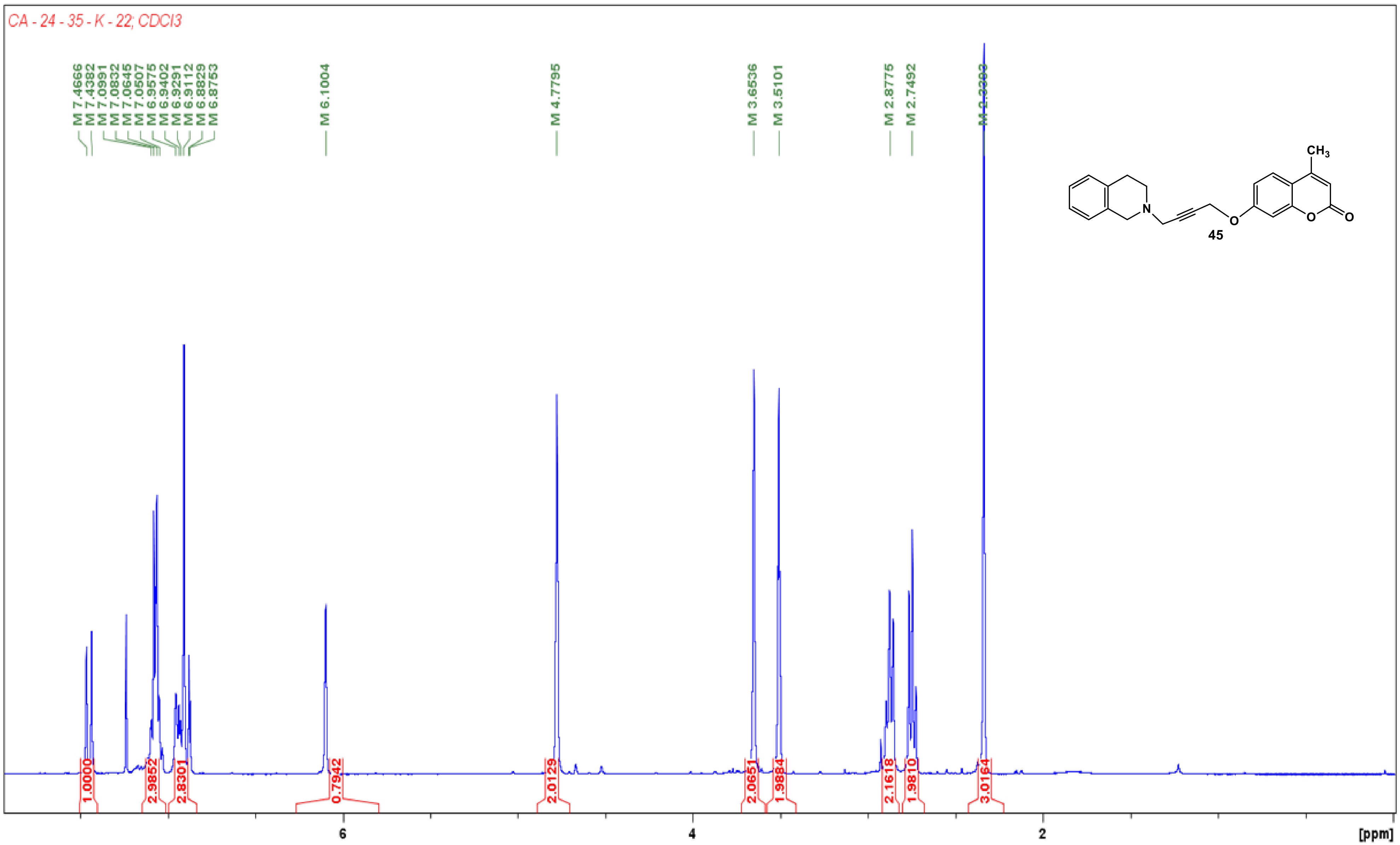

Figure S59. ^{1}H NMR spectrum of 7-(4-(3,4-dihydroisoquinolin-2(1*H*)-yl)but-2-ynyloxy)-4-methyl-2*H*-chromen-2-one (**45**) ($CDCl_3$, 300 MHz).

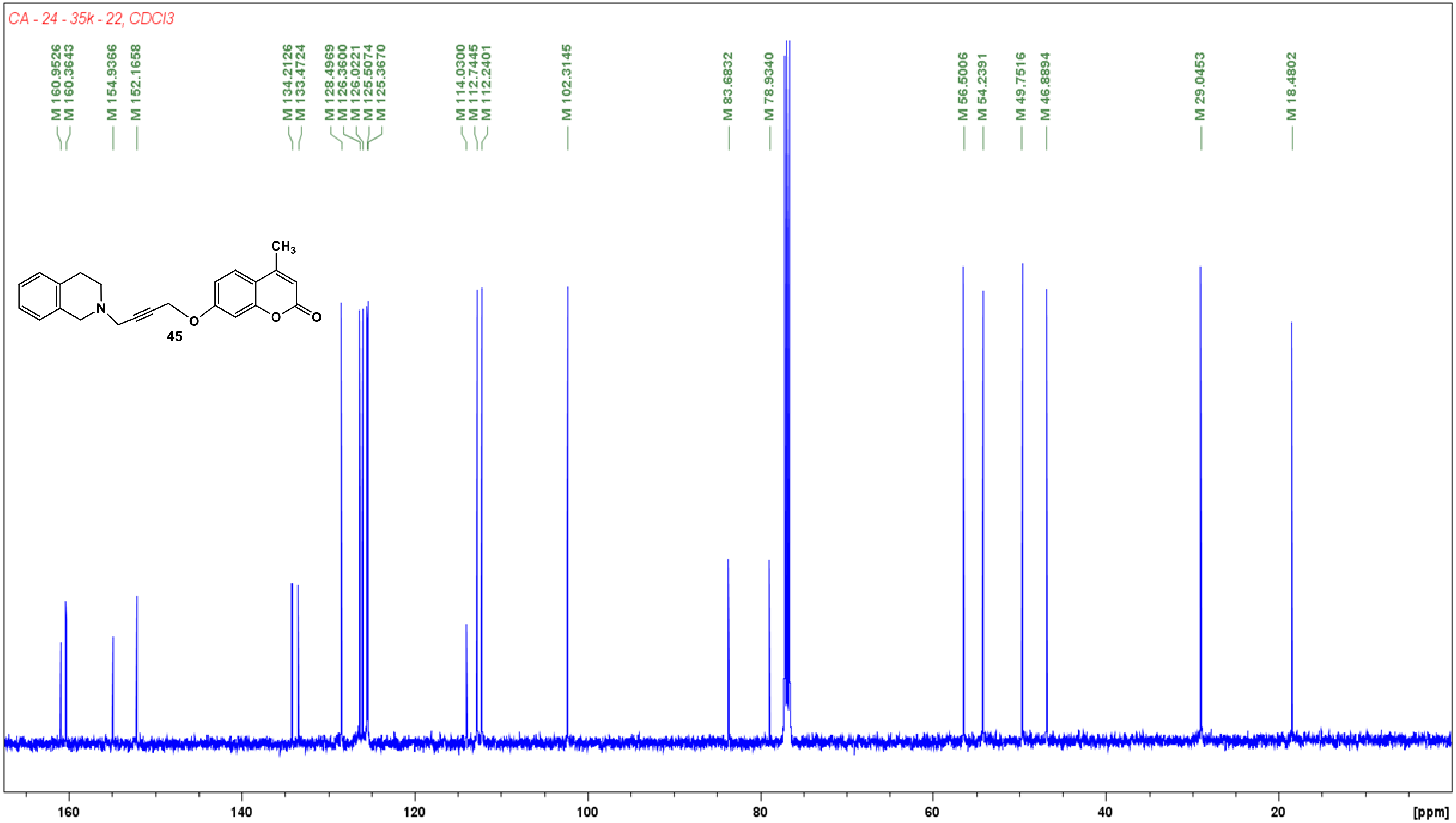

Figure S60. ^{13}C NMR spectrum of 7-(4-(3,4-dihydroisoquinolin-2(1*H*)-yl)but-2-ynyloxy)-4-methyl-2*H*-chromen-2-one (**45**) $CDCl_3$, 125 MHz).
